# Optimising the Flux Enhancer Dosing Strategy in a Pilot-Scale Anaerobic Membrane Bioreactor by Mathematical Modelling

**DOI:** 10.3390/membranes12020151

**Published:** 2022-01-26

**Authors:** Magela Odriozola, Jules B. van Lier, Henri Spanjers

**Affiliations:** Department of Water Management, Delft University of Technology, Stevinweg 1, 2628 CN Delft, The Netherlands; J.B.vanLier@tudelft.nl (J.B.v.L.); H.L.F.M.Spanjers@tudelft.nl (H.S.)

**Keywords:** anaerobic membrane bioreactor (AnMBR), control tool, flux enhancer, integrated model, sludge filterability

## Abstract

Flux enhancers (FEs) have been successfully applied for fouling mitigation in membrane bioreactors. However, more research is needed to compare and optimise different dosing strategies to improve the filtration performance, while minimising the use of FEs and preventing overdosing. Therefore, the goal of this research is to develop an optimised control strategy for FE dosing into an AnMBR by developing a comprehensive integrated mathematical model. The integrated model includes filtration, flocculation, and biochemical processes to predict the effect of FE dosing on sludge filterability and membrane fouling rate in an AnMBR. The biochemical model was based on an ADM1, modified to include FEs and colloidal material. We developed an empirical model for the FE-induced flocculation of colloidal material. Various alternate filtration models from the literature and our own empirical models were implemented, calibrated, and validated; the best alternatives were selected based on model accuracy and capacity of the model to predict the effect of varying sludge characteristics on the corresponding output, that is fouling rate or sludge filterability. The results showed that fouling rate and sludge filterability were satisfactorily predicted by the selected filtration models. The best integrated model was successfully applied in the simulation environment to compare three feedback and two feedforward control tools to manipulate FE dosing to an AnMBR. The modelling results revealed that the most appropriate control tool was a feedback sludge filterability controller that dosed FEs continuously, referred to as $∆R_{20}$_10. Compared to the other control tools, application of the $∆R_{20}$_10 controller resulted in a more stable sludge filterability and steady fouling rate, when the AnMBR was subject to specific disturbances. The simulation environment developed in this research was shown to be a useful tool to test strategies for dosing flux enhancer into AnMBRs.

## 1. Introduction

Anaerobic digestion (AD) is a competitive technology for wastewater treatment, whose main advantages include the conversion of the organic matter into biogas, low sludge production, and no aeration requirement [1]. Anaerobic membrane bioreactor (AnMBR) technology combines AD with membrane filtration to add the advantages of producing a high-quality effluent and to achieving complete solids retention inside the reactor. However, membrane fouling is one of the main operational challenges of AnMBRs.

The most implemented strategies to mitigate fouling in AnMBRs are [2,3]: (1) high shear stress near the membrane surface, (2) increased frequency and duration of backwashing and relaxation, (3) reduced transmembrane flux, and (4) increased frequency of chemical cleaning. The first strategy is achieved through increased biogas sparging or crossflow velocity, which are energy intensive processes that cause an increased energy demand. The second and third strategies decrease the treatment capacity, namely the daily volume of wastewater treated. The fourth strategy reduces the lifespan of the membranes. Alternative fouling mitigation strategies have been reported, such as the use of scouring agents, dosing flux enhancers (FEs), the use of vibrating/rotating membranes, and application of an electric field by using microbial electrolysis cell [3]. Particularly, cationic polymers have been successfully applied as FE in membrane bioreactors fed with real wastewater [4,5,6,7,8,9,10,11,12,13] and synthetic wastewater [14,15,16,17,18,19,20]. However, despite some successful applications, a proper FE dosing control tool is lacking, and different empirically based approaches are followed. In most cases, FE dosage to membrane bioreactors follows a ”feedforward dosing” strategy, as defined in Odriozola et al. [13]. Feedforward dosing does not consider possible disturbances and is based on different assumptions related to possible FE losses and target optimal FE concentrations, which in effect might lead to FE underdose or overdose. ”Feedback dosing” might be a good alternative to avoid underdosing and overdosing because the FE dosage is adjusted based on an input variable that quantifies the sludge filtration characteristics [21,22]. Therefore, feedback dosing can reject possible disturbances and does not require the assumptions made in feedforward dosing. Despite the possible advantages of feedback dosing, to the best of our knowledge, the only published study that used it was by Alkmim et al. [12], who manually performed a feedback dosing strategy, using time-to-filter measurements to assess the sludge filtration properties and dosing a pulse of FEs when the time-to-filter exceeded 200 s^−1^. The authors compared feedback and feedforward dosing, which they referred to as corrective and preventive dosage, respectively. Feedforward dosing caused more stable time-to-filter data and used a lower amount of FEs than feedback dosing. However, the comparison between dosing strategies was challenging due to differences in operational conditions. For example, during the feedforward dosing assessment, sludge was lost due to a leakage that might have caused FE loss which could explain the higher amount of FE used. Therefore, more research is needed to compare and optimise different dosing strategies to improve the filtration performance, while minimising the use of FEs and avoiding overdosing.

Application of a proper FE dosing strategy in membrane bioreactors requires the presence of reliable input data that activates a control tool. Various researchers have suggested the application of the online measurement of sludge filtration characteristics as an input variable [21,22]. Accordingly, in situ measurement of sludge filterability, measured with the anaerobic Delft filtration characterization method (AnDFCm) mounted in parallel, have proven to be an appropriate input variable for manipulating FE dosage for fouling control in a pilot-scale AnMBR [13]. Moreover, a control tool that couples sludge filtration characteristics measurements (such as filterability) with the membrane filtration process state variables (such as fouling rate) could identify the actual cause of changes in filtration performance and decide on the appropriate intervention [13,23], which is not necessarily restricted to FE dosing.

Comparing various FE dosing strategies using experiments, independent of the scale, can be expensive and time-consuming. Additionally, dosing strategies should ideally be compared under identical operational conditions, which is challenging to achieve in different reactors in parallel or in the same reactor at different moments. A simulation environment is regarded as an effective tool to test various FE dosing strategies for fouling control, provided the model structure is commonly accepted. The simulation environment should have a comprehensive model that can predict the effect of FE dosing on membrane fouling rate and sludge filterability. However, thus far, such a comprehensive model has never been presented in the literature. Nevertheless, multiple models with diverse complexity have been discussed for the different processes involved in membrane bioreactors, including filtration, biochemical, hydrodynamic, and flocculation models [3,24,25]. These models can be adapted and coupled into a comprehensive integrated model that describes an AnMBR under FE dosage. The first step towards developing such an integrated model is to identify the variables that are affected by the FE dosage and influence the filtration performance.

Colloidal material has been consistently identified as a major factor that affects sludge filterability [26] and reversible fouling in membrane bioreactors [13,27,28,29,30,31,32]. Colloidal material deposited in the cake layer can decrease cake porosity by filling the void space of the cake. The concentration of colloidal material decreases after dosing cationic polymers as FEs, while the floc size increases [13,26]. Regarding floc size, larger flocs can form more porous cakes, reduce the adhesion of the flocs to the membrane, increase the back transport of flocs from the membrane surface to the bulk liquid, and reduce cake layer thickness by surface erosion [27,33,34,35], thus, decreasing membrane fouling. However, the effect of floc size on membrane fouling is controversial [13]. Floc size can have a substantial effect on membrane fouling for small flocs, whereas further increasing the size of already large particles might have a negligible effect on fouling mitigation. Considering the above, the colloidal material concentration is likely an appropriate state variable to describe the effect of FE dosing on the membrane fouling rate and sludge filterability, whereas floc size may not be an appropriate variable. The concentration of particulate material is a poor indicator of sludge fouling propensity by itself [28]. However, it is a crucial input variable in filtration models because it affects cake layer formation by particle deposition over the membrane surface and may play a role in scavenging colloidal material [36]. The concentration of particulate material is affected by biochemical processes (such as bioconversion, biomass growth, and decay), influent characteristics, and flocculation of colloidal material. Therefore, to predict the effect of FE dosing on membrane fouling rate and sludge filterability, an integrated model including filtration with colloidal material deposition, (FE-induced) flocculation, and biochemical processes is needed.

The IWA anaerobic digestion model No. 1 (ADM1) [37] has been widely applied to simulate the biochemical processes occurring in anaerobic reactors [3,38,39], including AnMBRs. Other biochemical models applied in AnMBR include the biological nutrient removal model No. 2 (BNRM2) [40] and the simple anaerobic model AM2b that incorporates the kinetics of soluble microbial products (SMP) [41]. Although anaerobic digestion modelling is a relatively mature field, the kinetics of colloidal material has not yet been incorporated. Moreover, the FE could have a detrimental effect on the biological activity [42]. Therefore, to model an FE-dosed AnMBR, the biochemical models should be extended to incorporate colloidal material and FEs.

The filtration process in membrane bioreactors mostly has been modelled with grey box models, particularly, by applying the resistance-in-series model, Darcy’s Law, drag and lift forces, and the Carman–Kozeny equation to predict the membrane performance (such as fouling rate, transmembrane pressure, and transmembrane flux) based on sludge characteristics and operating conditions [3,24,25]. Researchers have modelled the reduction in cake layer porosity, and consequently, the increase in the specific cake resistance (SCR), caused by the entrapment of colloidal material [43], extracellular polymeric substances (EPS) [44], SMP [45,46], and submicron material [47]. This could be an appropriate approach to incorporate the effect of FE dosing on membrane fouling and sludge filterability through changes in the concentration of colloidal material.

To model the flocculation process, population balance models (PBM) have been widely applied in chemical engineering to predict the particle size distribution [48]. However, incorporating PBM in an AnMBR integrated model results in an extremely complex model with many state variables. Alternatively, a simpler flocculation model that describes the dynamics of floc size (i.e., mean particle size) can be useful and are sufficient to assess the necessity of floc size as a linking variable between a biochemical-flocculation model and filtration models.

The objective of this research is to develop an optimised control strategy for FE dosing into an AnMBR by developing a comprehensive integrated mathematical model. The integrated model was calibrated and validated using previously published data from a pilot-scale AnMBR treating blackwater [13,26]. The integrated model was used as simulation environment to test five control tools for manipulating FE dosing to an AnMBR. The developed integrated model includes flocculation, filtration, and biochemical processes to predict the effect of FE dosing on sludge filterability (as measured with the AnDFCm) and membrane fouling rate in an AnMBR. The tested control tools were: two feedback sludge filterability controllers, two feedforward FE concentration controllers, and one feedback FE concentration controller.

## 2. Materials and Methods

### 2.1. Experimental Data

The models were calibrated and validated using previously published data from a pilot-scale AnMBR plant treating blackwater from the main office building of the Business Centre Porto do Molle, Nigrán, Pontevedra, Spain. The experimental data consisted of monitoring data from the continuous operation of the pilot dosed with FE [13], and FE dosage-step experiments with grab samples [26]. During the operation of the pilot, a pulse input of the cationic polymer Adifloc KD451 (Adipap SA, Versailles, France) was introduced to the bypass line on Day 16 (see [13]). Sludge filterability, based on the anaerobic Delft filtration characterisation method (AnDFCm) [26], was measured ex situ during the dosage-step tests [26], and in situ during the pilot operation by connecting the AnDFCm installation in bypass to the pilot [13]. The output of the AnDFCm is the additional resistance obtained when 20 L of permeate per m^2^ of membrane surface area are produced, denoted as $∆R_{20}$; the sludge filterability is inversely related with $∆R_{20}$. Further details about the pilot-scale AnMBR are described in Odriozola et al. [13], and Section S1 presents complementary information, experimental results, and procedures for data treatment relevant to this research.

Furthermore, two additional experiments were performed to estimate certain parameter values. The description and results of these experiments are presented in Section S2. In short, batch flocculation kinetic experiments were performed to estimate the FE adsorption rate coefficient ($k_{\mathrm{ads}}$), and sludge viscosity measurements were performed in the AnDFCm installation to estimate the parameters of the mixed liquor viscosity model, a and b in Equation (36), for the AnDFCm filtration model. The dynamic viscosity of the mixed liquor ($\mu_{L}$) was calculated using the experimentally measured pressure drop along the membrane in the AnDFCm installation [49,50], while sludge samples with different total suspended solids (TSS) concentrations were used.

### 2.2. General Model Description and Approach

**Figure 1:**
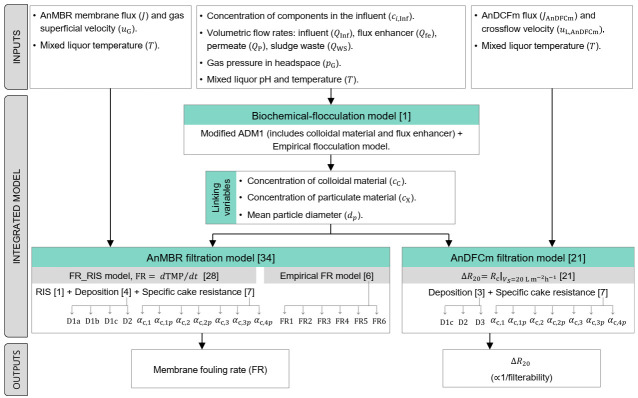
Modelling approach scheme. Between square brackets is the number of compared alternate models to select the most appropriate model. Abbreviations: ADM1, anaerobic digestion model No. 1; AnDFCm, anaerobic Delft filtration characterization method; RIS, resistance-in-series; SCR, specific cake resistance; TMP, transmembrane pressure; D1a, D1b, D1c, D2, and D3 are alternate deposition submodels; $\alpha_{c,1}$, $\alpha_{c,1p}$, $\alpha_{c,2}$, $\alpha_{c,2p}$, $\alpha_{c,3}$, $\alpha_{c,3p}$, and $\alpha_{c,4}$ are alternate SCR submodels; and FR1 to FR6 are alternate empirical FR models.

The required submodels for developing the control tool for an optimised FE dosing strategy are overviewed in Figure 1. The main outputs of the integrated model are the AnMBR membrane fouling rate and the sludge filterability expressed as $∆R_{20}$. The integrated model couples a biochemical-flocculation model with two filtration models: one for the AnMBR membrane module and one for AnDFCm installation membrane. The biochemical-flocculation model predicts the sludge characteristics that are used as input in both filtration models. For the AnMBR and AnDFCm filtration models, we compared several alternate models to select the best-fitting ones.

The AnMBR filtration process was modelled using two alternate approaches: (1) FR_RIS model and (2) empirical FR model. In the FR_RIS model, the fouling rate (FR) is calculated as the change in transmembrane pressure (TMP) over time during each filtration cycle (dTMP/dt). The TMP is calculated by combining three submodels: resistance-in-series (RIS), deposition, and SCR. The deposition submodel is an ordinary differential equation system to describe the deposition of colloidal and particulate material onto the membrane. The SCR submodel is an equation to calculate the SCR based on the amount and characteristics of the material deposited onto the membrane. We compared 28 alternate FR_RIS models that result from all possible combinations among: four alternate deposition submodels (Section 2.4.3), seven alternate SCR submodels (Section 2.4.2), and one RIS submodel (Section 2.4.1). The empirical FR model is an algebraic equation to calculate FR directly from the operational variables and mixed liquor properties, we proposed six alternate empirical FR models (Section 2.5). Therefore, 34 alternate AnMBR filtration models were compared, i.e., 28 FR_RIS and 6 empirical FR models.

In the AnDFCm filtration model the $∆R_{20}$ is the resistance of the cake layer ($R_{c}$) after 1200 s of continuous filtration under the operational conditions of the AnDFCm. $R_{c}$ is calculated by combining deposition and SCR submodels. We compared 21 alternate AnDFCm models (Section 2.6) that result from combinations of three deposition and seven SCR alternate submodels.

### 2.3. Biochemical-Flocculation Model

The biochemical-flocculation model is an amended ADM1 [37], modified to include the following processes caused by FE dosing: adsorption of FE onto particulate material, flocculation of colloidal material, and change in mean particle size as a result of flocculation. The model includes three new components as follows: inert colloidal material ($C_{I}$), FE in the bulk liquid (soluble, $S_{\mathrm{fe}}$), and adsorbed FE ($X_{\mathrm{fe}}$). Section S3 displays the scheme of the modified ADM1 and the stoichiometric (Petersen) matrix and process rate equations.

The modelled FE is a cationic polymer that interacts with the negatively charged surface of particulate and colloidal material. The adsorption of FE onto particulate material is described with the pseudo-first order model [51] in Equation (1), the FE adsorption onto particulate material promoted the flocculation of colloidal material as described in Equation (2):

(1) $$\rho_{23}=k_{\mathrm{ads}}\left(X_{\mathrm{fe},e}-X_{\mathrm{fe}}\right),$$

(2) $$\rho_{24}=Y_{\mathrm{fe},C}\;k_{\mathrm{ads}}\left(X_{\mathrm{fe},e}-X_{\mathrm{fe}}\right)\frac{\;C_{I}/i_{\mathrm{COD},\mathrm{CI}}}{c_{X}+c_{C}}\frac{X_{I}}{X_{I}+1\times 10^{-6}},$$

where $k_{\mathrm{ads}}$ is the adsorption rate coefficient; $X_{\mathrm{fe},e}$ is the adsorbed concentration of FE after equilibrium; $X_{\mathrm{fe}}$ is the concentration of FE adsorbed onto particulate material; $Y_{\mathrm{fe},C}$ is the yield of colloidal material flocculated per unit of FE adsorbed onto the particulate material; $C_{I}$ is the concentration of colloidal inert; $X_{I}$ is the concentration of particulate inert; $i_{\mathrm{COD},\mathrm{CI}}$ is the theoretical chemical oxygen demand (COD) for $C_{I}$; and $c_{C}$ and $c_{X}$ are the total concentration of colloidal and particulate material, respectively, expressed as suspended solids, and calculated as follows:

(3) $$c_{C}=\sum_{i}C_{i}/i_{\mathrm{COD},i},$$

(4) $$c_{X}=\sum_{i}X_{i}/i_{\mathrm{COD},i},$$

where $i_{\mathrm{COD},i}$ is the theoretical chemical oxygen demand for component i. 

Through Equation (2) the model considers the deflocculation of $X_{I}$ into $C_{I}$ when the concentration of FE decreased, and thus, the last term in Equation (2) was introduced to avoid negative $C_{I}$ values when $X_{I}$ approaches zero and there is no material to be deflocculated.

To calculate $X_{\mathrm{fe},e}$, the Langmuir adsorption isotherm in Equation (5), which describes the equilibrium conditions, is combined with Equation (6), which is the mass balance of FE inside the reactor at equilibrium conditions:

(5) $$X_{\mathrm{fe},e}=q_{m,\mathrm{ads}}\;c_{X}\frac{K_{L,\mathrm{ads}\;}S_{\mathrm{fe},e}}{1+K_{L,\mathrm{ads}\;}S_{\mathrm{fe},e}}\;,$$

(6) $$S_{\mathrm{fe},e}=\left(c_{\mathrm{fe}}-X_{\mathrm{fe},e}\right),$$

where $q_{m,\mathrm{ads}}$ is the maximum adsorption capacity corresponding to monolayer coverage, $K_{L,\mathrm{ads}}$ is the Langmuir affinity coefficient, $S_{\mathrm{fe},e}$ is the concentration of FE in the bulk liquid after equilibrium, and $c_{\mathrm{fe}}$ is the total concentration of FE inside the reactor.

Hydrolysis of decayed biomass has been reported as the slowest process in anaerobic digestion [52]. Thus, we modified the ADM1 approach and decoupled the degradation rates of the decayed biomass and of the particulate material of the influent. For this, the model incorporates the disintegration of decayed biomass as the rate limiting process in biomass degradation, whereas the particulate material of the influent directly hydrolyses (without disintegration).

The following additional modifications and assumptions in ADM1 were made: (1) removal of ammoniacal nitrogen inhibition of acetoclastic methanogenesis, because it is negligible in the pH range 7.0–7.5 and at total nitrogen concentrations in the permeate of 80–200 mgN L^−1^, as measured in the pilot-scale AnMBR [53]; (2) inclusion of a non-competitive inhibition of acetoclastic methanogenesis by FE ($I_{\mathrm{fe},\mathrm{ac}}$) [42]; and (3) inclusion of pH as an input of the model instead of performing the ion balance because pH was measured online by the supervisory control and data acquisition (SCADA) system.

Our model assumes that all soluble components pass through the membrane and reach the permeate, whereas the colloidal and particulate components are retained by the membrane and remain inside the reactor. Equation (7) gives the mass balances of component i in the liquid phase, and Equation (8) the mass balances of component i in the gas phases:

(7) $$\frac{dc_{i}}{dt}=E_{i}-f_{i,\mathrm{WS}}\frac{c_{i}Q_{\mathrm{WS}}}{V_{L}}-f_{i,P}\frac{c_{i}Q_{P}}{V_{L}}+\frac{1}{t_{\mathrm{conv}}}\sum_{j=\left[1-23\right]}\upsilon_{i,j}\rho_{j},$$

(8) $$\frac{dc_{i,G}}{dt}=-\frac{c_{i,G}Q_{G}}{V_{G}}+\frac{V_{L}}{t_{\mathrm{conv}}\;V_{G}}\sum_{j=\left[19-21\right]}\upsilon_{i,j}\rho_{j},$$

where $E_{i}$ is the input function of component i; $f_{i,P}$ is the fraction of component i that passes through the membrane and reaches the permeate ($f_{i,P}$ = 0 for colloidal and particulate components and $f_{i,P}$ = 1 for soluble components); $f_{i,\mathrm{WS}}$ is the fraction of component i that leaves the reactor with the sludge waste ($f_{i,\mathrm{WS}}$ = 1 for all components); $\upsilon_{i,j}$ is the stoichiometric coefficient of component i in process j and $\rho_{j}$ the rate of process j; $t_{\mathrm{conv}}$ the time conversion factor (86,400 s d^−1^); $c_{i,G}$ is the concentration of component i in the gas phase; $V_{L}$ is the total mixed liquor volume; and $V_{G}$ the volume of the gas phase in the reactor. $E_{i}$ was calculated with the concentration of component i in the influent ($c_{i,\mathrm{Inf}}$), Equation (9). 

The FE was added to the reactor in a separate flow, which increased the concentration of FE in bulk liquid ($S_{\mathrm{fe}}\equiv S_{13}$):

(9) $$E_{i}=\left\{\begin{array}{c} \frac{c_{i,\mathrm{Inf}\;}Q_{\mathrm{Inf}}}{V_{L}},\;\;i=\left[1,12\right]\;\cup \;\left[14,26\right]\\ \;\;\frac{{\dot{m}}_{\mathrm{fe}}}{V_{L}},\;\;\;\;i=13 \end{array}\right..$$

In the pilot-scale AnMBR (described in Section S1), the mean particle diameter ($d_{p}$) was almost constant throughout the operational period without dosing FE. We referred to this mean size as the mean particle diameter at stable operation ($d_{p,\mathrm{St}}$). Moreover, immediately after dosing FE to the pilot, $d_{p}$ increased, and then it decreased continuously until it reached $d_{p,\mathrm{St}}$. Therefore, we proposed the empirical model in Equation (10) to describe the $d_{p}$ dynamics in the pilot AnMBR:

(10) $$\frac{dd_{p}}{dt}=\frac{1}{t_{\mathrm{conv}}}\left(k_{\mathrm{floc},\mathrm{fe}}\frac{dX_{\mathrm{fe}}}{dt}+\left(d_{p,\mathrm{St}}-d_{p}\right)k_{\mathrm{floc}}\right),$$

where $k_{\mathrm{floc}}$ is the empirical flocculation-deflocculation rate that represents aggregation and breakage, and $k_{\mathrm{floc},\mathrm{fe}}$ is the FE-induced flocculation yield. The first term represents the immediate increase after FE dosing, where $d_{p}$ changes linearly with the adsorbed FE concentration, as experimentally observed in dosage-step tests with grab sludge samples [26]. The second term of the equation represents the tendency of $d_{p}$ to reach $d_{p,\mathrm{St}}$ in the pilot-scale AnMBR.

### 2.4. AnMBR Filtration: Alternate FR_RIS Models

The AnMBR FR_RIS filtration models predict the cake layer formation by attachment and detachment of particulate material onto the membrane, and the SCR $\left(\alpha_{c}\right)$ by entrapment of colloidal material in the cake layer. The model output is fouling rate (FR) calculated with a linear regression model using the simulated TMP, Equation (S.5) in Section S1.

#### 2.4.1. Resistance-In-Series (RIS) Submodel

The TMP was calculated by Darcy’s law, as follows:

(11) $$\mathrm{TMP}=J\mu R_{t},$$

where $R_{t}$ is the total filtration resistance, μ is the dynamic viscosity of the permeate, and J is the transmembrane flux. The permeate viscosity was assumed to be equal to pure water viscosity and calculated at the measured temperature (T, K) with the following empirical relationship [54]:

(12) $$\mu =0.001\exp\left(0.580-2.520\;\theta +0.909\;\theta^{2}-0.264\;\theta^{3}\right),\mathrm{with}\;\theta =\frac{3.661\;\left(T-273.1\right)}{273.1}.$$

Although no chemical cleaning was performed in the pilot-scale AnMBR during 2 years of operation, no irreversible fouling was observed. Therefore, the irreversible fouling resistance was neglected, and $R_{t}$ was calculated with the following RIS submodel:

(13) $$R_{t}=R_{m}+R_{c},$$

where $R_{m}$ is the intrinsic resistance of the membrane and $R_{c}$ is the cake-layer resistance. $R_{c}$ is the product between the mass of particulate material deposited per membrane area ($\omega_{X}$) and the SCR $\left(\alpha_{c}\right)$, as follows:

(14) $$R_{c}=\omega_{X}\alpha_{c}.$$

#### 2.4.2. Alternate Specific Cake Resistance (SCR) Submodels

Seven alternate SCR submodels were compared. In the first submodel, referred to as $\alpha_{c,1}$, the SCR was calculated with Equation (15) which combines the Carman–Kozeny equation for flow through a bed of spheres [55] with Darcy’s Law and the thickness of the cake layer as $\delta_{c}=\omega_{X}/\left(\rho_{X}\left(1-\varepsilon_{c}\right)\right)$.

(15) $$\alpha_{c,1}=\frac{k_{\mathrm{CK}}\;\left(1-\varepsilon_{c}\right)}{\rho_{X}\;d_{p}^{2}\;\varepsilon_{c}{}^{3}},$$

where $k_{\mathrm{CK}}$ is the proportionality Carman–Kozeny coefficient (which includes the shape factor), $\varepsilon_{c}$ is the cake layer porosity, $\rho_{X}$ is the density of particulate material deposited onto the membrane, and $d_{p}$ is the mean diameter of the deposited particles which was assumed equal to the mean diameter of the particles in the bulk liquid. The latter assumption neglects the selectivity towards the deposition of smaller particles, which has been previously described [56]. However, this assumption was regarded as sufficient in this first modelling approach to assess the necessity of incorporating floc size as a linking variable between biochemical-flocculation and filtration models. The effect of the smaller particles deposited onto the membrane was accounted for by incorporating colloidal material as a state variable.

The colloidal material entrapped in the cake layer decreases the cake layer porosity ($\varepsilon_{c}$) as follows [43]:

(16) $$\varepsilon_{c}=\varepsilon_{c0}-\left(1-\varepsilon_{c0}\right)\frac{\omega_{C}\;\rho_{X}}{\rho_{C}\;\omega_{X}},$$

where $\rho_{C}$ and $\omega_{C}$ are the density and mass per unit of area of colloidal material deposited on the membrane, respectively.

Several authors have reported the compression of the cake layer caused by TMP, which might cause deformation of soft sludge flocs and structural rearrangement of particles [57,58,59,60,61]. Therefore, we define the SCR submodels $\alpha_{c,1p}$ as the extended versions of $\alpha_{c,1}$ that includes cake compression. The SCR of the compressed cake layer at the operating pressure ($\alpha_{c,1p}$) is calculated using the SCR at zero pressure $\left(\alpha_{c,1}\right)$, the pressure drop over the cake ($∆P_{c}$), and the pressure needed to double the specific resistance ($P_{a}$), as follows:

(17) $$\alpha_{c,p}=\alpha_{c}\left(1+\frac{∆P_{c}}{P_{a}}\right).$$

By Darcy’s law $∆P_{c}=J\mu \omega_{X}\alpha_{c,p}$, and thus, combining Darcy’s law with Equation (17) the following equation was derived:

(18) $$\alpha_{c,1p}=\frac{\alpha_{c,1}}{\left(1-\frac{J\mu \omega_{X}\alpha_{c,1}}{P_{a}}\right)}.$$

The SCR submodel $\alpha_{c,2}$, was the model proposed by Wu et al. [43], presented in Equation (19), which does not include the dependency of $d_{p}$. Analogous to $\alpha_{c,1}$, $\alpha_{c,2}$ was extended into $\alpha_{c,2p}$, as shown in Table 1:

(19) $$\alpha_{c,2}=\frac{k_{c}\;{\left(1-\varepsilon_{c}\right)}^{2}}{\rho_{X}\;\varepsilon_{c}{}^{3}\left(1-\varepsilon_{c0}\right)},$$

where $k_{c}$ is a cake resistant coefficient and$\;\varepsilon_{c0}$ is the cake layer porosity without colloidal material.

Moreover, Cho et al. [44] developed an empirical equation to calculate SCR based on the concentration of extracellular polymeric substances, total suspended solids, and TMP. Several researchers have successfully applied this equation or slightly modified versions in aerobic MBRs [45,47,62,63]. Furthermore, Mannina, Suh and collaborators [62,63] modified Cho’s model to exclude the TMP dependency by using the TMP coefficient $P_{b}$. We included the following three SCR submodels based on Cho’s equation: $\alpha_{c,3}$, $\alpha_{c,3p}$, and $\alpha_{c,4p}$. The former, $\alpha_{c,3}$, corresponds to the equation presented by Mannina, Suh and collaborators, Equation (20); and $\alpha_{c,3p}$ is the compressible version of $\alpha_{c,3}$, presented in Table 1. Then, $\alpha_{c,4p}$ is an adapted version of Cho’s original equation, where the ratio EPS/TSS was substituted by $\omega_{C}$/$\omega_{X}$, presented in Equation (21). The model $\alpha_{c,4p}$ already includes cake compression because it is TMP dependent:

(20) $$\alpha_{c,3}=\frac{P_{b}}{\mu^{2}}\left(\zeta_{1}+\zeta_{2}{\left(1-\exp\left(-\zeta_{3}\frac{\omega_{C}}{\omega_{X}}\right)\right)}^{\zeta_{4}}\right),$$

(21) $$\alpha_{c,4p}=\frac{\mathrm{TMP}}{\mu^{2}}\left(\zeta_{1}+\zeta_{2}{\left(1-\exp\left(-\zeta_{3}\frac{\omega_{C}}{\omega_{X}}\right)\right)}^{\zeta_{4}}\right),$$

where $\zeta_{1}$, $\zeta_{2}$, $\zeta_{3}$, and $\zeta_{4}$ are empirical coefficients. In $\alpha_{c,4p}$, the SCR was calculated combining Equations (11), (13), (14), and (21), as follows:

(22) $$\alpha_{c,4p}=R_{m}{\left(\frac{\mu }{J\left(\zeta_{1}+\zeta_{2}{\left(1-\exp\left(-\zeta_{3}\frac{\omega_{C}}{\omega_{X}}\right)\right)}^{\zeta_{4}}\right)}-\omega_{X}\right)}^{-1}.$$

#### 2.4.3. Alternate Deposition Submodels

Two modelling approaches were compared to describe the cake layer formation by deposition of particulate material on the membrane surface. The first approach, further called “Deposition Submodel 1 (D1)”, was developed by Robles et al. [58] to describe the filtration process in a submerged AnMBR. The second approach, “Deposition Submodel 2 (D2)”, was developed by Li and Wang [64] and applied by different researchers in aerobic MBRs [43,45,61,62,63,65]. The deposition of colloidal material in the cake layer was based on the approach of Wu et al. [43]. Table 2 shows the stoichiometric coefficients and the kinetic expressions for both deposition submodels, and the extensions for colloidal material deposition.

Deposition Submodel 1 (D1) includes two processes related to particulate material. Process 1 is the attachment of particulate material onto the membrane promoted by the flow of permeate and as a function of the concentration of particulate material in the bulk liquid ($c_{X}$), Equation (23), where $J_{20}$ is the 20 °C normalised transmembrane flux. Process 2 is the detachment of particulate material promoted by the shear stress caused near the membrane surface by biogas sparging in the membrane tank, Equation (25). Robles et al. [58] also included the particulate material detachment during backflushing which was not included in our model since the pilot-scale AnMBR did not operate with backflushing.

Robles et al. [58] modelled Process 2 (detachment of particulate material) as a half-saturation switching function, Equation (25), where $q_{m,\mathrm{MS}}$ is the maximum membrane scouring velocity, $K_{S,c}$ is the half-saturation coefficient for cake mass during membrane scouring, $A_{m}$ is the membrane surface area, $u_{G}$ is the gas superficial velocity in the AnMBR membrane tank, and $H_{\mathrm{MT}}$ is the liquid level in membrane tank. Furthermore, based on experimental observation, they included a sigmoid inhibition function ($I_{\mathrm{MS}}$) to account for the impact of filtering at conditions above and below critical levels, Equation (31) in Table 3, where $K_{F}$ is an adjustable parameter representing the fouling rate when $J_{20}$ approaches zero; $\gamma_{0}$, $\gamma_{1}$, and $\gamma_{2}$ are parameters representing the influence of filtering capacity, biogas sparging, and particulate material on the fouling rate, respectively.

Different equations to calculate $I_{\mathrm{MS}}$ were developed, summarised in Table 3, to be included in Deposition Submodel D1. Depostion Submodel D1a is the original model by Robles et al. [58], D1b is an extension to account for the influence of colloidal material with the parameter $\gamma_{3}$. D1c is a simplified submodel that eliminates the impact of filtering at conditions above and below critical levels (i.e., $I_{\mathrm{MS}}=1$).

Robles’ model [58] was extended to incorporate the deposition of colloidal material on the membrane surface. Analogous to the attachment of particulate material, the attachment of colloidal material to the membrane (Process 3) was promoted by the permeate flow and the concentration of colloidal material in the bulk liquid ($c_{C}$), Equation (27). The detachment of colloidal material from the membrane (Process 4), Equation (29), was caused by the detachment of the particulate material weighed by the ratio of colloidal to total material deposited.

Deposition Submodel 2 (D2) describes the attachment of particulate material (Process 1) based on two competing forces, namely attraction drag from the permeate flow and lifting force caused by the shear stress near the membrane surface. The kinetic expression of Process 1 is in Equation (24), where $C_{d}$ is the drag coefficient, G is the apparent shear rate, and J is the transmembrane flux (at the operating temperature). The detachment of particulate material, Process 2, was calculated with Equation (26), where γ is the compression coefficient, $V_{F}$ is the volume of permeate produced within the filtration time $t_{F}$ with $V_{F}=Jt_{F}$, and $\beta_{\mathrm{ST}}$ is a lumped parameter with $\beta_{\mathrm{ST}}=\beta \left(1-K_{\mathrm{ST}}\right)$ where β is the erosion rate coefficient of the cake layer and $K_{\mathrm{ST}}$ is the stickiness coefficient.

The apparent shear rate, G, was calculated based on the $u_{G}$, and the density ($\rho_{L}$) and dynamic viscosity ($\mu_{L}$) of the mixed liquor, as follows:

(34) $$G=\sqrt{\frac{\rho_{L}\;g\;u_{G}}{\mu_{L}}}.$$

We assumed that $\rho_{L}$ is equal to the density of water ($\rho_{W}$) at the operational temperature T (K), The parameters of the quadratic function in Equation (35) were optimised to fit the $\rho_{W}$ versus T data [66], with a coefficient of determination (R^2^) of 0.9997:

(35) $$\rho_{W}=-0.0033\;T^{2}-0.1048\;T+1001.5.$$

The viscosity $\mu_{L}$ was a function of the concentration of solids in the bulk liquid (TSS) and the viscosity of water at T, as follows [67]:

(36) $$\mu_{L}=a\;\mu_{W}\;e^{b\;\mathrm{TSS}},$$

where a and b are parameters, with a = 1.05 and b = 0.08.

In Deposition Submodel 2, the attachment and detachment of colloidal material to the membrane was modelled following Wu et al. [43]. The attachment (Process 3) was calculated with Equation (33), where $f_{C,c}$ is the fraction of colloidal material entrapped in the cake layer. The detachment (Process 4) was caused by detachment of the cake layer, calculated with Equation (35).

### 2.5. AnMBR Filtration: Alternate Empirical FR Models

The empirical FR models are algebraic equations to calculate FR directly from the operational variables and mixed liquor properties, summarised in Table 4. The first FR model, FR1, is the one proposed by Robles et al. [58] for $I_{\mathrm{MS}}$ calculation and extended for colloidal material, presented in Equation (32). This model was further modified into FR2 by eliminating the effect of $c_{X}$, because the concentration of particulate material is a poor indicator of biomass fouling propensity by itself [28].

Based on the gas-step experiments in the pilot-scale AnMBR described in Section S1, the FR was proportional to $u_{G}^{-\gamma_{G}}$, where $\gamma_{G}$ is a parameter, this was incorporated in FR3. The conversion factor $f_{\mathrm{conv}}$ was introduced to achieve similar FR values as the model FR2 as follows: $f_{\mathrm{conv}\;}$ = ${\bar{u_{G}}}^{\gamma_{G}}$, where $\bar{u_{G}}$ is the mean gas velocity in the pilot-scale AnMBR, $\bar{u_{G}}$ = 0.003 m s^−1^.

As discussed in Odriozola et al. [13], it is not clear if the floc size influences membrane fouling. Therefore, we compared empirical FR models including and excluding mean particle size, $d_{p}$, as an input variable. The empirical models FR1, FR2, and FR3 were extended into FR4, FR5, and FR6, respectively, that included $d_{p}$.

### 2.6. AnDFCm Filtration: Alternate Models

The $∆R_{20}$, which is inversely related with sludge filterability, is the additional resistance obtained when a specific permeate volume ($V_{F}$) of 20 L per m^2^ of membrane area is produced in the AnDFCm installation. The $∆R_{20}$ was measured at 60 L m^−2^ h^−1^ (1.67 × 10^−5^ m^3^ m^−2^ s^−1^) transmembrane flux ($J_{\mathrm{AnDFCm}})$ and 1.5 m s^−1^ crossflow velocity ($u_{L,\mathrm{AnDFCm}}$). At 60 L m^−2^ h^−1^ flux, the final filtration time to obtain 20 L m^−2^ of permeate is 1200 s. Therefore, the AnDFCm filtration models predicted $∆R_{20}$ as $R_{c}$ after 1200 s of continuous filtration under the operational conditions of the AnDFCm starting with a clean membrane, meaning the initial conditions are $w_{C}$ = 0 and $w_{X}$ = 0, thereby, initial $R_{c}$ = 0.

The cake layer resistance, $R_{c}$, was calculated with Equation (14), and combining the alternate SCR submodels described in Section 2.4.2 and the alternate deposition submodels in Section 2.4.3, with the following modifications:

- The gas superficial velocity in the AnMBR membrane tank ($u_{G}$) was replaced by the crossflow velocity in the AnDFCm membrane tube ($u_{L,\mathrm{AnDFCm}}$ = 1.5 m s^−1^);
- The transmembrane flux was J = $J_{\mathrm{AnDFCm}}$ (1.67 × 10^−5^ m^3^ m^−2^ s^−1^) and $J_{20}$ = $J_{\mathrm{AnDFCm}}\;\mu /\mu_{20}$;
- There were no relaxation cycles (continuous filtration);
- The parameters of the mixed liquor viscosity model, a and b in Equation (36), were estimated based on the viscosity measurement performed in the AnDFCm installation with sludge samples with different TSS, presented in Section S2;
- Deposition Submodels D1a and D1b were not used because they were equal to D1c for the AnDFCm installations; the superficial velocity in the AnDFCm installation ($u_{L,\mathrm{AnDFCm}}$ = 1.5 m s^−1^) was three orders of magnitude higher than in the AnMBR (0.5 × 10^−3^ < $u_{G}$ < 5.7 × 10^−3^ m s^−1^), and thus $I_{\mathrm{MS}}≅1$ in Equations (31) and (32).

The AnDFCm installation operates in continuous filtration mode and at fixed transmembrane flux and crossflow velocity. Thus, in addition to D2 and D1c, we propose a simplified deposition submodel, referred to as “Deposition Submodel 3” (D3), as follows:

(42) $$\omega_{X}=f_{X,c}\;V_{F}\;c_{X},$$

(43) $$\omega_{C}=f_{C,c}\;V_{F}\;c_{C},$$

where $f_{X,c}$ and $f_{C,c}$ are the fractions of particulate and colloidal materials deposited onto the membrane, respectively. These fractions represent the balance between the different forces acting over the particles. When $f_{X,c}$ and $f_{C,c}$ both equal one, all the material is deposited in the membrane, analogous to dead-end filtration. Deposition Submodel 3 consisted of algebraic equations instead of ordinary differential equations (ODE), which simplified the resolution and computational cost considerably. Deposition Submodels D1c, D2, and D3 were coupled with the 7 alternate SCR submodels in Table 1, obtaining 21 alternate AnDFCm models to compare.

### 2.7. Model Implementation and Parameter Values

The ODE of the biochemical-flocculation model was solved with the built-in ODE solver ode15s in Matlab^®^ R2019b, using a timestep of 0.01 d (864 s). The ODE of the deposition submodels were solved using ode45 in Matlab^®^ R2019b to obtain $\omega_{C}$ and $\omega_{X}$ as a function of time. The timestep was set sufficiently low, 10 s, to capture the operational stages (filtration and relaxation) in the pilot-scale AnMBR and avoid numerical problems. Subsequently, the SCR was calculated applying the equations in Table 1.

Most parameter values were taken from the literature [37,43,45,52,58,63,64,68,69,70,71,72,73,74,75,76,77,78,79,80,81,82] or were estimated based on experimental data and different assumptions, as described in Section S4.

### 2.8. Model Calibration and Validation

The biochemical-flocculation model, the 34 alternate AnMBR filtration models, and the 21 alternate AnDFCm filtration models were calibrated separately. The calibration procedure, detailed in Section S5, consisted of the following steps: (1) the subset containing only influential parameters ($\theta_{I}$) was selected using global sensitivity analysis (GSA), (2) identifiability analysis from $\theta_{I}$ was used to select a new subset $\theta_{II}\;$that can be reliably estimated from the given experimental data, (3) the parameters in $\theta_{II}$ were estimated, (4) $\theta_{III}$ was defined with the parameters contained in $\theta_{I}$ and not in $\theta_{II}$, and (5) the parameters in $\theta_{III}$ were estimated. The sample size for GSA was selected by convergence analysis [83]. The quality of the estimators $\hat{\theta }$ was evaluated based on the relative error ($\sigma_{\theta }/\hat{\theta }$) as follows: below 0.1 good, above 0.5 poor [84], and between 0.1 and 0.5 moderate. During model validation, the predictive capacity of the calibrated models was quantified with statistical indicators, presented in Section S6, and was assessed visually with plots comparing experimental and simulated values.

The parameters related to the flocculation kinetic process in the biochemical-flocculation model, namely the subset $\theta \;=\;\left\{q_{m,\mathrm{ads}},K_{L,\mathrm{ads}},k_{\mathrm{ads}},Y_{\mathrm{fe},C},k_{\mathrm{floc},\mathrm{fe}}\right\}$, were optimised with the submicron COD (csCOD) and particle size measured in the dosage-step experiments described in Section 2.1. The experimental particle size was incorporated in the model as the geometric mean diameter $d_{p}$ [85]. Subsequently, the remaining adjustable parameters in the biochemical-flocculation model were optimised using the long-term measurements of colloidal COD (cCOD), TSS, and $d_{p}$ in the pilot-scale AnMBR. The mean blackwater characteristics values were used as inputs for model implementation. The same dataset was used for calibration and validation of the long-term prediction, and thus, the biochemical-flocculation model requires further validation with an independent dataset from an independent operational period of the pilot or from another AnMBR.

The AnMBR filtration model was calibrated using eight datasets from the operation of the pilot-scale AnMBR, whereby each dataset covered an 8-hour period. These eight calibration datasets were selected to capture changes in the following operational conditions: gas sparging, mean particle size, and concentration of colloidal and particulate material. The model was validated by predicting the entire operational period of the pilot. However, the validation should be improved by applying the model to an independent operational period of the pilot or to another AnMBR, but this data was not available during our research.

The AnDFCm model was calibrated using in situ $∆R_{20}$ measurement in the pilot AnMBR immediately after FE dosing and ex situ $∆R_{20}$ measurement during the dosage-step tests performed with grab samples from the pilot AnMBR. The model was validated using long-term in situ measurements of $∆R_{20}$ measured in the pilot-scale AnMBR.

### 2.9. Control Tools for Flux Enhancer Dosage

We proposed and compared different feedforward and feedback control tools to manipulate the FE mass flow rate fed to the reactor (${\dot{m}}_{\mathrm{fe}}$), as summarised in Table 5. The control tool FB_$∆R_{20}$_10 is a feedback loop to control $∆R_{20}$ to a target setpoint ($∆R_{20,\mathrm{sp}}$); $∆R_{20,\mathrm{sp}}$ of 10 × 10^12^ m^−1^ is an intermediate value between the pilot-scale AnMBR operation before and immediately after FE dosing. The control tool FB_$∆R_{20}$_8–12 is similar to the latter, but $∆R_{20}$ is maintained inside a target range instead of to a specific value. FB_$∆R_{20}$_8–12 starts dosing FE (on) when $∆R_{20}$ is above 12 × 10^12^ m^−1^ and stops (off) when $∆R_{20}$ is below 8 × 10^12^ m^−1^, thereby, causing periodic FE pulses instead of a continuous dosage (as in FB_$∆R_{20}$_10).

The feedforward control tool FF_$Q_{\mathrm{WS}}$ is analogous to the mostly applied FE dosing strategy reported in the literature, that is, an initial FE pulse dosage that is followed by periodic additions to compensate for the loss of FE with sludge withdrawal and biodegradation [13], with the objective to maintain a certain concentration of FE inside the reactor. The FE is not biodegradable in the proposed model, therefore, FF_$Q_{\mathrm{WS}}$ does not compensate for FE loss by biodegradation. Furthermore, an alternative dosing strategy used in literature is a step of FE on the influent [86,87], which was implemented in the control tool FF_$Q_{\mathrm{Inf}}$.

The simulation without FE dosing, No_FE, was included to assess the improvement achieved when FE is added to the reactor by the control tools. Moreover, the feedback $c_{\mathrm{fe}}$ control tool FB_$c_{\mathrm{fe}}$ was included to compare with FF_$Q_{\mathrm{WS}}$ and FF_$Q_{\mathrm{Inf}}$, whose controlled variable is also $c_{\mathrm{fe}}$. Nevertheless, to apply FB_$c_{\mathrm{fe}}$ in practice, a method to measure $c_{\mathrm{fe}}$ should be developed.

The feedback control tools were manually tuned to achieve a slow response (low ${\dot{m}}_{\mathrm{fe}}$) to avoid overdosing. The FE concentration setpoint ($c_{\mathrm{fe},\mathrm{sp}}$) was 8.7 × 10^−3^ kgCOD m^−3^, which is equal to the concentration needed to achieve a $∆R_{20}$ of 10 × 10^12^ m^−1^ at the beginning of the simulated operational period. For FF_$Q_{\mathrm{Inf}}$, the ratio of FE to influent flow ($Y_{\mathrm{fe},\mathrm{Inf}}$) was 7.23 × 10^−4^ kgCOD m^−3^, calculated as the ratio between the cumulative masses of FE and influent fed to the reactor during the first 100-day period simulated with FB_$∆R_{20}$_10.

The control tools were implemented and tested in Simulink, Matlab^®^ 2019b, by using the integrated model composed by the calibrated biochemical-flocculation model, and the best alternates of the calibrated AnMBR and AnDFCm filtration models. The implementation included a feedback TSS controller which manipulated $Q_{\mathrm{WS}}$ to sustain the TSS at a fixed setpoint (TSS_sp_). A constant mixed liquor volume was assumed, whereby, $Q_{\mathrm{Inf}}$ was calculated with the mass balance in Equation (S.4) with $∆V_{L}$ = 0; and $Q_{\mathrm{fe}}$ = ${\dot{m}}_{\mathrm{fe}}/c_{\mathrm{fe},\mathrm{stock}}$, where $c_{\mathrm{fe},\mathrm{stock}}$ is the concentration of the stock solution fed to the reactor (30 kgCOD m^−3^).

The model inputs were: T, pH, $J_{20}$, $u_{G}$, and concentration of ammonium (NH4_BW_) and alkalinity (Alk_BW_) in the blackwater, and were assumed constant and equal to the mean values in the pilot-scale AnMBR (Section S1). The total and submicron blackwater COD fluctuated inside the range of the pilot; the input was generated with the “uniform random number” block from Simulink as shown in Section S7.

The fraction of components i in the waste sludge, $f_{i,\mathrm{WS}}$, were estimated based on the sludge withdrawal made on Day 123, where 31% of the mixed liquor volume was removed causing a 62% decrease in TSS and 7% decrease in csCOD. Therefore, $f_{i,\mathrm{WS}}$ was assumed as 2.0 for all particulate material ($f_{X,\mathrm{WS}}$), and 0.22 for colloidal material ($f_{C,\mathrm{WS}}$).

Furthermore, the robustness of the control tools was tested by applying step disturbances in ${\mathrm{TSS}}_{\mathrm{sp}}$ and $f_{C,\mathrm{WS}}$. The ${\mathrm{TSS}}_{\mathrm{sp}}$ were 9.6, 5.5, and 16.0 kg m^−3^ corresponding to the mean, minimum, and maximum TSS in the pilot; the steps on ${\mathrm{TSS}}_{\mathrm{sp}}$ were performed on Days 100 and 200. The initial $f_{C,\mathrm{WS}}$ was 0.22 and increased to 1 on Day 300, owing to a better mix before wastage.

## 3. Results and Discussion

### 3.1. Biochemical-Flocculation Model

**Figure 2:**
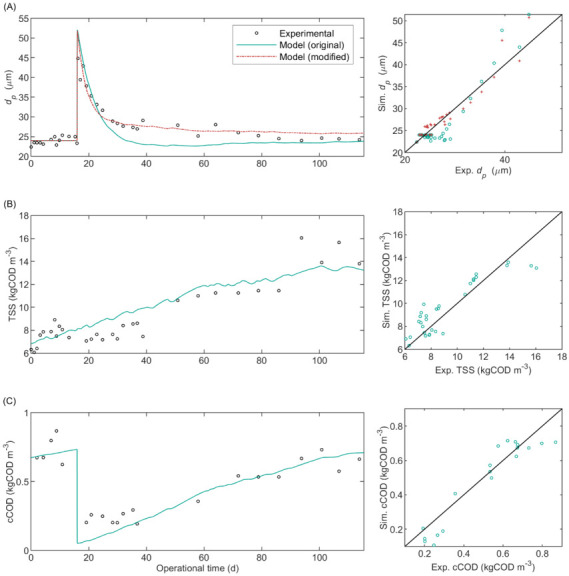
Long-term biochemical-flocculation model calibration. Sludge characteristics during operational period of pilot AnMBR plant dosed with flux enhancer on day 16: (**A**) mean particle diameter; (**B**) total suspended solids; (**C**) colloidal COD.

The calibration results are detailed in Section S8. Figure 2 shows the experimental data and model predictions with the estimated parameters. The model predicted a sharp cCOD decrease and $d_{p}$ increase caused by FE dosing on Day 16. Then, cCOD slowly increased over time due to the accumulation of colloidal inerts coming from the influent, decayed biomass, and deflocculation. The latter was caused by the loss of unbonded FE ($S_{\mathrm{fe}}$) with the permeate flow that lowered the equilibrium concentration $X_{\mathrm{fe},e}$ causing desorption of FE from the particulate material (i.e., $\rho_{23}$ < 0), displayed in Figure S9B, and concomitantly deflocculation (i.e., $\rho_{24}$ < 0).

The predicted $d_{p}$ decrease, after the sharp increase on Day 16, was overpronounced as compared with the experimental observations. To improve the predictive capacity, we substitute Equation (10) with the modified Equation (44), where the stable mean particle size was proportional to the ratio between $c_{\mathrm{fe}}$ and the total concentration of suspended material ($c_{X}$ + $c_{C}$) inside the reactor, with a proportionality parameter $Y_{\mathrm{floc},\mathrm{fe}}$:

(44) $$\frac{dd_{p}}{dt}=\frac{1}{t_{\mathrm{conv}}}\left(k_{\mathrm{floc},\mathrm{fe}}\frac{dX_{\mathrm{fe}}}{dt}+\left(Y_{\mathrm{floc},\mathrm{fe}}\left(\frac{c_{\mathrm{fe}}}{c_{X}+c_{C}}\right)d_{p,\mathrm{St}}-d_{p}\right)k_{\mathrm{floc}}\right).$$

The parameters $k_{\mathrm{floc}}$ and $Y_{\mathrm{floc},\mathrm{fe}}$ were optimised to fit the experimental floc size; the optimal values for $k_{\mathrm{floc}}$ and $Y_{\mathrm{floc},\mathrm{fe}}$ were 0.34 d^−1^ and 46.9 kg kgCOD^−1^ with $\sigma_{\theta }/\hat{\theta }$ of 0.28 and 0.35, respectively. The modified model presented an improved accuracy to predict $d_{p}$ as compared with the original model, evidenced by the lower mean absolute percentage error (MAPE) in Table S12, that is, 4.2 and 7.1 for the modified and original models, respectively. Accordingly, Figure 2A shows that the modified model in Equation (44) fitted better to the experimental values than the original model in Equation (10).

The model predicted a continuous TSS increase, shown in Figure 2A, caused by the accumulation of inert material (Figure S9A) coming from the influent and decaying biomass because the reactor was operated without sludge wastage. Because the model considered constant influent composition, the fluctuations in TSS were caused by fluctuations of the influent flow rate and of the temperature and pH of the mixed liquor (which affect the conversion rates). However, in the pilot-scale AnMBR, the fluctuations in the solids content were affected by variations in the blackwater composition, which was highly variable throughout the operational period [13]. Therefore, the predicted TSS deviated from the experimental values, likely due to the lack of an exact characterisation of the influent. Additionally, the applied physicochemical characterization was not sufficient to detect all the fluctuations in the exact blackwater composition. Therefore, a more comprehensive and frequent blackwater characterization using flow-proportional sampling should be done to predict the exact TSS dynamics.

### 3.2. AnMBR Filtration Model

**Figure 3:**
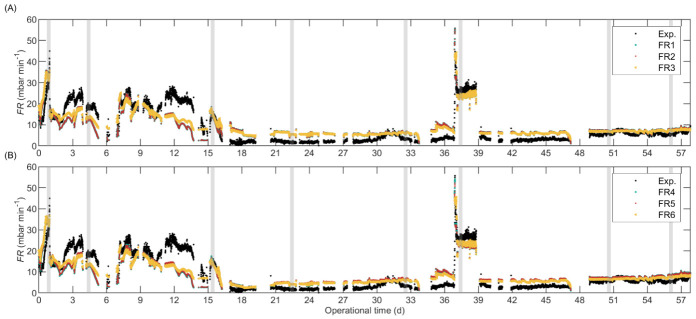
Validation of the alternate AnMBR empirical fouling rate (FR) models which (**A**) exclude and (**B**) include floc size as input variable. The grey vertical areas represent the representative dataset (iD1 to iD8 from left to right) used for model calibration. Imposed limits between 0 and 60 in the *y*-axis.

**Figure 4:**
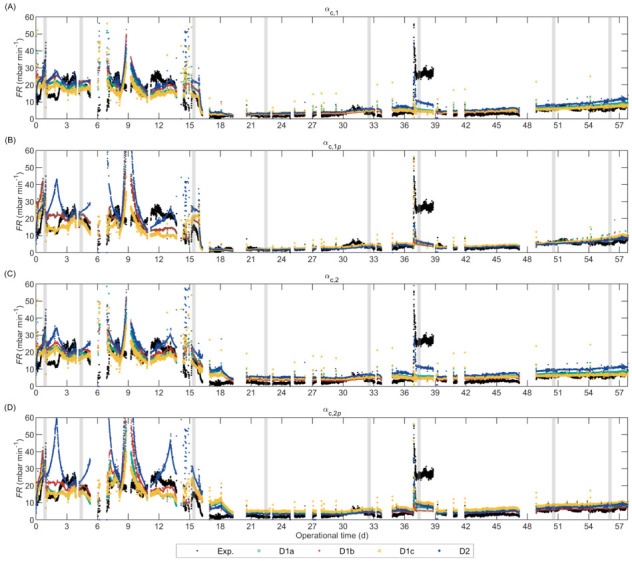
Validation of the alternate FR_RIS AnMBR filtration models combining the different deposition submodels (D1a, D1b, D1c, and D2) with the Carman–Kozeny based specific cake resistance submodels: (**A**) $\alpha_{c,1}$; (**B**) $\alpha_{c,1p}$; (**C**) $\alpha_{c,2}$; (**D**) $\alpha_{c,2p}$. The grey-vertical areas represent the representative dataset (iD1 to iD8 from left to right) used for model calibration. Imposed limits between 0 and 60 in the *y*-axis.

The calibration results are detailed in Section S9. The predictive capacity of the calibrated models was assessed by analysing the predictions during the entire operation of the pilot-scale AnMBR, shown in Figure 3 for the empirical FR models and in Figure 4 for the FR_RIS models that included Carman–Kozeny based SCR submodels. The *y*-axis was limited between 0 and 60 for better visualization and discussion; Section S9 Figures S15–S23 display the individual plots for each model without imposed limits. The models that included the SCR submodels $\alpha_{c,3}$, $\alpha_{c,3p}$, or $\alpha_{c,4}$ were not further analysed because they could not be satisfactorily calibrated with the procedure described in Section 2.8, and thus, were unable to predict the representative data used for calibration. The results are shown in Section S9.

In Figure 3, the empirical models FR1 and FR2 had identical predictions during the entire operation of the pilot-scale AnMBR; the only difference between these models was that FR1 included $c_{X}$ and FR2 did not. Apparently, $c_{X}$ had no influence on FR prediction in our case and could, therefore, be removed from the model. The same conclusion was derived after comparing FR4 and FR5.

In general terms, all the FR_RIS models in Figure 4 and empirical FR models in Figure 3 predicted satisfactorily the effect of $c_{C}$ on the fouling rate. During the period without FE (0–16 d) the experimental and predicted FR values were considerably higher than the FR after FE dosing (after Day 16). Nevertheless, during the initial period (0–16 d), the empirical FR models considerably underpredicted the FR at high $u_{G}$ (periods 4.5–7 and 12–15 days), which is further explained below. The deposition submodels based on Robles et al. [58], that is, D1a, D1b, and D1c, presented similar behaviour, and D1b was slightly more sensitive to $c_{C}$ than D1a and D1c because in D1b $c_{C}$ affects the deposition of particulate material through $I_{\mathrm{MS}}$.

The lack of online gas flow measurements is a limitation for model calibration and validation, especially for the empirical FR models that are highly sensitive to $u_{G}$. For example, the biggest deviation between the experimental data and the predicted values was between Days 12 and 15 where the reactor operated at a low liquid level in the membrane tank ($H_{\mathrm{MT}}$) causing a high simulated $u_{G}$ [13]. However, the simulated $u_{G}$ during this period could not be confirmed with experimental data, therefore, we could not ensure that the input variable $u_{G}$ was correct or if the actual values were lower, and the model could have predicted the FR accurately. Similarly, in the period 34.8–36.8 d the simulated $u_{G}$ was 8 to 36% lower than the experimental (manually recorded) $u_{G}$ which caused the overpredicted FR values. Therefore, to improve model calibration and validation the biogas should be monitored online to provide a reliable input variable.

Similarly, the use of grab samples for determining the input variables for sludge characterization limited model calibration and validation. This was particularly true for the fluctuating sludge characteristics that were highly influential in the model, such as $c_{C}$, which was calculated as the difference between the measured csCOD and permeate COD (pCOD). The pCOD was relatively stable during the reactor operation, however, csCOD fluctuated considerably (particularly before FE dosing on day 16) and had only few datapoints. For model implementation, we linearly interpolated between measurements resulting in $c_{C}$ with sharp fluctuations and peaks that caused fluctuations in the simulated FR, but the true values of $c_{C}$ between grab samples could not be confirmed, hampering proper model validation.

The FR_RIS models overpredicted the fouling rate at high $c_{C}$ (>0.5 kgCOD m^−3^), and the overprediction was higher for D2. From the Robles et al. [58] based FR_RIS models, D1c $\alpha_{c,1p}$ and D1c $\alpha_{c,1}$, had the lowest fouling rate overpredictions, and D1c $\alpha_{c,1p}$ was slightly better than D1c $\alpha_{c,1}$. The models that combined the Depostiion Submodel D2 with a Carman–Kozeny-based SCR submodel (i.e., $\alpha_{c,1}$, $\alpha_{c,1p}$, $\alpha_{c,2}$, and $\alpha_{c,2p}$) presented instabilities or pronounced peaks at high $c_{C}$ (shown in Figure S1D), which was attributed to the considerably low estimated $\varepsilon_{c0}$ of 0.12–0.17 (Table S15). When colloidal material accumulated in the cake, the porosity ($\varepsilon_{c}$) was reduced below $\varepsilon_{c0}$, resulting in values close to zero causing an overpronounced increase in SCR because of the term: $\alpha_{c}\propto \varepsilon_{c}{}^{-3}$ (Table 1). The low $\varepsilon_{c0}$ value in D2 was estimated because this deposition submodel predicted approximately 200 times less material deposition onto the membrane surface than the deposition submodels based on Robles et al. [58], and thus, the SCR increased (by decreasing the porosity) to reach similar $R_{c}$ values. To elucidate which modelling approach was more accurate, the amount of particulate and colloidal material deposited should be measured, which was unfortunately not possible in our research.

The models that included floc size as input variable (Figure 3B and Figure 4A,B) improved the FR prediction at large floc size (i.e., operational period 17–30 days) as compared with the models that did not include floc size (Figure 3A and Figure 4C,D). These results suggest that floc size had a direct impact on FR and should be included as a state variable in a model that predicts the effect of FE on FR.

During days 37–39, the reactor was operated at considerably low $u_{G}$ causing a sharp fouling rate increase despite the low $c_{C}$. The empirical FR models predicted this behaviour satisfactorily and only slightly underestimated the fouling rate. Additionally, the empirical FR models adequately predicted the fouling rate increase caused by the $u_{G}$ decrease during Day 1 in which $c_{C}$ was high. Contrarily, all FR_RIS models predicted only a slight or no increase in fouling rate during Days 37–39, thus, substantially underestimating the fouling rate. Nevertheless, some FR_RIS models (namely the ones with $\alpha_{c,1p}$, $\alpha_{c,2p}$, or D2) could predict the fouling rate increase during Day 1. The Deposition Submodel D2, that considered drag and lift forces, was slightly more sensitive to $u_{G}$ than the submodels that considered only drag forces (D1a, D1b, and D1c) because, in D2, $u_{G}$ affects the attachment and detachment of particulate material (through G), whereas, in the other submodels, only the detachment is affected by $u_{G}$. In summary, at high $c_{C}$, the effect of low $u_{G}$ on fouling rate was satisfactorily predicted by all the empirical FR models and by the FR_RIS models that included either the SCR submodels with cake compression ($\alpha_{c,1p}$ and $\alpha_{c,2p}$) or Deposition Submodel D2; whereas at low $c_{C}$, only the empirical FR models could predict the effect of low $u_{G}$ on fouling rate.

The reactor was not intentionally operated at high $u_{G}$; however, during some periods, $H_{MT}$ decreased due to influent shortage which increased the simulated $u_{G}$ [13]. However, the experimental $u_{G}$ was measured a few times during those periods: the experimental $u_{G}$ on Day 14 was 8% lower than the calculated value; on Day 7 the calculated and experimental values were equal. Thus, we analysed the prediction at high $u_{G}$ on Day 7. All the empirical FR models and the FR_RIS models that combined Deposition Submodels D1a, D1b, or D1c with SCR submodels with cake compression ($\alpha_{c,1p}$ or $\alpha_{c,2p}$) satisfactorily predicted the fouling rate at high $u_{G}$, whereas the models that included SCR submodels without cake compression ($\alpha_{c,1}$ or $\alpha_{c,2}$) or the Deposition Submodel D2, overpredicted the fouling rate. Particularly, for D2, the fouling rate was substantially high, because the $c_{C}$ was elevated and caused instabilities in the model, as explained above.

### 3.3. AnDFCm Filtration Model

**Figure 5:**
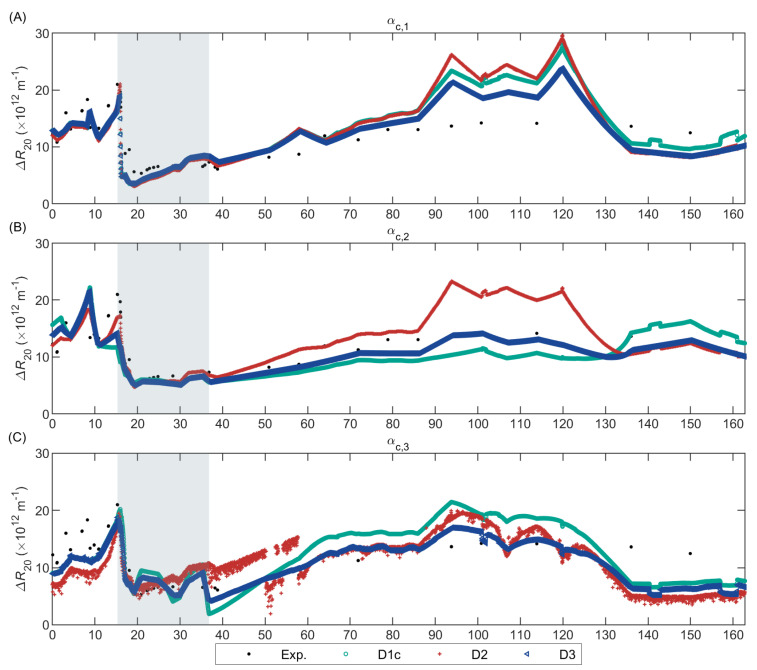
Validation of the alternate AnDFCm filtration models that combine the different deposition submodels (D1c, D2, and D3) with the non-compressible specific cake resistance submodels: (**A**) $\alpha_{c,1}$; (**B**) $\alpha_{c,2}$; (**C**) $\alpha_{c,3}$. The grey area represents the in situ data used for model calibration.

Figure 5 compares the experimental and predicted long-term $∆R_{20}$ of the pilot-scale AnMBR sludge for the alternate models without cake compression; Figure S27, in Section S11, shows the predictions of the alternate models with cake compression.

Figure S27 suggested that the model with D3 could predict the experimental $∆R_{20}$ when combined with cake compression SCR submodels. However, as explained in Section S11, the models combining D3 with $\alpha_{c,1p}$, $\alpha_{c,2p}$, or $\alpha_{c,3p}$, in fact, did not describe a compressible cake. Therefore, all the models that included cake compression were unable to predict the experimental $∆R_{20}$. Accordingly, the shape of the filtration curve obtained when filtering different anaerobic sludge samples in the AnDFCm installation suggested that the cake layer formed was mostly non-compressible. As further discussed in Section S13, the cake compressibility analysis was done based on the possible hypothetical filtration curves previously defined [50,88,89].

The alternate AnDFCm filtration models without cake compression, shown in Figure 5, satisfactorily predicted the filterability improvement (i.e., $∆R_{20}$ decrease) caused by dosing FE on Day 16. During the period without FE (0–16 d), the experimental and predicted $∆R_{20}$ values were considerably higher than the $∆R_{20}$ after FE dosing (after Day 16) for the models with $\alpha_{c,1}$ or $\alpha_{c,2}$. However, for the models with $\alpha_{c,3}$, the difference between these periods was less clear because the models predicted relatively high $∆R_{20}$ in the period 20–35 d, which was caused by small fluctuations in $c_{C}$ and $c_{X}$.

Section S10 shows the sensitivity of the models to $c_{X}$ and $c_{C}$ inside the operational range of the pilot. Sludge was withdrawn from the pilot on Day 123 causing a drop in $c_{X}$ from 14 to 5.5 kg m^−3^, whereas $c_{C}$ and $∆R_{20}$ were almost unaltered. Figure 5 shows that D3 $\alpha_{c,2}$ was the only model that accurately predicted this behaviour because it had only moderate sensitivity to $c_{X}$, as illustrated in Section S10. The models that included $\alpha_{c,1}$, $\alpha_{c,3}$, or D2 overpredicted the $∆R_{20}$ drop after sludge withdrawal because models with $\alpha_{c,1}$ or D2 were too sensitive to $c_{X}$ and, with $\alpha_{c,3}$ were too sensitive to $c_{C}/c_{X}$. The high sensitivity of $\alpha_{c,1}$ and D2 to $c_{X}$ also caused the overprediction when $c_{X}$ was high (85–125 d). Similarly, D1c $\alpha_{c,2}$ had an elevated sensitivity to $c_{X}$, but with an opposite effect on $∆R_{20}$, that is, a higher $c_{X}$ caused a lower $∆R_{20}$, consequently, D1c $\alpha_{c,2}$ overpredicted $∆R_{20}$ at low $c_{X}$ (after sludge withdrawal) and underpredicted $∆R_{20}$ at high $c_{X}$ (85–125 d).

Opposite to the AnMBR filtration models, the incorporation of $d_{p}$ as input variable in the AnDFCm filtration models worsened the $∆R_{20}$ prediction. During the operational period at large floc size (17–25 days), the models with $\alpha_{c,2}$ (without $d_{p}$) accurately predicted the experimental $∆R_{20}$, whereas the models with $\alpha_{c,1}$ (with $d_{p}$) underpredicted $∆R_{20}$. Additionally, the models with $\alpha_{c,1}$ predicted peaks in $∆R_{20}$ around Days 60, 94, and 120, caused by small $d_{p}$ reductions; these $∆R_{20}$ peaks were not observed experimentally. These results suggested that floc size might not have a direct impact on sludge filterability and could be excluded as state variable in the AnDFCm filtration models for $∆R_{20}$ prediction. The negligible effect of floc size on sludge filterability might be caused by the absence of relaxation cycles in the AnDFCm, as previously proposed in Section 4.1.2 in Odriozola et al. [13].

### 3.4. Model Limitation, Applicability, and Further Development

The calibrated biochemical-flocculation model satisfactorily predicted the dynamics of $d_{p}$ and cCOD in the pilot-scale AnMBR dosed with FE. Nevertheless, a more frequent and comprehensive influent characterization is needed to improve model calibration and validation to accurately predict the fluctuations in TSS, $c_{X}$, and $c_{C}$. Additionally, the same dataset was used for calibration and validation, and thus, the model requires further validation with an independent dataset from an independent operational period of the pilot or from another AnMBR.

The biochemical-flocculation model included only inert colloidal material, the model could be further extended to incorporate biodegradable colloidal components consisting of proteins, carbohydrates, lipids, humic substances, etc. Furthermore, particle size prediction could be improved, for example, by incorporating a population balance model in the biochemical-flocculation model to predict the particle size distribution [48]. This would increase the complexity of the model by increasing the amount of state variables and parameters, but it might also increase the accuracy of the model.

From the alternate AnMBR filtration models, the FR_RIS that included Carman–Kozeny based SCR submodels and all the proposed empirical FR models satisfactorily predicted the effect of $c_{C}$ on fouling rate. Nevertheless, the empirical FR models might have underpredicted and the FR_RIS overpredicted FR at high $c_{C}$. Furthermore, all the empirical FR models and none of the FR_RIS models predicted the effect of the low $u_{G}$ on the fouling rate when the reactor was operated at low $c_{C}$. Therefore, we selected one empirical and one FR_RIS model for the simulation environment to cover a predicted fouling rate range. Nevertheless, the calibration of the alternate AnMBR filtration models should be further improved by online gas monitoring and applying more intensive monitoring of grab samples, particularly for csCOD which fluctuated and highly affected the model. Additionally, the validation should be improved by applying the alternate models to an independent dataset.

We selected the empirical FR model FR6 because it included $d_{p}$ as input variable, which improved the prediction, and better predicted FR at high $u_{G}$ as compared with FR4 and FR5. Additionally, FR6 was the alternate empirical FR model with the highest accuracy, as evidenced by the lowest MAPE (Table S18). From the FR_RIS models, D1c $\alpha_{c,1p}$ and D1c $\alpha_{c,1}$ had the lowest FR overpredictions at high $c_{C}$. In Section S10, D1c $\alpha_{c,1p}$ was more sensitive to $u_{G}$ and less sensitive to $c_{X}$ than D1c $\alpha_{c,1}$, as experimentally observed; therefore, we selected model D1c $\alpha_{c,1p}$ for the simulation environment. The accuracy of D1c $\alpha_{c,1p}$ was one of the lowest, together with D1a $\alpha_{c,1p}$ and D1b $\alpha_{c,1p}$, as shown by the lowest MAPE values in Table S18.

From the AnDFCm filtration models, the best alternate model to predict sludge filterability was D3 $\alpha_{c,2}$ because it had limited sensitivity to $c_{X}$ as experimentally observed, and it satisfactorily predicted the experimental $∆R_{20}$, including the $∆R_{20}$ decrease after FE dosing and the small change in $∆R_{20}$ value after sludge withdrawal. Together with D2 $\alpha_{c,2}$, D3 $\alpha_{c,2}$ had the lowest MAPE (Table S23), and thus, the highest accuracy.

The simulation environment developed in this research provides a tool to test strategies for dosing FE in AnMBRs. The integrated AnMBR model used in the simulation environment was developed, calibrated, and validated under specific conditions, that is, using the FE Adifloc KD451 in a specific pilot-scale AnMBR. To use the simulation environment under different conditions, the integrated model should be initially validated under those conditions.

### 3.5. Control Tools for Dosing Flux Enhancer

The integrated model was used as a simulation environment to test the various control tools presented in Table 5, for manipulating FE dosing to the pilot-scale AnMBR. The Simulink^®^ implementation is available in Simulink model S1, Supplementary Materials. The simulation environment included the biochemical-flocculation model described in Section 2.3, but with the mean particle size dynamics from Equation (44). As previously explained, the sludge filterability was predicted as $∆R_{20}$ with the AnDFCm filtration model D3 $\alpha_{c,2}$; the fouling rate was predicted with the empirical FR model FR6 and the FR_RIS model D1c $\alpha_{c,1p}$.

**Figure 6:**
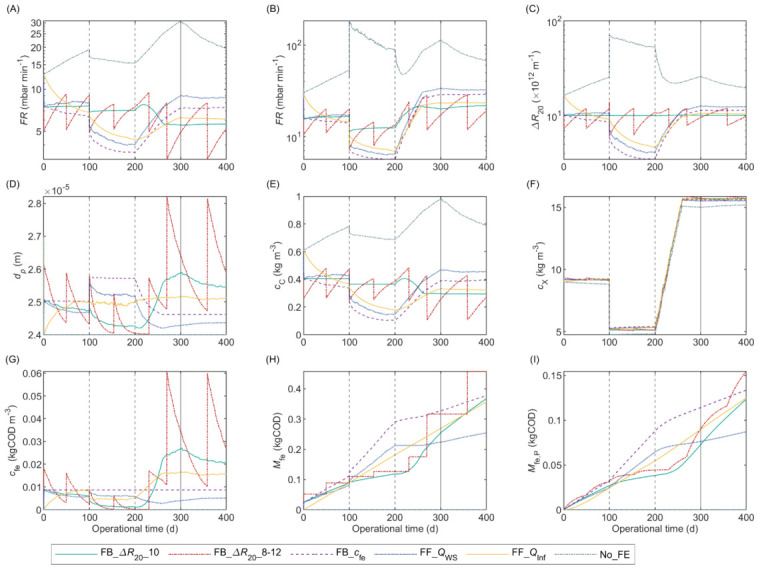
Simulated pilot-scale AnMBR behaviour with different feedback (FB) and feedforward (FF) control tools for manipulating the flux enhancer (FE) dosage. Compared variables: (**A**) Fouling rate with empirical model FR6; (**B**) fouling rate with RIS model D1c $\alpha_{c,1p}$; (**C**) sludge filterability expressed as $∆R_{20}$; (**D**) mean particle diameter; (**E**) colloidal material concentration; (**F**) particulate material concentration; (**G**) total FE concentration inside the reactor; (**H**) cumulative mass of FE dosed; (**I**) cumulative mass of FE removed with permeate flow. The vertical lines indicate applied disturbances on: TSS setpoint (dotted) and $f_{C,\mathrm{WS}}$ (continuous).

The results in Figure 6 show that all control tools substantially improved reactor performance by decreasing $∆R_{20}$ and membrane fouling as compared with the reactor without FE dosing (No_FE). The decrease was caused by FE-induced flocculation which reduced the concentration of colloidal material and increased the floc size.

The total mass of FE added in the 400-day simulated period varied between the different control tools, the lowest and highest amounts were 0.25 and 0.46 kg for FF_$Q_{\mathrm{WS}}$ and FB_$∆R_{20}$_8–12, respectively. Considering the base FE price given by the supplier of Adifloc KD451 of 6 € kg^−1^, the FE cost was between 1.37 and 2.50 € y^−1^, or 0.49 and 0.89 € m^−3^ y^−1^, which is negligible. Nevertheless, the costs of FE dosing can vary considerable for different AnMBRs [26].

The feedback $∆R_{20}$ control tool FB_$∆R_{20}$_8–12 was the tool that required the most FE due to the higher loss of FE with the permeate, shown in Figure 6I. This was because high amounts of FE were dosed in a short period, elevating the concentration of unbonded FE ($S_{\mathrm{fe}}$) which passed through the membrane and left the reactor with the permeate flow. Additionally, as expected, FB_$∆R_{20}$_8–12 caused less stable filterability and fouling rate than FB_$∆R_{20}$_10. Accordingly, continuous dosing the FE MPE50 to a pilot MBR caused more stable time-to-filter values and used less FE than applying periodic pulses; the pulses were applied whenever the time-to-filter was 200 s^−1^ [12]. These strategies were analogous to FF_$Q_{\mathrm{WS}}$ and FB_$∆R_{20}$_8–12, respectively.

We assumed $c_{\mathrm{fe}}$ for FB_$∆R_{20}$_10 in Figure 6G as the optimal FE dosage required to sustain a good and stable sludge filterability inside the reactor ($D_{\mathrm{opt}}$). This dosage varied between 1 and 27 mgCOD L^−1^ during the simulated period due to changes in sludge characteristics. Consequently, the $c_{\mathrm{fe}}\;$control tool (namely FB_$c_{\mathrm{fe}}$, FF_$Q_{\mathrm{WS}}$, and FF_$Q_{\mathrm{Inf}}$), which targeted a specific $c_{\mathrm{fe},\mathrm{sp}}$ of 8.7 mgCOD L^−1^, under- or overdosed FE during certain periods. For example, at high $c_{X}$ (250–400 d) more FE was required to achieve similar $c_{C}$ reductions, because the FE was adsorbed onto the particulate material, thereby, decreasing its availability for colloidal material flocculation. Here, the $c_{\mathrm{fe}}\;$control tools underdosed FE causing an increased $∆R_{20}$ and fouling rate as compared with FB_$∆R_{20}$_10. Conversely, at low $c_{X}$ (100–200 d), the FE required was lowered, and the $c_{\mathrm{fe}}\;$control tools overdosed FE increasing the FE concentration in the permeate and using unnecessary FE.

## 4. Conclusions

In this research, a comprehensive integrated model was developed and applied to test and optimise the dosing strategy of flux enhancer to an AnMBR. The integrated model was composed of the following models: biochemical-flocculation, AnMBR filtration, and AnDFCm filtration. These models were developed, calibrated, and validated separately, using experimental data from a pilot-scale AnMBR. The biochemical-flocculation model satisfactorily predicted the dynamics of mean particle diameter and colloidal material concentration. Nevertheless, the long-term model prediction requires further validation. For the filtration models, several alternate models were compared, and the best alternatives were selected based on model accuracy and capacity of the model to predict the effect of varying sludge characteristics on the corresponding output, that is, fouling rate or $∆R_{20}$. The selected model to predict sludge filterability was the AnDFCm filtration model D3 $\alpha_{c,2}$. To predict fouling rate, the selected models were the empirical FR model FR6 and the FR_RIS model D1c $\alpha_{c,1p}$, which covered a predicted fouling rate range. The concentration of colloidal material was an appropriate linking variable between the biochemical-flocculation models and filtration models for fouling rate and $∆R_{20}$ prediction, whereas the mean particle diameter was only appropriate for fouling rate prediction, but it worsened $∆R_{20}$ prediction. To improve model calibration and validation, better and additional input data is required, particularly, online gas flow measurements and intensive and comprehensive monitoring of sludge and blackwater characteristics.

The integrated calibrated model was used as a simulation environment to optimise the flux enhancer dosing strategy in a pilot-scale AnMBR. The preferred control tool to manipulate flux enhancer dosing was the feedback $∆R_{20}$ proportional controller, referred to as $∆R_{20}$_10. Furthermore, continuous flux enhancer dosing performed better than periodically dosing flux enhancer in the form of pulses. Continuous dosing decreased permeate contamination by flux enhancer, required a lower amount of flux enhancer, and achieved more stable sludge filterability and fouling rate.

## Figures and Tables

**Table 1:** Specific cake resistance (SCR, $\alpha_{c}$) submodels and the effects accounted for in each submodel.

SCR Submodel	Equation	Effects Considered		
		Colloidal Material	Particle Size	Compression, TMP
$\alpha_{c,1}$	$\frac{k_{\mathrm{CK}}\;\left(1-\varepsilon_{c}\right)}{\rho_{X}\;d_{p}^{2}\;\varepsilon_{c}{}^{3}}$	X	X	
$\alpha_{c,1p}$	$\frac{\alpha_{c,1}}{\left(1-\frac{J\mu \omega_{X}\alpha_{c,1}}{P_{a}}\right)}$	X	X	X
$\alpha_{c,2}$	$\frac{k_{c}\;{\left(1-\varepsilon_{c}\right)}^{2}}{\rho_{X}\varepsilon_{c}{}^{3}\left(1-\varepsilon_{c0}\right)}$	X		
$\alpha_{c,2p}$	$\frac{\alpha_{c,2}}{\left(1-\frac{J\mu \omega_{X}\alpha_{c,2}}{P_{a}}\right)}$	X		X
$\alpha_{c,3}$	$\frac{P_{b}}{\mu^{2}}\left(\zeta_{1}+\zeta_{2}{\left(1-\exp\left(-\zeta_{3}\frac{\omega_{C}}{\omega_{X}}\right)\right)}^{\zeta_{4}}\right)$	X		
$\alpha_{c,3p}$	$\frac{\alpha_{c,3}}{\left(1-\frac{J\mu \omega_{X}\alpha_{c,3}}{P_{a}}\right)}$	X		X
$\alpha_{c,4p}$	$R_{m}{\left(\frac{\mu }{J\left(\zeta_{1}+\zeta_{2}{\left(1-\exp\left(-\zeta_{3}\frac{\omega_{C}}{\omega_{X}}\right)\right)}^{\zeta_{4}}\right)}-\omega_{X}\right)}^{-1}$	X		X

**Table 2:** Stoichiometric coefficients and kinetic expressions for material deposition onto the membrane in alternate deposition submodels (D1 and D2).

Component i → Process j ↓		$\omega_{X}$ Deposited Particulate	$\omega_{C}$ Deposited Colloidal	$c_{X}$ Particulate in Bulk Liquid	$c_{C}$ Colloidal in Bulk Liquid	Deposition Submodel 1 (D1) (Drag Forces)		Deposition Submodel 2 (D2) (Drag and Lift Forces)	
1	Attachment of particulate material	1		−1		$J_{20}\;c_{X}$	(23)	$\frac{24\;J}{24\;J\;+\;C_{d}d_{p}G}Jc_{X}$	(24)
2	Detachment of particulate material by biogas sparging	−1		1		$q_{m,\mathrm{MS}}\frac{\omega_{X}}{K_{S,c}/A_{m}\;+\;\omega_{X}}I_{\mathrm{MS}}\frac{u_{G}}{H_{\mathrm{MT}}}\omega_{X}$	(25)	$\frac{\beta_{\mathrm{ST}}\;G\;\omega_{X}}{\gamma V_{F}\;+\;\omega_{X}}\omega_{X}$	(26)
3	Attachment of colloidal material		1		−1	$J_{20}\;c_{C}$	(27)	$f_{C,c}\;J\;c_{C}$	(28)
4	Detachment of colloidal material by biogas sparging		−1		1	$q_{m,\mathrm{MS}}\frac{\omega_{X}}{K_{S,c}/A_{m}\;+\;\omega_{X}}I_{\mathrm{MS}}\frac{u_{G}}{H_{\mathrm{MT}}}\omega_{C}$	(29)	$\frac{\beta_{\mathrm{ST}}\;G\;\omega_{X}}{\gamma V_{F}\;+\;\omega_{X}}\omega_{C}$	(30)

**Table 3:** Sigmoid inhibition function ($I_{\mathrm{MS}}$) equations for alternate deposition submodels (D1a, D1b, and D1c).

Deposition Submodel	$I_{MS}$ Equation	
D1a	$I_{\mathrm{MS}}={\left(1+FR\right)}^{-1}$ with $FR=K_{F}e^{J_{20}\left(\gamma_{0}\;-\;\gamma_{1}\frac{u_{G}}{H_{\mathrm{MT}}}\;+\;\gamma_{2}c_{X}\right)}$	(31)
D1b	$I_{\mathrm{MS}}={\left(1+FR\right)}^{-1}$ with $FR=K_{F}e^{J_{20}\left(\gamma_{0}\;-\;\gamma_{1}\frac{u_{G}}{H_{\mathrm{MT}}}\;+\;\gamma_{2}c_{X}\;+\;\gamma_{3}c_{C}\right)}$	(32)
D1c	$I_{\mathrm{MS}}=1$	(33)

**Table 4:** Alternate empirical FR models.

FR Model	$I_{MS}\mathrm{Equation}$	
1	$FR=K_{F}e^{J_{20}\left(\gamma_{0}-\gamma_{1}\frac{u_{G}}{H_{\mathrm{MT}}}+\gamma_{2}c_{X}+\gamma_{3}c_{C}\right)}$	(32)
2	$FR=K_{F}e^{J_{20}\left(\gamma_{0}-\gamma_{1}\frac{u_{G}}{H_{\mathrm{MT}}}+\gamma_{3}c_{C}\right)}$	(37)
3	$FR=f_{\mathrm{conv}\;}K_{F\;}u_{G}^{-\gamma_{G}}e^{J_{20}\left(\gamma_{0}+\gamma_{3}c_{C}\right)}$	(38)
4	$FR=K_{F}e^{J_{20}\left(\gamma_{0}-\gamma_{1}\frac{u_{G}}{H_{\mathrm{MT}}}+\gamma_{2}c_{X}+\gamma_{3}c_{C}-\gamma_{4}d_{p}\right)}$	(39)
5	$FR=K_{F}e^{J_{20}\left(\gamma_{0}-\gamma_{1}\frac{u_{G}}{H_{\mathrm{MT}}}+\gamma_{3}c_{C}-\gamma_{4}d_{p}\right)}$	(40)
6	$FR=f_{\mathrm{conv}\;}K_{F\;}u_{G}^{-\gamma_{G}}e^{J_{20}\left(\gamma_{0}+\gamma_{3}c_{C}-\gamma_{4}d_{p}\right)}$	(41)

**Table 5:** Control tools to manipulate the mass flow rate of flux enhancer (${\dot{m}}_{\mathrm{fe}}$) to an AnMBR.

Reference	Type of Control	Measured Variable	Controlled Variable	${\dot{m}}_{fe}$ Calculation
FB_$∆R_{20}$_10	Feedback, proportional	$∆R_{20}$	$∆R_{20}$	1.6 × 10^−7^ $\left(∆R_{20}-∆R_{20,\mathrm{sp}}\right)$
FB_$∆R_{20}$_8–12	Feedback, on-off	$∆R_{20}$	$∆R_{20}$	5 × 10^−6^
FB_ $c_{\mathrm{fe}}$	Feedback, proportional	$c_{\mathrm{fe}}$	$c_{\mathrm{fe}}$	1 × 10^−3^ $\left(c_{\mathrm{fe},\mathrm{sp}}-c_{\mathrm{fe}}\right)$
FF_ $Q_{\mathrm{WS}}$	Feedforward, pulse + proportional ^1^	$Q_{\mathrm{WS}}$	$c_{\mathrm{fe}}$	$Q_{\mathrm{WS}}\;f_{\mathrm{Xfe},\mathrm{WS}\;}c_{\mathrm{fe},\mathrm{sp}}$
FF_ $Q_{\mathrm{Inf}}$	Feedforward, proportional	$Q_{\mathrm{Inf}}$	$c_{\mathrm{fe}}$	$Y_{\mathrm{fe},\mathrm{Inf}}\;Q_{\mathrm{Inf}}$
No_FE	No control	NA	NA	0

^1^ Initial pulse dosage to achieve the setpoint $c_{\mathrm{fe},\mathrm{sp}}$ followed by continuous additions to compensate for the loss of flux enhancer with sludge withdrawal.

## Data Availability

Not applicable.

## Supplementary Materials

The following are available online at https://www.mdpi.com/article/10.3390/membranes12020151/s1, Section S1: Supplementary pilot-scale AnMBR experimental data, Section S2: Supplementary experiments, Section S3: Supplementary material for the biochemical-flocculation model, Section S4: Parameter values, Section S5: Model calibration procedure, Section S6: Statistical indicators representing model accuracy, Section S7: Simulated influent characteristics and applied disturbances, Section S8: Calibration and validation of the biochemical-flocculation model, Section S9: Calibration and validation of alternate AnMBR filtration models, Section S10: Effect of sludge characteristics on fouling rate predictions, Section S11: Calibration and validation of alternate AnDFCm filtration models, Section S12: Effect of sludge characteristics on filterability predictions, Section S13: Cake layer compression, Section S14: Nomenclature, Simulink Model S1: Simulink^®^ implementation of control tools for flux enhancer dosing to the pilot-scale AnMBR.
