# Supplementary material for: Optimising the Flux Enhancer Dosing Strategy in a Pilot-Scale Anaerobic Membrane Bioreactor by Mathematical Modelling
Source: Membranes (Basel). 2022 Jan 26;12(2):151. doi: 10.3390/membranes12020151 (PMC8877340; doi:10.3390/membranes12020151)
Supplement: Supplementary file 1 [file membranes-12-00151-s001.zip › Supplementary file s1.pdf]

membranes

MDPI

Supplementary material

Optimising the flux enhancer dosing strategy in a pilot-scale anaerobic membrane bioreactor by mathematical modelling

Magela Odriozola*, Jules B. van Lier, Henri Spanjers

Delft University of Technology, Department of Water Management, Stevinweg 1, 2628 CN Delft, the Netherlands.

*Corresponding author: m.odriozolaarbiza@tudelft.nl.

Table of contents

S1. Supplementary pilot AnMBR experimental data 2

S2. Supplementary experiments 8

S3. Supplementary material for biochemical-flocculation model 11

S4. Parameter values 13

S5. Model calibration procedure 20

S6. Statistical indicators representing model accuracy 21

S7. Simulated influent characteristics and applied disturbances 22

S8. Calibration and validation of biochemical-flocculation model 23

S9. Calibration and validation of alternate AnMBR filtration models 27

S10. Effect of sludge characteristics on fouling rate predictions 52

S11. Calibration and validation of alternate AnDFCm filtration models 54

S12. Effect of sludge characteristics on filterability predictions 66

S13. Cake layer compression 67

S14. Nomenclature 69

References 75

S1. Supplementary pilot AnMBR experimental data

Pilot AnMBR description and monitoring

The pilot AnMBR plant described in Odriozola et al. [1], was used for model calibration and validation. Table S1 summarises the characteristics and operational conditions of the pilot AnMBR plant. The 20°C-normalised transmembrane flux (J_{20}) was calculated with the motor frequency of the permeate pump (v_{P}), using Equation (S.1) based on a flux-step experiment performed in the pilot AnMBR.

$$J_{20} = {(0.154\ v_{\mathrm{P}} + 0.733)}/{3.6e6} \tag{S.1}$$

The transmembrane flux (J) was calculated to reflect the permeate viscosity dependence on temperature with Equation (S.2), where μ_{20} is the permeate viscosity at 20°C and μ the permeate viscosity at the operational temperature.

$$J = J_{20}\frac{\mu_{20}}{\mu} \tag{S.2}$$

The flow rates of blackwater (Q_{Inf}) and permeate (Q_{P}), which were used in the biochemical-flocculation model, were calculated with a time-step (Δt) of 864 s (0.01 days). Q_{P} was the ratio between the volume of permeate produced during one time-step (ΔV_{P}) and Δt, and Q_{Inf} is calculated applying a mass balance in the liquid phase of the pilot AnMBR, as follows:

$$Q_{\mathrm{P}} = \frac{\Delta V_{\mathrm{P}}}{\Delta t} \tag{S.3}$$

$$Q_{\mathrm{Inf}} = Q_{\mathrm{P}} + Q_{\mathrm{WS}} - Q_{\mathrm{fe}} + \frac{\Delta V_{\mathrm{L}}}{\Delta t} \tag{S.4}$$

, where Q_{WS} and Q_{fe} are the flow rates of wasted sludge and flux enhancer, respectively, ΔV_{L} the difference in V_{L} between time-steps and V_{L} the total mixed liquor volume.

Grab samples of sludge, blackwater and permeate were regularly taken from the AnMBR for characterisation. Table S2 and Figure S1 show the physicochemical characteristics used for calibration of the biochemical-flocculation model. During days 13 to 18, TSS accumulated in the membrane tank due to malfunctioning of the sludge recirculation pump, this period was not included in the calibration of the biochemical-flocculation model.

The fouling rate (FR) in the pilot AnMBR was measured as the change in transmembrane pressure (TMP) over time during each filtration cycle ($d\mathrm{TMP}/dt$), calculated with the following linear regression equation:

$$\mathrm{FR} = \frac{\mathrm{dTMP}}{\mathrm{dt}} \approx \frac{\mathrm{n}\sum_{\mathrm{i=1}}^{\mathrm{n}}(\mathrm{TMP_i\ t_i}) - \sum_{\mathrm{i=1}}^{\mathrm{n}}\mathrm{TMP_i}\ \sum_{\mathrm{i=1}}^{\mathrm{n}}\mathrm{t_i}}{\mathrm{n}\sum_{\mathrm{i=1}}^{\mathrm{n}}\mathrm{t_i}^2 - (\sum_{\mathrm{i=1}}^{\mathrm{n}}\mathrm{t_i})^2} \tag{S.5}$$

, where t_i and TMP_i are the time and corresponding TMP during one filtration cycle, and n is the number of observations. The fouling rate measured in the pilot AnMBR was used as model output during calibration and validation of the AnMBR filtration models. The sludge characteristics, c_{C}, c_{X}, T and d_p shown in Figure S1, and gas sparging intensity (Figure S2) were used as inputs during model calibration and validation. The total concentration of colloidal and particulate material in the mixed liquor expressed as suspended solids, c_{C} and c_{X}, respectively, were calculated as follows:

$$c_{\mathrm{C}} = \frac{\mathrm{csCOD} - \mathrm{pCOD}}{i_{\mathrm{COD,CI}}} \tag{S.6}$$

$$c_{\mathrm{X}} = \mathrm{TSS} - c_{\mathrm{C}} \tag{S.7}$$

, where $i_{\mathrm{COD,CI}}$ the theoretical COD of inert colloidal material, which was considered equal to the theoretical COD of biomass, that is 1.42 gCOD g^{-1} [2]. The values for csCOD, pCOD and TSS are the linearly interpolated values between measured values of each variable.

During the operation of the pilot, filterability was measured in-situ by connecting the AnDFCm installation in bypass to the pilot AnMBR, details are given in Odriozola et al. [1]. The ΔR_{20} was the output of the AnDFCm filtration models, and the sludge characteristics shown in Figure S1 were used as inputs during model calibration.

Flux enhancer was added to the pilot as follows: 138.5 g pulse input (M_{fe} = 0.1385 kgCOD) of the cationic polymer Adifloc KD451 (Adipap SA, France), was introduced to the bypass line of the pilot AnMBR on day 16 (t_{fe0} = 1,379,754 s) with an injection time (Δt_{fe}) of 2,700 s. Therefore, in the model, the FE mass flow rate ($\dot{m}_{\mathrm{fe}}$) was as follows:

$$\dot{m}_{\mathrm{fe}} = \begin{cases} 0, & t < t_{\mathrm{fe0}} \\ \dfrac{M_{\mathrm{fe}}}{\Delta t_{\mathrm{fe}}}, & t_{\mathrm{fe0}} \le t \le t_{\mathrm{fe0}} + \Delta t_{\mathrm{fe}} \\ 0, & t > t_{\mathrm{fe0}} + \Delta t_{\mathrm{fe}} \end{cases} \tag{S.8}$$

Table S1. Characteristics of the pilot AnMBR and operational conditions relevant for model calibration and validation.

Description	Nomenclature	Units	Mean value	Range
Cross-sectional area MT	A_{MT}	m^2	0.68	NA
Gas pressure in AR headspace	p_{G}	bar	1.03	[1.00, 1.06]
Gas superficial velocity in MT	u_{G}	$\times 10^{-3}$ m s^{-1}	3.0	[0.5,5.7]
Liquid level in MT	H_{MT}	m	1.42	[1.22, 1.56]
Membrane surface area	A_{m}	m^2	6.25	NA
Mixed liquor pH	pH	-	7.1	[6.8, 7.5]
Mixed liquor temperature	T	K	296	[292, 301]
Motor frequency blower	v_{B}	s^{-1}	59	[50, 60]
Motor frequency permeate pump	v_{P}	s^{-1}	60	[30, 60]
Flow rate influent	Q_{Inf}	$\times 10^{-5}$ m^3 s^{-1}	2.0	[0.9, 38]
Flow rate permeate	Q_{P}	$\times 10^{-5}$ m^3 s^{-1}	2.0	[0.9, 2.6]
Flow rate wasted sludge [a]	Q_{WS}	$\times 10^{-5}$ m^3 s^{-1}	~ 0	[0, 38]
Total filtration time in one cycle	θ_{F}	s	300	NA
Total relaxation time in one cycle	θ_{R}	s	90	NA
Total mixed liquor volume	V_{L}	m^3	2.7	[2.0, 2.9]
Transmembrane flux, 20°C-normalised	J_{20}	$\times 10^{-6}$ m^3 m^2 s^{-1}	2.7	[1.5, 2.8]
Transmembrane flux	J	$\times 10^{-6}$ m^3 m^2 s^{-1}	3.2	[1.7, 3.3]
Transmembrane pressure	TMP	$\times 10^3$ Pa	0.82	[0, 2.8]

Abbreviations: AR: anaerobic reactor; COD: chemical oxygen demand; MT: membrane tank; NA: not applicable.

[a] Sludge waste negligible during normal operation, except for a one-time sludge withdrawal of 31% of the mixed liquor performed on day 123.

Table S2. Blackwater, permeate and mixed liquor characteristics during pilot AnMBR operation.

Sample	Parameter	Nomenclature (for model)	Units	Mean value	Range
Blackwater	Alkalinity	Alk_{BW}	$kgCaCO_3\ m^{-3}$	0.69	[0.50, 0.82]
	Ammonium	$NH4_{BW}$	$kgN\ m^{-3}$	0.15	[0.10, 0.20]
	Submicron COD	$csCOD_{BW}$	$kg\ m^{-3}$	0.34	[0.12, 0.63]
	Total COD	$tCOD_{BW}$	$kg\ m^{-3}$	1.62	[0.7, 3.3]
Mixed liquor	Colloidal COD [a]	cCOD	$kg\ m^{-3}$	0.50	[0.20, 0.87]
	Mean particle diameter	d_p	$\times 10^5\ m$	2.7	[2.1, 4.5]
	Submicron COD	csCOD	$kg\ m^{-3}$	0.59	[0.30, 0.96]
	Total suspended solids	TSS	$kg\ m^{-3}$	9.6	[5.5, 16.0]
Permeate	Total COD	pCOD	$kg\ m^{-3}$	0.09	[0.05, 0.11]

[a] Calculated as the difference between the mixed liquor csCOD and the total permeate COD.

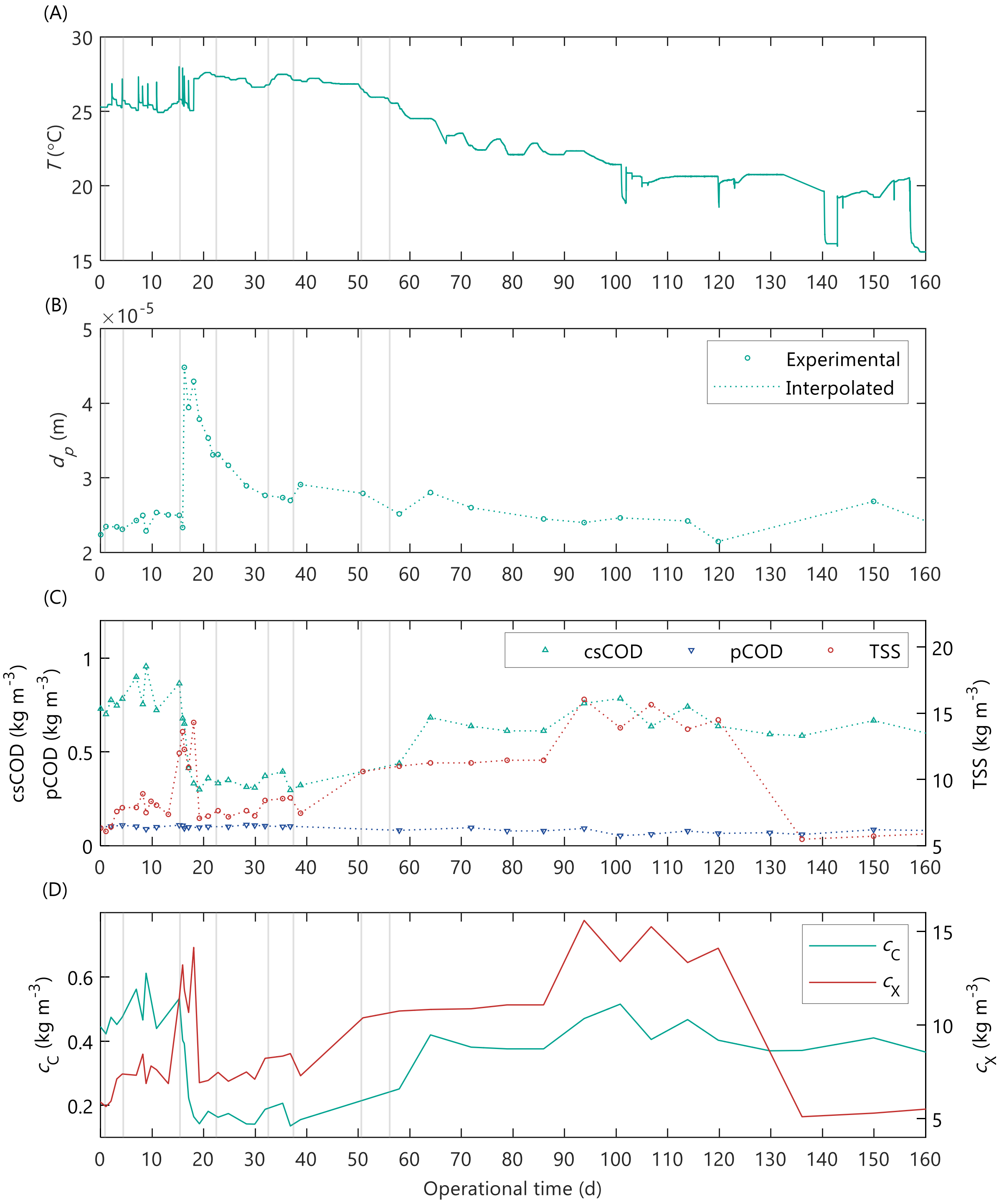

Figure S1. Pilot AnMBR mixed liquor characteristics used as inputs in the AnMBR and AnDFCm filtration models: (A) temperature measured by SCADA, (B) measured and interpolated mean particle diameter, (C) measured (markers) and interpolated (dotted lines) submicron COD, permeate COD and total suspended solids concentrations, and (D) calculated colloidal and particulate material concentrations. The grey-vertical areas represent the representative dataset, iD1 to iD8 from left to right, used for calibration of the filtration models.

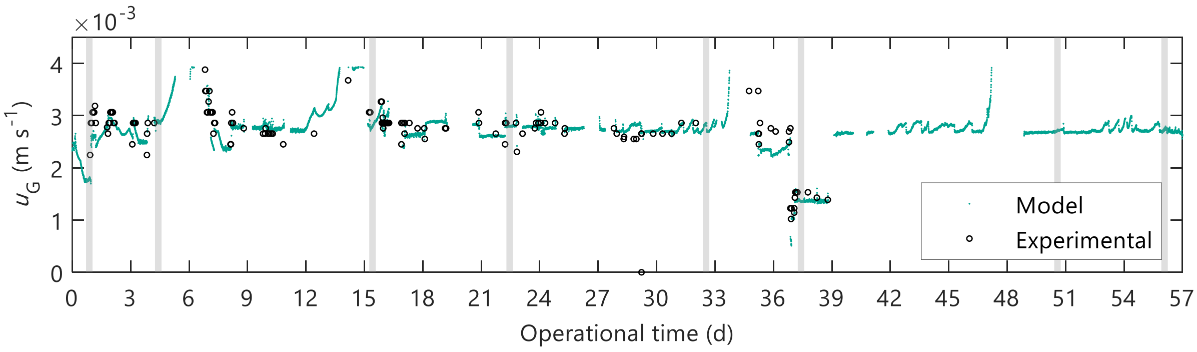

Figure S2. Gas superficial velocity in the pilot AnMBR membrane tank used as input variable in the AnMBR filtration models . The grey-vertical areas represent the representative dataset, iD1 to iD8 from left to right, used for calibration of the AnMBR filtration models.

Gas-step test in the pilot AnMBR

The effect of gas sparging on the fouling rate was assessed with gas-step experiments in the pilot AnMBR. The gas superficial velocity in the membrane tank (u_G) was stepwise decreased at the beginning of each relaxation cycle. The step height was -0.4×10^{-3} m s^{-1}, the maximum and minimum u_G were 2.7×10^{-3} and 0.6×10^{-4} m s^{-1}, respectively. The duration of the filtration and relaxation were 15 minutes.

The FR models in Table 4 were optimised to fit the experimental fouling rate measured during the gas-step test in the pilot AnMBR. The gas-step test was performed at constant J_{20}, H_{MT}, c_X and c_C; therefore, Equation (32) becomes $FR = K_F e^{(a_0 - a_1 u_G)}$, where a_0 and a_1 are parameters, with $a_1 = -\gamma_1 J_{20}/H_{MT}$ and $a_0 = J_{20}(\gamma_0 + \gamma_2 c_X)$; and Equation (38) becomes $FR = a_0\, u_G^{-\gamma_G}$ with $a_0 = f_{conv} K_F e^{J_{20}(\gamma_0 + \gamma_3 c_C)}$. The parameters were optimised to fit the experimental data using the function *fit* in Matlab®, the experimental and simulated results are displayed in **Figure S3**.

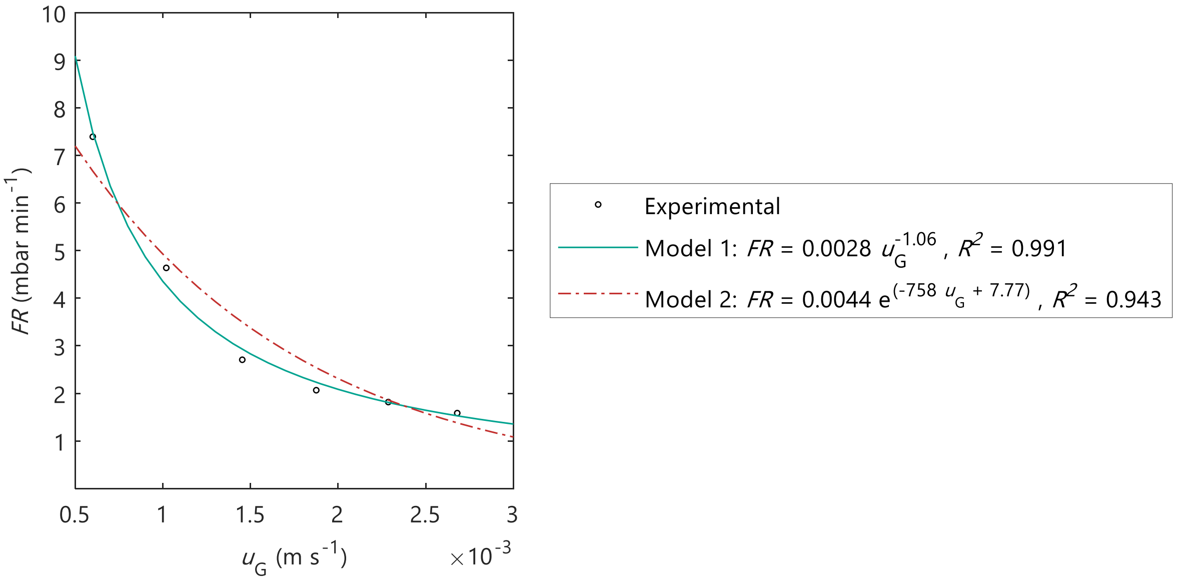

Figure S3. Experimental and simulated fouling rate during the gas-step experiment in the pilot AnMBR.

S2. Supplementary experiments

Batch flocculation kinetic experiments

Flocculation kinetics were assessed in batch assays by intensive monitoring the PSD as a function of time after FE dosing into a grab sludge sample. PSD was measured with a Microtrac Bluewave diffraction analyser (Malvern Instruments Ltd., UK). The sludge samples were collected from a full-scale anaerobic digester treating primary and secondary sludge of a sewage treatment plant (Harnaschpolder, Den Hoorn, The Netherlands). The tests were performed in a jar-test apparatus with a mixing speed of 90 rpm and under dosing of 0.1, 0.2 and 0.3 g L^{-1} of Adifloc KD451, with two replicates per dosage. The sludge was premixed for 10 minutes to homogenise the sample before FE dosing.

The FE caused a rapid increase in the median particle size followed by a gradual decrease reaching a steady value, Figure S4. The time to reach steady values was higher at higher FE concentrations, varying from 10 to 90 minutes.

The FE adsorption rate coefficient (k_{ads}) was estimated as the inverse of the flocculation time, thus, k_{ads} was between 16 and 144 d^{-1}, and a nominal value of 48 d^{-1} was assumed (equivalent to 30 minutes). Similarly, researchers achieved equilibrium conditions after mixing for 30 minutes for the absorption of polyDADMAC onto waste activated sludge [3] and onto cellulosic fibres [4].

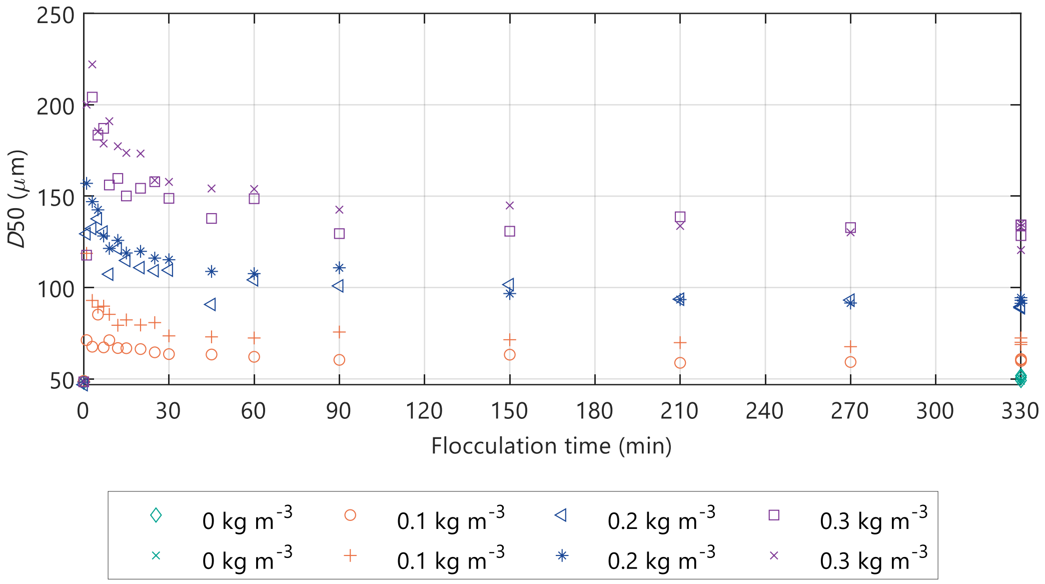

Figure S4. Median particle diameter immediately after flux enhancer addition at different concentrations into a sludge grab sample from full-scale digester. The experiments were performed in a jar test apparatus continuously mixed at 90 rpm. Duplicate measurements have the same colour.

Sludge viscosity in the AnDFCm installation

We determine the dynamic viscosity of sludge samples at different sludge concentrations using the AnDFCm installation operated at 1.5 m s^{-1} crossflow velocity. Five sludge samples were collected from three different AnMBRs and one anerobic digester, the total suspended solids (TSS) of the samples ranged from 3 to 30 g L^{-1}.

The dynamic viscosity of the mixed liquor (μ_L) was calculated using the experimentally measured pressure drop along membrane in the AnDFCm installation (Δp) [5,6]. μ_L was calculated with Equation (S.9) which combines Euler's and Darcy-Weisbach equations for energy loss in a pipe and the empirical formula that relates the Darcy-Weisbach friction factor with the Reynold's number (Re) for laminar flow (Re<2300).

$$\mu_L = \frac{D_m^2}{32 u_L}\left(-\rho_L\, g + \frac{\Delta p}{\Delta L}\right) \quad (S.9)$$

, where D_m is the membrane internal diameter (0.008 m), u_L the fluid crossflow velocity (1.5 m s^{-1}), ρ_L the fluid density, g the gravitational acceleration, and ΔL the height difference (0.95 m, membrane length). We assumed ρ_L as equal to the density of water (ρ_W) at the operational temperature T, calculated as follows:

$$\rho_W = -0.0033\, T^2 - 0.1048\, T + 1001.5 \quad (S.10)$$

The Re was calculated with Equation (S.11) to assess the rheological conditions, where laminar flow corresponds to Re <2300, and turbulent to Re > 4000.

$$Re = \frac{\rho_L u_L D_m}{\mu_L} \quad (S.11)$$

The viscosity of sludge sample with different TSS was measured in the AnDFCm installation, results are show in Table S3. All sludges presented laminar flow (Re<2300), which was assumed during viscosity calculation in Equation (S.9).

The parameters of the mixed liquor viscosity model, a and b in Equation (36), were estimated with the function fit in Matlab®. The calibrated model was as follows:

$$\frac{\mu_L}{\mu_W} = 9.35\, e^{0.047\ \mathrm{TSS}} \quad (S.12)$$

The water viscosity (μ_W) was calculated at each sludge temperature with Equation (S.10). Figure S5 displays the experimental and simulated data.

Table S3. Sludge characteristics and viscosity calculations.

Sludge	TSS (g L^{-1})	T (°C)	Δp (Pa)	Re	μ_L (Pa s)
1	3	17.3	18,334	1,047	0.0113
2	5	21.5	17,902	1,104	0.0108
3	10	24.3	19,693	978	0.0127
4	15	30.2	22,198	728	0.0165
5	30	29.5	33,202	400	0.0305

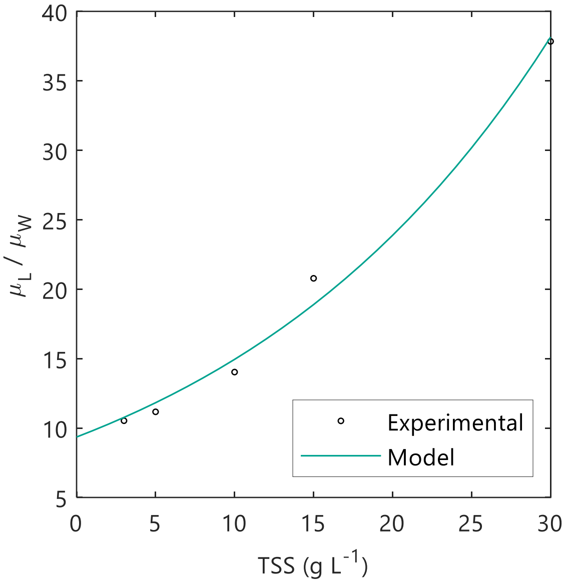

Figure S5. Model calibration of the mixed liquor apparent viscosity (μ_L), normalised by water viscosity (μ_W), in the AnDFCm installation for sludges with different concentrations of total suspended solids (TSS).

S3. Supplementary material for biochemical-flocculation model

Table S4. Process rate equations (ρ_j) and stoichiometric coefficients ($\upsilon_{i,j}$) of the biochemical-flocculation model.

Component →	i	1	2	3	4	5	6	7	8	9	10	11	12	13	14	15	16	17	18	19	20	21	22	23	24	25	26	27	28	29	30	Rate ρ_j (kgCOD m^{-3} d^{-1}) (ρ_j for j=19-21 kmol m^{-3} d^{-1})
j	Process ↓	S_{su}	S_{aa}	S_{fa}	S_{va}	S_{bu}	S_{pro}	S_{ac}	S_{h2}	S_{ch4}	S_{IC}	S_{IN}	S_I	S_{fe}	X_{ch}	X_{pr}	X_{li}	X_{su}	X_{aa}	X_{fa}	X_{c4}	X_{pro}	X_{ac}	X_{h2}	X_I	C_I	X_{fe}	X_{bio}	$c_{h2,G}$	$c_{ch4,G}$	$c_{co2,G}$	
1	Hydrolysis of Carbohydrates	1													-1																	$k_{hyd,ch}X_{ch}$
2	Hydrolysis of Proteins		1													-1																$k_{hyd,pr}X_{pr}$
3	Hydrolysis of Lipids	1 - $f_{fa,li}$		$f_{fa,li}$													-1															$k_{hyd,li}X_{li}$
4	Uptake of Sugars	-1				$(1-Y_{su})f_{bu,su}$	$(1-Y_{su})f_{pro,su}$	$(1-Y_{su})f_{ac,su}$	$(1-Y_{su})f_{h2,su}$		$-\sum_{i\neq10} i_{C,i}\nu_{i,su}$	$-Y_{su}i_{N,su}$						Y_{su}														$k_{m,su}\frac{S_{su}}{K_{s,su}+S_{su}}X_{su}I_{1,su}$
5	Uptake of Amino Acids		-1		$(1-Y_{aa})f_{va,aa}$	$(1-Y_{aa})f_{bu,aa}$	$(1-Y_{aa})f_{pro,aa}$	$(1-Y_{aa})f_{ac,aa}$	$(1-Y_{aa})f_{h2,aa}$		$-\sum_{i\neq10} i_{C,i}\nu_{i,aa}$	$-Y_{aa}i_{N,aa}$							Y_{aa}													$k_{m,aa}\frac{S_{aa}}{K_{s,aa}+S_{aa}}X_{aa}I_{1,aa}$
6	Uptake of LCFA			-1				$(1-Y_{fa})0.7$	$(1-Y_{fa})0.3$			$-Y_{fa}i_{N,fa}$								Y_{fa}												$k_{m,fa}\frac{S_{fa}}{K_{s,fa}+S_{fa}}X_{fa}I_{2,fa}$
7	Uptake of Valerate				-1		$(1-Y_{c4})0.54$	$(1-Y_{c4})0.31$	$(1-Y_{c4})0.15$			$-Y_{c4}i_{N,c4}$									Y_{c4}											$k_{m,c4}\frac{S_{va}}{K_{s,c4}+S_{va}}X_{c4}\frac{1}{1+S_{bu}/S_{va}}I_{2,c4}$
8	Uptake of Butyrate					-1		$(1-Y_{c4})0.8$	$(1-Y_{c4})0.2$			$-Y_{c4}i_{N,c4}$									Y_{c4}											$k_{m,c4}\frac{S_{bu}}{K_{s,c4}+S_{bu}}X_{c4}\frac{1}{1+S_{va}/S_{bu}}I_{2,c4}$
9	Uptake of Propionate						-1	$(1-Y_{pro})0.57$	$(1-Y_{pro})0.43$		$-\sum_{i\neq10} i_{C,i}\nu_{i,pro}$	$-Y_{pro}i_{N,pro}$										Y_{pro}										$k_{m,pro}\frac{S_{pro}}{K_{s,pro}+S_{pro}}X_{pro}I_{2,pro}$
10	Uptake of Acetate							-1		$1-Y_{ac}$	$-\sum_{i\neq10} i_{C,i}\nu_{i,ac}$	$-Y_{ac}i_{N,ac}$											Y_{ac}									$k_{m,ac}\frac{S_{ac}}{K_{s,ac}+S_{ac}}X_{ac}I_{3,ac}$
11	Uptake of Hydrogen								-1	$1-Y_{h2}$	$-\sum_{i\neq10} i_{C,i}\nu_{i,h2}$	$-Y_{h2}i_{N,h2}$												Y_{h2}								$k_{m,h2}\frac{S_{h2}}{K_{s,h2}+S_{h2}}X_{h2}I_{1,h2}$
12	Decay of X_{su}																	-1										1				$b_{su}X_{su}$
13	Decay of X_{aa}																		-1									1				$b_{aa}X_{aa}$
14	Decay of X_{fa}																			-1								1				$b_{fa}X_{fa}$
15	Decay of X_{c4}																				-1							1				$b_{c4}X_{c4}$
16	Decay of X_{pro}																					-1						1				$b_{pro}X_{pro}$
17	Decay of X_{ac}																						-1					1				$b_{ac}X_{ac}$
18	Decay of X_{h2}																							-1				1				$b_{h2}X_{h2}$
19	Disintegration of decayed biomass												$f_{SI,bio}$		$f_{ch,bio}$	$f_{pr,bio}$	$f_{li,bio}$								$f_{XI,bio}$	$f_{CI,bio}$		-1				$k_{dis,bio}X_{bio}$
20	Liquid-gas transfer of H_2								-1																				1/16			$k_La(S_{h2}-K_{H,h2}\,c_{h2,G}RT)$
21	Liquid-gas transfer of CH_4									-1																				1/64		$k_La(S_{ch4}-K_{H,ch4}\,c_{ch4,G}RT)$
22	Liquid-gas transfer of CO_2										-1																				1	$k_La(S_{co2}-K_{H,ch4}c_{co2,G}RT)$
23	Adsorption of flux enhancer onto particulate material													-1													1					$k_{ads}(X_{fe,e}-X_{fe})$
24	Flocculation of colloidal material																								1	-1						$Y_{fe,C}\,k_{ads}(X_{fe,e}-X_{fe})\frac{C_I/i_{COD,CI}}{c_X+\ c_C}\frac{X_I}{X_I+1\times10^{-6}}$
Component → (kgCOD m^{-3})		Monosaccharides	Amino Acids	Long chain fatty acids (LCFA)	Total Valerate	Total Butyrate	Total Propionate	Total Acetate	Hydrogen gas	Methane gas	Inorganic Carbon (kmol m^{-3})	Inorganic Nitrogen (kmol m^{-3})	Soluble inerts	Flux enhancer (in bulk liquid)	Carbohydrates	Proteins	Lipids	Sugar degraders	Amino acid degraders	LCFA degraders	Valerate and butyrate degraders	Propionate degraders	Acetate degraders	Hydrogen degraders	Particulate inerts	Colloidal inerts	Flux enhancer (adsorbed)	Decayed biomass	Hydrogen in gas phase (kmol m^{-3})	Methane in gas phase (kmol m^{-3})	Carbon dioxide in gas phase (kmol m^{-3})	Inhibition factors:<br>$I_{1,j}=I_{ph,j}\,I_{IN,lim}$<br>$I_{2,j}=I_{ph,j}\,I_{IN,lim}\,I_{h2,j}$<br>$I_{3,j}=I_{ph,j}\,I_{IN,lim}\,I_{fe}$<br>$I_{ph,j}=\frac{1+2\times10^{0.5(pH_{LL,j}-pH_{UL,j})}}{1+10^{(pH-pH_{UL,j})}+10^{(pH_{LL,j}-pH)}}$<br>$I_{IN,lim}=\frac{S_{IN}}{K_{s,IN}+S_{IN}}$<br>$I_{h2,j}=\frac{1}{1+S_{h2}/K_{I,h2,j}}$<br>$I_{fe}=\frac{1}{1+S_{fe}/K_{I,fe}}$

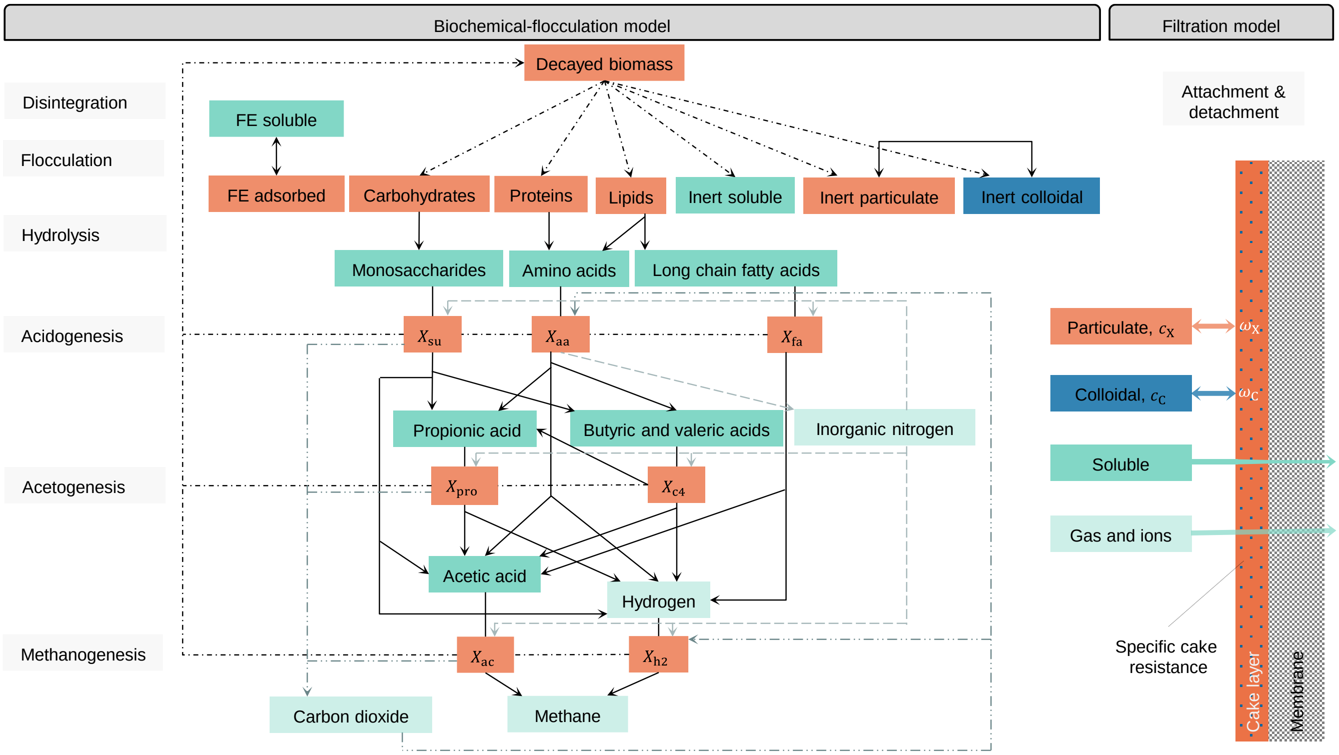

Figure S6. Scheme of the modified ADM1, extended to include processes caused by the addition of flux enhancer (FE), coupled with filtration model. X_i is the degrader of component i; c_X and c_C are the total concentration of particulate and colloidal materials in the bulk liquid, respectively; and ω_X and ω_C are the mass of particulate and colloidal materials deposited in the membrane, respectively.

S4. Parameter values

Biochemical-flocculation model

Most parameter values were taken from literature. The reaction rate for process j (k_j) was calculated at the operational temperature as follows [7]:

$$k_j\Big|_T = k_j\Big|_{T_{\mathrm{ref}}} \theta_j^{(T-T_{\mathrm{ref}})} \tag{S.13}$$

, where θ_j is the temperature correction factor for k_j, and T_{ref} the reference temperature. The reaction rate k_j is: the first order reaction rate coefficient for hydrolysis ($k_{\mathrm{hyd},j}$) for $j \in [1,3]$, the Monod maximum specific uptake rate ($k_{m,j}$) for $j \in [4,11]$, the first order decay rate of microorganism (b_j) for $j \in [12,18]$, and the first order reaction rate coefficient for biomass disintegration ($k_{\mathrm{dis,bio}}$) for $j = 19$. Table S5 summarises the values for k_j at T_{ref} =308.15 K and θ_j.

Disintegration and hydrolysis are the rate limiting steps in anaerobic digestion and have a high variability and sensitivity [8]. Thus, we included the biomass disintegration ($k_{\mathrm{dis,bio}}$) and hydrolysis rate coefficients in the model calibration. To simplify the calibration, a unique hydrolysis rate coefficient (k_{hyd}) was considered for all hydrolysis processes, which seems to be warranted considering the low loading rates applied to the pilot AnMBR [9]. Previous research reported rate limiting values for primary sludge or blackwater ranging from 0.0096 to 4.5 d^{-1} [8,10–12]. Therefore, the range for global sensitivity analysis (GSA) was set from 0.0096 to 4.5 for k_{hyd} and $k_{\mathrm{dis,bio}}$, as shown in Table S6. The nominal value for k_{hyd} was the value for disintegration in the ADM1 which is rate limiting compared to the proposed hydrolysis rates. For $k_{\mathrm{dis,bio}}$ the nominal value was the limiting rate for biomass degradation and hydrolysis [13].

Table S5 shows the values of the following stoichiometric and inhibitions parameters: Monod half saturation coefficient for process j ($K_{S,j}$), Monod half saturation coefficient for inorganic nitrogen ($K_{S,\mathrm{IN}}$), concentration of inhibitor i giving 50% inhibition on process j ($K_{I,i,j}$), empirical upper ($\mathrm{pH}_{\mathrm{UL},j}$) and lower ($\mathrm{pH}_{\mathrm{LL},j}$) pH inhibition coefficients, and yield coefficient of biomass on substrate for process j (Y_j).

Table S7 shows the carbon and nitrogen content in the different components and the theoretical chemical oxygen demand used to calculate the total concentration of particulate and colloidal material expressed as suspended solids.

The gas-liquid transfer coefficient ($k_L a$) for oxygen, 178 d^{-1} [14], was used for all gases. Henry's law coefficients were calculated at the operational temperature with Equation (S.14) using the values at T_{ref}. The $K_{H,i}$ at 308.15 K were 0.012 kgCOD m^{-3} bar^{-1}, 0.108 kgCOD m^{-3} bar^{-1}, and 0.027 kmol m^{-3} bar^{-1}, and $\theta_{H,i}$ were 525, 1744, 2405 for hydrogen, methane and carbon dioxide, respectively [15].

$$K_{H,i}\Big|_T = K_{H,i}\Big|_{T_{\mathrm{ref}}} e^{\theta_{H,i}\left(\frac{1}{T}-\frac{1}{T_{\mathrm{ref}}}\right)} \tag{S.14}$$

Table S5. Kinetic and stoichiometric parameter values for biochemical reactions at 35°C.

j	k_j [a] (d^{-1})	θ_j (-)	$K_{S,j}$ (kgCOD m^{-3})	$K_{S,\mathrm{IN}}$ (M)	$K_{I,\mathrm{h2},j}$ (kgCOD m^{-3})	$K_{I,\mathrm{fe},j}$ (kgCOD m^{-3})	$\mathrm{pH}_{\mathrm{UL},j}$ (-)	$\mathrm{pH}_{\mathrm{LL},j}$ (-)	Y_j (-)
1	0.5 [b]	1.066							
2	0.5 [b]	1.066							
3	0.5 [b]	1.066							
4	30	1.033	0.5	1×10^{-4}			5.5	4	0.1
5	50	1.033	0.3	1×10^{-4}			5.5	4	0.08
6	6	1.033	0.4	1×10^{-4}	5×10^{-6}		5.5	4	0.06
7	20	1.043	0.2	1×10^{-4}	1×10^{-5}		5.5	4	0.06
8	20	1.043	0.2	1×10^{-4}	1×10^{-5}		5.5	4	0.06
9	13	1.043	0.1	1×10^{-4}	3.5×10^{-6}		5.5	4	0.04
10	8	1.031	0.15	1×10^{-4}		0.02	7	6	0.05
11	35	1.030	7×10^{-6}	1×10^{-4}			6	5	0.06
12-18	0.1	1.066							
19	0.15 [b]	1.066							

Note: most values are the ones suggested for mesophilic solids in the ADM1 [8], except: b_j [25], θ_j [7], $K_{I,\mathrm{fe}}$ [26] and $k_{\mathrm{dis,bio}}$ [13].
[a] k_j is $k_{\mathrm{hyd},j}$ for $j \in [1,3]$, $k_{m,j}$ for $j \in [4,11]$, b_j for $j \in [12,18]$, and $k_{\mathrm{dis,bio}}$ for $j = 19$.
[b] Initial guess for parameter estimation.

The yield of product p on substrate i ($f_{p,i}$), except $f_{p,\mathrm{bio}}$, were given the values suggested in the ADM1 (not shown). The yield of product p on biomass ($f_{p,\mathrm{bio}}$) during biomass decay were assumed as follows: 0.104 carbohydrates, 0.664 protein, 0.032 lipids, 0.1 soluble inert and 0.1 suspended inert [13]. The suspended inert material was composed of particulate and colloidal material individually calculate as follow:

$$f_{\mathrm{CI,bio}} = i_{\mathrm{C,CXI,bio}}\left(1 - f_{\mathrm{SI,bio}} - f_{\mathrm{Xch,bio}} - f_{\mathrm{Xpr,bio}} - f_{\mathrm{Xli,bio}}\right) \tag{S.15}$$

$$f_{\mathrm{XI,bio}} = \left(1 - i_{\mathrm{C,CXI,bio}}\right)\left(1 - f_{\mathrm{SI,bio}} - f_{\mathrm{Xch,bio}} - f_{\mathrm{Xpr,bio}} - f_{\mathrm{Xli,bio}}\right) \tag{S.16}$$

, where $i_{\mathrm{C,CXI,bio}}$ is the colloidal fraction of the biomass suspended inert material, which was an adjustable parameter between 0 and 1, with an initial estimation of 0.1, that is: 10% of the suspended inter material release during biomass decay is colloidal and 90% is particulate.

Table S6. Nominal values and range for global sensitivity analysis (GSA) of the adjustable parameters in the biochemical flocculation **model.**

Parameter	Description	Units	Nominal value	Lower bound	Upper bound
$i_{\mathrm{C,CXI,bio}}$	Colloidal fraction of the released suspended inert material upon biomass decay	kgCOD kgCOD^{-1}	0.10	0	1
$i_{\mathrm{CI,CSInf}}$	Inert colloidal content in the submicron material of the influent	kgCOD kgCOD^{-1}	0.03	0	1
$i_{\mathrm{Saa,SInf}}$	Amino acids content in the soluble material of the influent	kgCOD kgCOD^{-1}	0.18	0	1
$i_{\mathrm{Sfa,SInf}}$	Long chain fatty acids content in the soluble material of the influent	kgCOD kgCOD^{-1}	0.20	0	1
$i_{\mathrm{SI,SInf}}$	Inter content in the soluble material of the influent	kgCOD kgCOD^{-1}	0.15	0	1
$i_{\mathrm{Ssu,SInf}}$	Monosaccharides content in the soluble material of the influent	kgCOD kgCOD^{-1}	0.10	0	1
$i_{\mathrm{Svfa,SInf}}$	VFA content in the soluble material of the influent	kgCOD kgCOD^{-1}	0.37	0	1
$i_{\mathrm{Xch,XInf}}$	Carbohydrates content in the particulate material of the influent	kgCOD kgCOD^{-1}	0.17	0	1
$i_{\mathrm{XI,XInf}}$	Inert content in the particulate material of the influent	kgCOD kgCOD^{-1}	0.19	0	1
$i_{\mathrm{Xli,XInf}}$	Lipid content in the particulate material of the influent	kgCOD kgCOD^{-1}	0.42	0	1
$i_{\mathrm{Xpr,XInf}}$	Protein content in the particulate material of the influent	kgCOD kgCOD^{-1}	0.22	0	1
k_{ads}	Pseudo-first order reaction rate coefficient for flux enhancer adsorption	d^{-1}	48	16	144
$k_{\mathrm{dis,bio}}$	First order reaction rate coefficient for biomass disintegration	d^{-1}	0.15	0.0096	4.5
k_{floc}	Empirical flocculation-deflocculation rate	d^{-1}	0.02	0.01	0.05
$k_{\mathrm{floc,fe}}$	Flux enhancer induced flocculation yield	m kgCOD^{-1} m^3	4.2×10^{-4}	1.6×10^{-5}	1.0×10^{-3}
k_{hyd}	Unique first order reaction rate coefficient for all hydrolysis processes	d^{-1}	0.50	0.0096	4.5
$K_{L,\mathrm{ads}}$	Langmuir affinity coefficient	m^3 kg^{-1}	7.6	2	1960
$q_{m,\mathrm{ads}}$	Maximum adsorption capacity corresponding to monolayer coverage	kgCOD kg^{-1}	0.45	0.032	0.45
$Y_{\mathrm{fe,C}}$	Yield of colloidal material flocculated per unit of flux enhancer adsorbed	kg kg^{-1}	363	0.3	815

Table S7. Composition matrix: nitrogen content ($i_{N,i}$), carbon content ($i_{C,i}$) and theoretical chemical oxygen demand ($i_{COD,i}$) for the component i.

		Component i	$i_{N,i}$ (kmol kgCOD^{-1})	$i_{C,i}$ (kmol kgCOD^{-1})	$i_{COD,i}$ (kgCOD kg^{-1})
1	S_{su}	Monosaccharides	0	0.0313	
2	S_{aa}	Amino Acids	0.007	0.0054 [c]	
3	S_{fa}	Long chain fatty acids (LCFA)	0	0.0217	
4	S_{va}	Total Valerate	0	0.0240	
5	S_{bu}	Total Butyrate	0	0.0250	
6	S_{pro}	Total Propionate	0	0.0268	
7	S_{ac}	Total Acetate	0	0.0313	
8	S_{h2}	Hydrogen gas	0	0	
9	S_{ch4}	Methane gas	0	0.0156	
10	S_{IC}	Inorganic Carbon	0	1	
11	S_{IN}	Inorganic Nitrogen	1	0	
12	S_I	Soluble inerts [a]	0.00625	0.0313	
13	S_{fe}	Flux enhancer in bulk liquid	0.0045 [b]	0.0357 [b]	
14	X_{ch}	Carbohydrates	0	0.0313	1.19 [d]
15	X_{pr}	Proteins	0.007	0.0054 [c]	1.42 [d]
16	X_{li}	Lipids	0	0.0220	2.90 [d]
17	X_{su}	Sugar degraders	0.00625	0.0313	1.42 [e]
18	X_{aa}	Amino acid degraders	0.00625	0.0313	1.42 [e]
19	X_{fa}	LCFA degraders	0.00625	0.0313	1.42 [e]
20	X_{c4}	Valerate and butyrate degraders	0.00625	0.0313	1.42 [e]
21	X_{pro}	Propionate degraders	0.00625	0.0313	1.42 [e]
22	X_{ac}	Acetate degraders	0.00625	0.0313	1.42 [e]
23	X_{h2}	Hydrogen degraders	0.00625	0.0313	1.42 [e]
24	X_I	Particulate inerts [a]	0.00625	0.0313	1.42 [e]
25	C_I	Colloidal Inerts [a]	0.00625	0.0313	1.42 [e]
26	X_{fe}	Flux enhancer adsorbed	0.0045 [b]	0.0357 [b]	1.14 [f]
27	X_{bio}	Decayed biomass	0.00625	0.0313	1.42 [e]

Note: unless otherwise stated the reference is the ADM1 [8].
[a] Biomass values assumed for inert material.
[b] Calculated from the chemical formula of polydiallyldimethylammonium chloride, $(C_8H_{16}NCl)_n$.
[c] Mean value of inorganic carbon content in different amino acids presented in the ADM1.
[d] Reference Lidholm and Ossiansson [27].
[e] Reference Mara et al. [2].
[f] Experimentally measured in Adifloc KD451.

The exact composition of the blackwater that was used as influent in the biochemical-flocculation model was unknown. Therefore, we estimated the composition based on the blackwater characterisation in Table S2, literature values and adjustable parameters. The total concentration of soluble ($c_{\mathrm{S,Inf}}$), colloidal ($c_{\mathrm{C,Inf}}$) and particulate ($c_{\mathrm{X,Inf}}$) materials in the influent were calculated as follows:

$$c_{\mathrm{S,Inf}} = \mathrm{csCOD_{BW}}\left(1 - i_{\mathrm{C,CSInf}}\right) \tag{S.17}$$

$$c_{\mathrm{C,Inf}} = \mathrm{csCOD_{BW}}\, i_{\mathrm{C,CSInf}} \tag{S.18}$$

$$c_{\mathrm{X,Inf}} = \mathrm{csCOD_{BW}} - \mathrm{tCOD_{BW}} \tag{S.19}$$

, where $i_{\mathrm{C,CSInf}}$ is the content of colloidal material in the submicron material of the influent, which was an adjustable parameter between 0 and 1. We assumed negligible concentrations of hydrogen, methane, FE and biomass in the influent. The concentration of each component i in the influent ($c_{i,\mathrm{Inf}}$,) was calculated as follows:

$$c_{i,\mathrm{Inf}} = \begin{cases} i_{i,\mathrm{SInf}}\, c_{\mathrm{S,Inf}} & , \quad i \in [1,2,3,4,5,6,7,12] \\ i_{i,\mathrm{XInf}}\, c_{\mathrm{X,Inf}} & , \quad i \in [14,15,16,24] \\ c_{\mathrm{C,Inf}} & , \quad i = 25 \\ \mathrm{Alk_{BW}}/_{50.044} & , \quad i = 10 \\ \mathrm{NH4_{BW}} & , \quad i = 11 \\ 0 & , \quad i \in [8,9,13,26] \\ 0 & , \quad 17 \leq i \leq 23 \text{ (biomass)} \end{cases} \tag{S.20}$$

, where $i_{i,\mathrm{SInf}}$ and $i_{i,\mathrm{XInf}}$ are the content of component i in the soluble and particulate fractions of the influent, respectively. The particulate fraction was composed of carbohydrates ($i_{\mathrm{Xch,XInf}}$), proteins ($i_{\mathrm{Xpr,XInf}}$), lipids ($i_{\mathrm{Xli,XInf}}$) and interts ($i_{\mathrm{XI,XInf}}$). The soluble fraction was composed of volatile fatty acids (VFA) ($i_{\mathrm{Svfa,SInf}}$), monosaccharides ($i_{\mathrm{Ssu,SInf}}$), amino acids ($i_{\mathrm{Saa,SInf}}$), long chain fatty acids ($i_{\mathrm{Sfa,SInf}}$) and interts ($i_{\mathrm{SI,SInf}}$). These parameters were optimised during model calibration, the initial values were taken from literature [11,16] and the range for GSA was set between 0 and 1. To reduce the amount of adjustable parameters, the total VFA content in the soluble material of the influent ($i_{\mathrm{Svfa,SInf}}$) was optimised instead of the four individual acids in the model. The content of the individual VFA was as follows:

$$i_{i,\mathrm{SInf}} = i_{\mathrm{Svfa,SInf}}\, i_{i,\mathrm{Svfa}} \tag{S.21}$$

, where $i_{i,\mathrm{Svfa}}$ is fraction of component i in the total VFA fraction; $i_{i,\mathrm{Svfa}}$ was 0.05, 0.08, 0.24, 0.63 for acetic, propionic, butyric and valeric acid, respectively [11]. For mass conservation $\sum_i i_{i,\mathrm{XInf}} = 1$ and $\sum_i i_{i,\mathrm{SInf}} = 1$, therefore, the model included the following equation to avoid values higher than 1 during model calibration:

$$i_{i,\mathrm{XInf}} = \frac{i_{i,\mathrm{XInf}}}{\sum_i i_{i,\mathrm{XInf}}} \tag{S.22}$$

$$i_{i,\mathrm{SInf}} = \frac{i_{i,\mathrm{SInf}}}{\sum_i i_{i,\mathrm{SInf}}} \tag{S.23}$$

The flocculation related parameters were all adjustable parameters because they were first introduced in this research. The nominal values and ranges used for GSA are summarised in Table S6. As explained in Odriozola et al. [17], the nominal values and range for the Langmuir adsorption parameters, $q_{m,\mathrm{ads}}$ and $K_{L,\mathrm{ads}}$, were based on previously reported values for the adsorption of polydiallyldimethylammonium chloride (polyDADMAC) onto different adsorbents [3,4,18].

The nominal value of the empirical flocculation coefficient (k_{floc}) was the inverse of the 50 days needed for d_p to return to $d_{p,\text{St}}$ in the pilot AnMBR after being spiked with FE. The range was the inverse of the period when d_p approximates to $d_{p,\text{St}}$ after the spike, which was between 20 and 100 days. The nominal value of the FE adsorption rate coefficient (k_{ads}) was 48 d^{-1}, and the range was between 16 and 144 d^{-1}, estimated with the batch flocculation kinetic experiments described in Section S2. The yield of inert colloidal material flocculated per unit of FE adsorbed onto the particulate material ($Y_{\text{fe,C}}$) and the FE induced flocculation coefficient ($k_{\text{floc,fe}}$) were calculated based on the FE dosage-step experiments described in Section S1 as follows:

$$Y_{\text{fe,C}} = \frac{\text{csCOD} - \text{csCOD}_0}{c_{\text{fe}}} \frac{\text{TSS}}{\text{csCOD}} \quad (S.24)$$

$$k_{\text{floc,fe}} = \frac{d_p - d_{p0}}{c_{\text{fe}}} \quad (S.25)$$

, where csCOD_0 and d_{p0} are the csCOD and d_p without FE (at the beginning of the experiment), respectively. $Y_{\text{fe,C}}$ and $k_{\text{floc,fe}}$ were calculated for each test and at each FE dosage; the mean, minimum and maximum values were used as nominal, lower and upper bounds, respectively.

AnMBR and AnDFCm filtration models

The intrinsic resistance of the membrane in the AnDFCm installation was 5×10^{11} m^{-1} which was the resistance when filtering water after performing chemical cleaning. This value was assumed as the R_{m} for both the AnMBR and the AnDFCm.

The nominal values for the adjustable parameters in the filtration models are summarised in Table S8. Most parameters were taken from literature, and $K_{S,\text{G}}$, γ_3, and k_{CK} were estimated based on experimental data and different assumptions.

Table S8. Nominal values of the adjustable parameters in the AnMBR and AnDFCm filtration models.

Parameter	Description	Units	Values	Reference
Deposition parameters				
C_d	Drag coefficient	-	0.40	[28]
$f_{\mathrm{C,c}}$	Fraction of colloidal material deposited onto the membrane	-	0.25	[28]
$f_{\mathrm{X,c}}$	Fraction of particulate material deposited onto the membrane	-	0.25	This research [a]
K_F	Parameter representing the fouling rate when J_{20} tends to zero	Pa s^{-1}	5.6×10^{-4}	[29]
$K_{S,\mathrm{c}}$	Half-saturation coefficient for cake mass during membrane scouring	kg	0.2	[29]
$q_{m,\mathrm{MS}}$	Maximum membrane scouring velocity	-	4.71	[30]
β_{ST}	Lumped parameter $\beta_{\mathrm{ST}} = \beta(1 - K_{\mathrm{ST}})$; β Erosion rate coefficient of the sludge cake; K_{ST} stickiness coefficient	-	1.75×10^{-4}	[31]
γ	Compression coefficient for the dynamic cake layer	kg m^{-3}	2.0×10^{-5}	[31]
γ_0	Empirical model parameter	s m^{-1}	2.81×10^{6}	[29]
γ_1	Empirical model parameter	s^2 m^{-1}	2.48×10^{8}	[29]
γ_2	Empirical model parameter	s m^{-2} kg^{-1}	5.1×10^{4}	[29]
γ_3	Empirical model parameter	s m^{-2} kg^{-1}	1.28×10^{6}	This research [b]
γ_4	Empirical model parameter	s m^{-2}	1.75×10^{10}	This research [b]
γ_G	Empirical model parameter	-	2.15	This research [c]
Specific cake resistance parameters				
k_c	Cake resistance coefficient	m^{-2}	1.0×10^{17}	[31]
k_{CK}	Carman-Kozeny cake resistance coefficient	-	4.0×10^{7}	This research [d]
P_{a}	Pressure needed to double the specific resistance	Pa	2.01×10^{4}	[30]
P_{b}	Transmembrane pressure coefficient	Pa	4179.9	[32]
$\varepsilon_{\mathrm{c0}}$	Cake layer porosity without colloidal material	-	0.66	[28]
ζ_1	Empirical model parameters	-	1.16×10^{3}	[33]
ζ_2	Empirical model parameters	-	1.36×10^{4}	[33]
ζ_3	Empirical model parameters	-	172.4	[33]
ζ_4	Empirical model parameters	-	150.9	[33]
ρ_{C}	Density of colloidal material	kg m^{-3}	4.98×10^{3}	[28]
ρ_{X}	Density of particulate material	kg m^{-3}	1.24×10^{3}	[28]

[a] Assumed equal to $f_{\mathrm{C,c}}$.

[b] Estimated to achieve similar weights to particulate matter on FR as follow: γ_3=γ_2 $\bar{c_{\mathrm{X}}}/\bar{c_{\mathrm{C}}}$ and γ_4=γ_2 $\bar{c_{\mathrm{X}}}/\overline{d_p}$, where $\bar{c_{\mathrm{X}}}$, $\bar{c_{\mathrm{C}}}$ and $\overline{d_p}$ are the mean values in the pilot AnMBR for particulate material, colloidal material and floc size, respectively.

[c] Calculated with the experimental representative data iD6 and iD7 from the pilot AnMBR as follows: $\gamma_G = -(\log(\overline{FR}|_{\mathrm{iD6}}) - \log(\overline{FR}|_{\mathrm{iD7}}))/(\log(\overline{u_{\mathrm{G}}}|_{\mathrm{iD6}}) - \log(\overline{u_{\mathrm{G}}}|_{\mathrm{iD7}}))$, where $\overline{FR}|_{\mathrm{iD6}}$ and $\overline{FR}|_{\mathrm{iD7}}$ are the mean fouling rate and $\overline{u_{\mathrm{G}}}|_{\mathrm{iD6}}$ and $\overline{u_{\mathrm{G}}}|_{\mathrm{iD7}}$ the mean superficial gas velocity in the datasets iD6 and iD7, respectively.

[d] Estimated to obtain $\alpha_{c,1} \approx \alpha_{c,2}$ as follows: k_{CK}=$k_c d_p^2$, assuming floc size of 2×10^{-5} m.

S5. Model calibration procedure

The calibration procedure was as follows: first, the subset containing only influential parameters (θ_I) was selected using global sensitivity analysis (GSA). The GSA was based on linear regression models built from Monte Carlo simulations using Latin hypercube sampling with N = 500, where N was selected by convergence analysis.

The convergence analysis, for N determination, was based on the stability in parameter selection. The convergence analysis was as follows [19]: 30 batches (k=30) of Monte Carlo simulations using Latin hypercube sampling with 100 samples (n=100) per batch were performed. The SRC were calculated after each batch with the cumulative number of output files, where $\beta_{i,k}$ was the SRC of the i-th parameter in the k-th batch. The convergence criterion was the stability in the parameter selection, with N=100k when the parameters with $|\beta_{i,k}| \geq 0.1$ in the k-th batch remain the same for five consecutive batches.

The input uncertainty was uniform. In the filtration modes, the variability was set to 20% around the initial or nominal parameter vector ($\theta°$), this is $\theta \sim U(0.8\theta°, 1.2\theta°)$. In the biochemical-flocculation model the upper and lower limits for GSA were selected from literature or proposed based on experimental observations.

The standardised regression coefficients (SRC, β_k) for each parameter k were computed using the objective function ($f_{\text{objective}}$) in Equation (S.26) as output. When $R^2 \geq 0.7$, we selected influential parameters with $|\beta_k| \geq 0.1$ [20].

$$f_{\text{objective}} = \sqrt{\sum_j w_j \frac{\sum_i \left(y_{e,j,i} - y_{m,j,i}\right)^2}{n_j}} \tag{S.26}$$

, where w_j is the weight, $y_{e,j,i}$ the i-th experimental value, $y_{m,j,i}$ the i-th predicted value, and n_j the number of experimental observations of the output variable j. For a unique output variable, $w_j = 1$ and the objective function becomes the root-mean-square error (RMSE). For multiple output variables, $f_{\text{objective}}$ was calculated using normalised values by defining w_j as the inverse of the mean experimental value as follows: $w_j = \left(\sum_i y_{e,j,i}/n_j\right)^{-1}$.

Alternatively, if $R^2 < 0.7$, we computed individual $\beta_k(t)$ using the predicted values $y(t)$ as model output for each operational time; then calculated the mean $\overline{\beta_k}$ using the $\beta_k(t)$ values when $R^2 \geq 0.7$; then selected the influential parameters with $|\overline{\beta_k}| \geq 0.1$.

Subsequently, the parameters in θ_I were estimated using the nominal parameter values as initial guess ($\theta°$), and by minimising $f_{\text{objective}}$. Initially, an identifiability analysis from θ_I was used to select a new subset θ_{II} that can be reliably estimated from the given experimental data; and the parameters in θ_{II} were estimated. Then, θ_{III} was defined with the parameters contained in θ_I and not in θ_{II}, and the parameters in θ_{III} were estimated. The quality of the estimators $\hat{\theta}$ was evaluated based on the relative error ($\sigma_\theta/\hat{\theta}$) as follows: below 0.1 good, above 0.5 poor [20], and between 0.1 and 0.5 moderate.

The identifiability analysis was performed by computing the collinearity index of the parameter subset k (γ_k) [20]. We selected the parameter subset with γ_k below 10 which contained the most influential parameter and had the highest number of parameters.

S6. Statistical indicators representing model accuracy

The following statistical indicators were used to estimate model accuracy during validation: normalised root mean square error (nRMSE), Pearson correlation coefficient (Corr), and mean absolute percentage error (MAPE). The correlation coefficient was calculated with the function *corr* in Matlab®, and nRMSE and MAPE where calculated as follows:

$$\mathrm{nRMSE} = \frac{\sqrt{\sum_i \left(y_{e,i} - y_{m,i}\right)^2 / n}}{\sum_i y_{e,i} / n} \tag{S.27}$$

$$\mathrm{MAPE} = \frac{100}{n} \sum_i \frac{\left|y_{e,i} - y_{m,i}\right|}{y_{e,i}} \tag{S.28}$$

, where $y_{e,i}$ is the i-th experimental value, $y_{m,i}$ the i-th predicted value, and n the number of experimental observations.

S7. Simulated influent characteristics and applied disturbances

The total and submicron blackwater COD input was generated with the "uniform random number" block from Simulink®, with the minimum and maximum values from Table S2, seed of 30 days (the pattern is repeated every 30 days) and sample time of 12 min (changes every 12 minutes). The simulated input is displayed in Figure S7.

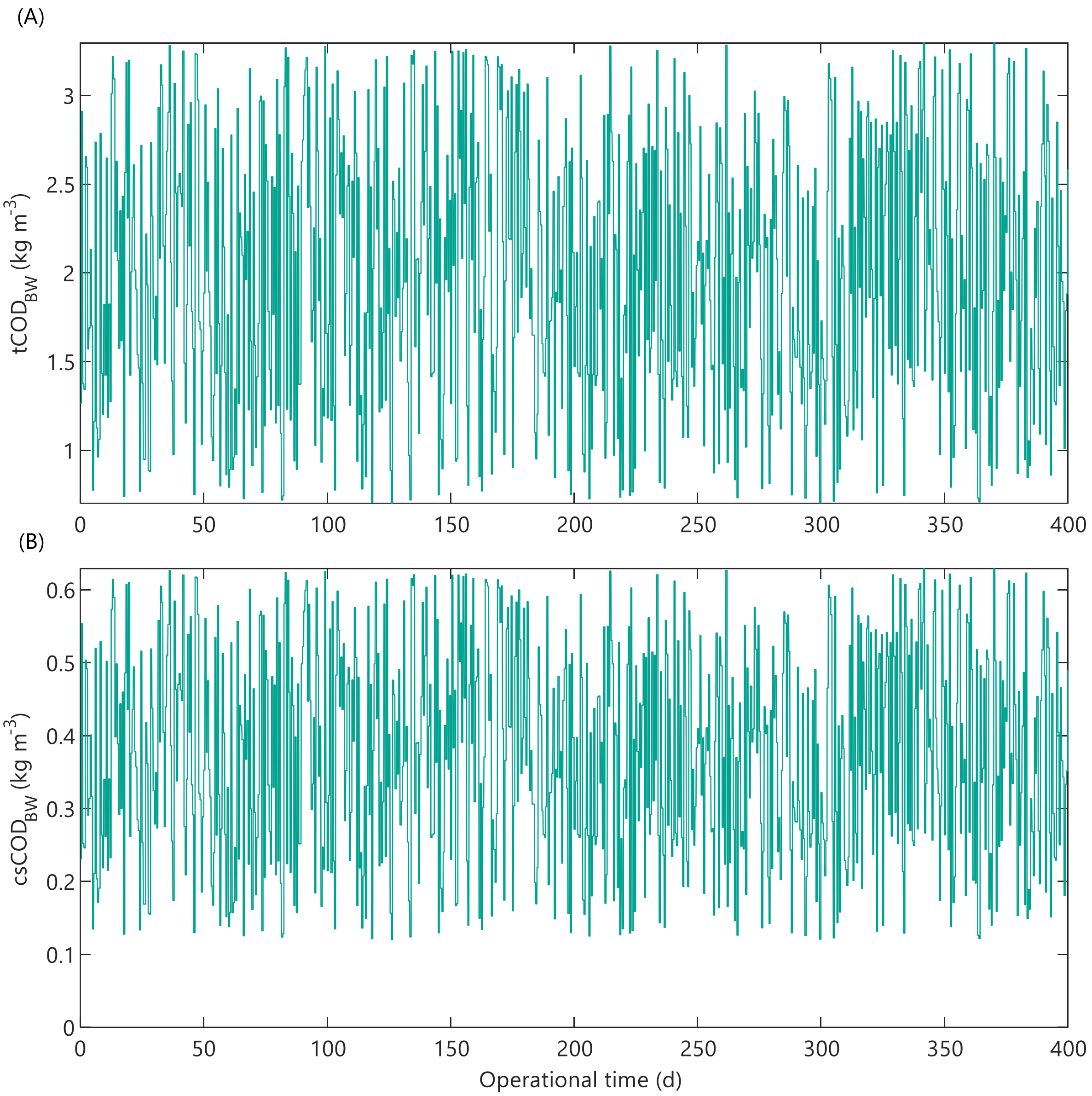

Figure S7. Simulated blackwater characteristics used in the simulation environment: (A) total COD, and (B) submicron COD.

S8. Calibration and validation of biochemical-flocculation model

Fast processes calibration results and discussion

The influential parameters, selected with GSA, in the subset $\theta = \{q_{m,\text{ads}}, K_{L,\text{ads}}, k_{\text{ads}}, Y_{\text{fe,C}}, k_{\text{floc,fe}}\}$ were estimated to fit the experimental csCOD and d_p measured in the dosage-step experiments using sludge samples from a reactor fed with blackwater.

The linear models built using $f_{\text{objective}}$ had R^2 of 0.63 and 0.06 for the variables csCOD and d_p, respectively; and R^2 was 0.52 when both csCOD and d_p were used together in $f_{\text{objective}}$. Consequently, because R^2 were below 0.7, these models built with $f_{\text{objective}}$ were not used for sensitivity evaluation. Instead, as described in Section S5, the sensitivity was evaluated with the mean SRC ($\overline{\beta_k}$) of the linear models that had R^2 > 0.7, the models were built with the predicted csCOD or d_p at each operational time instant.

The mean R^2 were 0.90 and 0.98 for csCOD and d_p, respectively. Results in Table S9 show that $Y_{\text{fe,C}}$ was the only influential parameter (with $|\overline{\beta_k}| \geq 0.1$) on csCOD, and $k_{\text{floc,fe}}$ on d_p. Therefore, the parameters were estimated separately for each output variable as follows: $k_{\text{floc,fe}}$ was estimated to fit the experimental d_p, and $Y_{\text{fe,C}}$ to fit the experimental csCOD. Results in Table S9 show that the optimal values ($\hat{\theta}$) had good quality because the relative error, $\sigma_\theta/\hat{\theta}$, was below 0.1 [20]. Figure S8 compares the experimental observations with the simulated values for the calibrated parameters. The model satisfactorily predicted the csCOD in the batch tests; however, the model overestimated or underestimated the floc size for a considerable number of observations. Therefore, the model in Equation (10) could be further developed to improve the prediction of floc size changes caused by FE dosing.

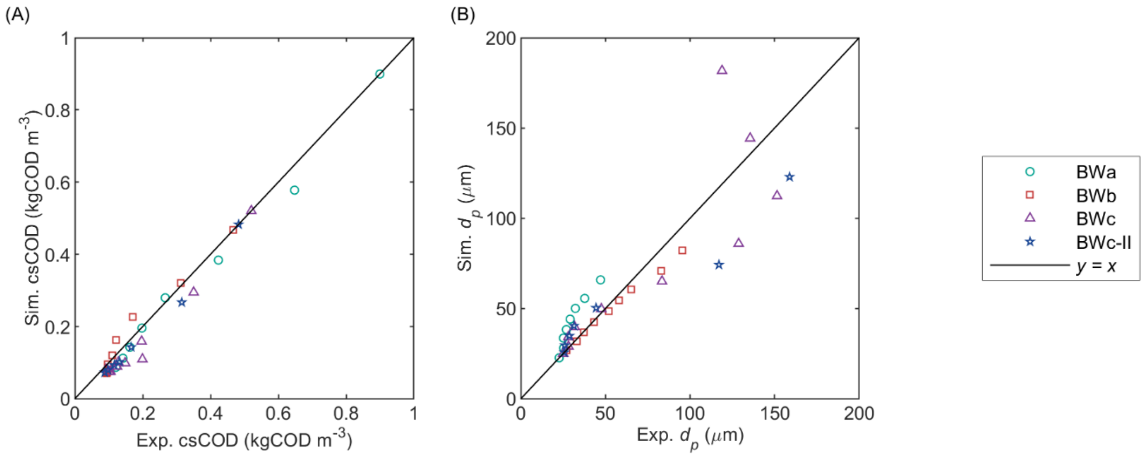

Figure S8. Short-term biochemical-flocculation model calibration. Sludge characteristics during flux enhancer dosage-step test with sludge samples from a pilot AnMBR fed with blackwater: (**A**) submicron COD, and (**B**) mean geometric particle diameter.

Table S9. Flocculation model calibration results: mean of the standardised regression coefficients with R^2>0.7 ($\overline{\beta_k}$) for different output variables, and values ($\hat{\theta}$), standard deviation (σ_θ), and relative error ($\sigma_\theta/\hat{\theta}$) of the estimated parameters.

Parameter	Units	$\overline{\beta_k}$ for csCOD	$\overline{\beta_k}$ for d_p	$\hat{\theta}$	σ_θ	$\sigma_\theta/\hat{\theta}$
k_{ads}	d^{-1}	-0.07	0.07			
$k_{\text{floc,fe}}$	m kgCOD^{-1} m^3	-0.02	0.98	6.00×10^{-4}	0.43×10^{-4}	0.07
$K_{L,\text{ads}}$	m^3 kg^{-1}	-0.03	0.003			
$q_{m,\text{ads}}$	kgCOD kg^{-1}	-0.03	0.02			
$Y_{\text{fe,C}}$	kg kg^{-1}	-0.95	0.0004	649.8	0.34	0.001

Slow processes calibration results and discussion

Table S10 summarises the SRC of the linear models built using $f_{\text{objective}}$ with different output variables. The linear model with TSS had an R^2 below 0.7, and thus the sensitivity on TSS was evaluated with the $\overline{\beta_k}$ of the linear models that had R^2 > 0.7.

Based on the GSA results, k_{floc} was estimated with d_p as a unique output variable because k_{floc} was the only influential parameter on d_p and had a negligible influence on TSS and cCOD. Then, $i_{\text{CI,CSInf}}$ and $i_{\text{Xli,XInf}}$ were estimated with cCOD and TSS as output variables because they were the only influential parameter on cCOD and the most influential parameters on TSS. The subset $\{i_{\text{Xch,XInf}}, i_{\text{Xli,XInf}}, i_{\text{Xpr,XInf}}\}$ was also influential on TSS, however, the model did not improve further by optimising this subset with TSS as output variable, the optimal values were almost identical to the initial guess, thus, the initial (nominal) values were used for $i_{\text{Xch,XInf}}$, $i_{\text{Xli,XInf}}$ and $i_{\text{Xpr,XInf}}$. Table S11 summarises the estimated parameters. The parameters $i_{\text{CI,CSInf}}$ and $i_{\text{Xli,XInf}}$ had good quality, whereas k_{floc} had medium quality.

Table S10. Sensitivity analysis of the biochemical-flocculation model: standardised regression coefficients (β_k) and mean of the standardised regression coefficients with R^2>0.7 ($\overline{\beta_k}$) for different output variables. Influential parameters with absolute value above 0.1 (*).

Parameter	Units	β_k for TSS, cCOD & d_p	β_k for TSS & cCOD	β_k for TSS	β_k for d_p	β_k for cCOD	$\overline{\beta_k}$ for TSS
	R^2 →	0.97	0.97	0.45	0.95	0.97	$\overline{R^2}$=0.77
$i_{\mathrm{C,CXI,bio}}$	kgCOD kgCOD^{-1}	0.05	0.05	-0.01	-0.01	0.05	-0.01
$i_{\mathrm{CI,CSInf}}$	kgCOD kgCOD^{-1}	0.96*	0.96*	0.32*	0.06	0.95*	0.46*
$i_{\mathrm{Saa,SInf}}$	kgCOD kgCOD^{-1}	0.01	0.01	-0.05	-0.01	0.01	-0.02
$i_{\mathrm{Sfa,SInf}}$	kgCOD kgCOD^{-1}	0.01	0.01	0.03	0.01	0.01	0.01
$i_{\mathrm{SI,SInf}}$	kgCOD kgCOD^{-1}	-0.001	-0.001	0.002	-0.01	-0.001	-0.01
$i_{\mathrm{Ssu,SInf}}$	kgCOD kgCOD^{-1}	-0.01	-0.01	0.01	-0.01	-0.01	-0.003
$i_{\mathrm{Svfa,SInf}}$	kgCOD kgCOD^{-1}	-0.001	-0.001	-0.01	0.02	-0.0003	0.001
$i_{\mathrm{Xch,XInf}}$	kgCOD kgCOD^{-1}	0.04	0.04	-0.25*	-0.05	0.06	-0.25*
$i_{\mathrm{XI,XInf}}$	kgCOD kgCOD^{-1}	-0.17*	-0.17*	0.44*	0.09	-0.20*	0.62*
$i_{\mathrm{Xli,XInf}}$	kgCOD kgCOD^{-1}	0.04	0.04	-0.22*	-0.05	0.06	-0.24*
$i_{\mathrm{Xpr,XInf}}$	kgCOD kgCOD^{-1}	0.04	0.04	-0.15*	-0.02	0.05	-0.21*
$k_{\mathrm{dis,bio}}$	d^{-1}	0.001	0.001	-0.01	-0.01	0.002	-0.003
k_{floc}	d^{-1}	-0.02	-0.01	0.00	-0.95*	-0.01	0.004
k_{hyd}	d^{-1}	0.002	0.002	-0.11*	-0.01	0.01	-0.05

Table S11. Parameter estimation of the biochemical-flocculation model: values ($\hat{\theta}$), standard deviation (σ_θ), and relative error ($\sigma_\theta/\hat{\theta}$) of the estimated parameters, and output variable used for parameter estimation.

Parameter	Units	$\hat{\theta}$	σ_θ	$\sigma_\theta/\hat{\theta}$	Output variable
$i_{\mathrm{CI,CSInf}}$	kgCOD kgCOD^{-1}	0.0287	6×10^{-6}	2×10^{-4}	cCOD & TSS
$i_{\mathrm{XI,XInf}}$	kgCOD kgCOD^{-1}	0.189	9×10^{-6}	5×10^{-5}	cCOD & TSS
k_{floc}	d^{-1}	0.16	0.02	0.128	d_p

Prediction with calibrated model

Table S12. Statistical indicators representing model accuracy of the original and modified biochemical-flocculation models for long-term fouling rate prediction in the pilot AnMBR. Normalised root mean square error (nRMSE), Pearson correlation coefficient (Corr), and mean absolute percentage error (MAPE).

	nRMSE		Corr		MAPE	
Model[a] → / Output variable ↓	Original	Modified	Original	Modified	Original	Modified
d_{p}	0.10	0.06	0.94	0.96	7.1	4.2
TSS	0.13	0.13	0.90	0.90	11.3	11.3
cCOD	0.19	0.19	0.95	0.95	21.7	21.7

[a] Biochemical flocculation model using Equation (10) (Original) and Equation (44) (Modified) to describe particle size dynamics.

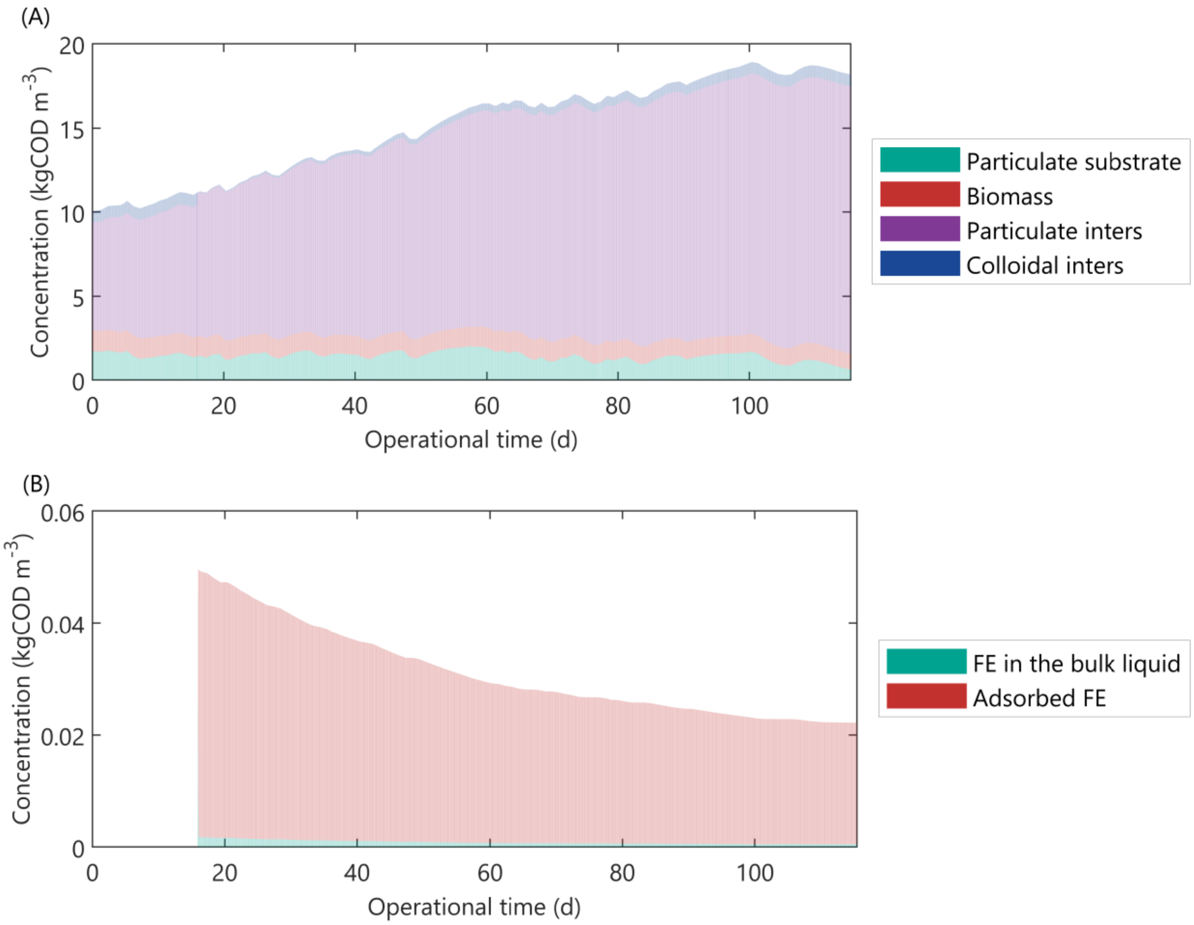

Figure S9. Simulated concentrations inside the reactor during operational period of pilot AnMBR dosed with flux enhancer (FE) on day 16: (A) particulate and colloidal materials, and (B) FE components.

S9. Calibration and validation of alternate AnMBR filtration models

Model calibration discussion

The GSA results, in Table S13, showed that almost all parameters, except γ_G, were influential in the alternate empirical FR models and were therefore optimised. The parameters were estimated using the representative dataset from the pilot AnMBR, the optimal values and quality of the estimators are summarised in Table S16. Figure S10 compares the experimental FR in the representative dataset with the predicted values using the optimised parameters for each empirical FR model. The calibrated models were unable to predict accurately each specific FR values, nevertheless, the FR trend was well predicted.

In Table S16, most estimators presented good ($\sigma_\theta/\hat{\theta} \leq 0.1$) or moderate ($0.1 < \sigma_\theta/\hat{\theta} \leq 0.5$) quality, except γ_2 which had poor quality ($\sigma_\theta/\hat{\theta} > 0.5$). The parameter γ_2 represents the influence of c_{X} on FR, and thus the high relative error of γ_2 (5.93 for FR1 and 0.60 for FR4) agreed with previous research that reported that the concentration of particulate material is a poor indicator of biomass fouling propensity by itself [21].

Table S14 summarises the GSA results for the alternate RIS_FR AnMBR filtration models. The parameters C_d, β_{ST}, k_c, k_{CK}, $\varepsilon_{\mathrm{c0}}$, ρ_{X}, and P_{b} were influential in all models, and γ_0, ζ_2 and ζ_3 were influential in most models; whereas $f_{\mathrm{C,c}}$, γ, K_F, γ_3, and ρ_{C} were none-influential in all models, and $K_{S,\mathrm{c}}$, $q_{m,\mathrm{MS}}$, γ_1, γ_2, P_{a}, ζ_1, and ζ_4 were only influential in a few models. Particularly, $\varepsilon_{\mathrm{c0}}$ was the most influential parameter in all the models that included it, presenting SRC between 0.77 and 0.93.

The parameters were estimated using the representative dataset from the pilot AnMBR. The optimal values and quality of the estimators are summarised in Table S17. Figure S11 to Figure S14 compare the experimental FR with the predicted values using the optimised parameters for each model.

The majority of the models that included a SCR submodel based on the empirical equation by Cho et al. [22], namely $\alpha_{\mathrm{c},3}$, $\alpha_{\mathrm{c},3p}$ and $\alpha_{\mathrm{c},4p}$, had at least one estimator with poor quality ($\sigma_\theta/\hat{\theta} > 0.50$). Additionally, these models were unable to predict the experimental FR in the representative dataset used for calibration. The models D1a $\alpha_{\mathrm{c},3p}$ and D1c $\alpha_{\mathrm{c},4}$ considerably underpredicted the FR when c_{C} was high (iD1 and iD2). The models D1b $\alpha_{\mathrm{c},3p}$ and D1c $\alpha_{\mathrm{c},3p}$ predicted FR well at high c_{C} but underpredicted the FR when c_{C} was low (iD4 to iD8). The remaining models had considerably poor FR predictions for all the representative datasets. Therefore, the models that included $\alpha_{\mathrm{c},3}$, $\alpha_{\mathrm{c},3p}$ or $\alpha_{\mathrm{c},4}$ could not be satisfactorily calibrated with the procedure described in Section S5.

Conversely, all the models that included a SCR submodel that is based on the Carman-Kozeny equation (i.e., $\alpha_{\mathrm{c},1}$, $\alpha_{\mathrm{c},1p}$, $\alpha_{\mathrm{c},2}$ and $\alpha_{\mathrm{c},2p}$) presented good estimators' quality ($\sigma_\theta/\hat{\theta} \leq 0.1$) and FR at high and low c_{C} in the representative dataset was well predicted. However, all these models considerably underpredicted FR when the reactor operated at low u_{G} (dataset iD6); and most models, except D1c $\alpha_{\mathrm{c},1}$ and D1c $\alpha_{\mathrm{c},2}$, slightly overpredicted FR when c_{X} was high (dataset iD3).

Global sensitivity analysis results

Table S13. Sensitivity analysis of AnMBR empirical fouling rate (FR) models: standardised regression coefficients (β_k) and mean of the standardised regression coefficients with R^2>0.7 ($\overline{\beta_k}$). Influential parameters with absolute β_k or $\overline{\beta_k}$ value above 0.10 (*).

Model	R^2	$\overline{R^2}$	β_k ($\overline{\beta_k}$)						
			K_F	γ_0	γ_1	γ_2	γ_3	γ_4	γ_G
FR1	0.13	0.81	0.05 (0.12*)	-0.28* (0.86*)	-0.10 (-0.14*)	0.15* (0.19*)	0.09 (0.15*)		
FR2	0.85	0.83	-0.13*	-0.88*	0.12*		-0.17*		
FR3	0.02	0.85	0.10 (0.15*)	0.04 (0.90*)			0.08 (0.13*)		0.07 (0.06)
FR4	0.88	0.81	-0.12*	-0.90*	0.11*	-0.13*	-0.14*	0.13*	
FR5	0.82	0.82	-0.15*	-0.84*	0.11*		-0.18*	0.16*	
FR6	0.88	0.83	-0.11*	-0.91*			-0.15*	0.17*	-0.10

Table S14. Sensitivity analysis of AnMBR filtration models: standardised regression coefficients (β_k) and mean of the standardised regression coefficients with R^2>0.7 ($\overline{\beta_k}$). Influential parameters with absolute β_k or $\overline{\beta_k}$ value above 0.10 (*).

Model	R^2	$\overline{R^2}$	β_k ($\overline{\beta_k}$)					
			C_d	$f_{\mathrm{C,c}}$	$K_{S,\mathrm{c}}$	$q_{m,\mathrm{MS}}$	β_{ST}	γ
D1a $\alpha_{\mathrm{c},1}$	0.92	0.89			-0.01	0.02		
D1a $\alpha_{\mathrm{c},1p}$	0.03	0.80			-0.02 (0.001)	-0.09(-0.002)		
D1a $\alpha_{\mathrm{c},2}$	0.95	0.89			-0.01	-0.004		
D1a $\alpha_{\mathrm{c},2p}$	0.07	0.83			-0.02 (-0.03)	-0.09 (-0.02)		
D1a $\alpha_{\mathrm{c},3}$	0.98	0.92			0.003	0.001		
D1a $\alpha_{\mathrm{c},3p}$	0.96	0.88			-0.06	0.08		
D1a $\alpha_{\mathrm{c},4p}$	0.05	0.79			0.11* (0.06)	-0.03 (-0.05)		
D1b $\alpha_{\mathrm{c},1}$	0.94	0.91			-0.004	-0.01		
D1b $\alpha_{\mathrm{c},1p}$	0.04	0.79			0.04 (0.02)	0.05 (-0.03)		
D1b $\alpha_{\mathrm{c},2}$	0.97	0.91			0.01	-0.002		
D1b $\alpha_{\mathrm{c},2p}$	0.03	0.84			-0.02(-0.001)	-0.02 (-0.03)		
D1b $\alpha_{\mathrm{c},3}$	0.97	0.91			-0.01	0.01		
D1b $\alpha_{\mathrm{c},3p}$	0.96	0.88			-0.08	0.12*		
D1b $\alpha_{\mathrm{c},4p}$	0.03	0.87			-0.04 (0.04)	-0.10 (-0.08)		
D1c $\alpha_{\mathrm{c},1}$	0.93	0.92			-0.02	-0.01		
D1c $\alpha_{\mathrm{c},1p}$	0.84	0.85			0.01	0.03		
D1c $\alpha_{\mathrm{c},2}$	0.94	0.93			-0.02	0.02		
D1c $\alpha_{\mathrm{c},2p}$	0.74	0.73			-0.01	0.02		
D1c $\alpha_{\mathrm{c},3}$	0.99	0.92			-0.04	0.06		
D1c $\alpha_{\mathrm{c},3p}$	0.98	0.91			-0.07	0.10*		
D1c $\alpha_{\mathrm{c},4p}$	0.92	0.86			-0.27*	0.37*		
D2 $\alpha_{\mathrm{c},1}$	0.90	0.90	0.14*	-0.03			0.21*	0.01
D2 $\alpha_{\mathrm{c},1p}$	0.91	0.90	0.16*	0.01			0.20*	0.02
D2 $\alpha_{\mathrm{c},2}$	0.90	0.90	0.16*	-0.04			0.21*	-0.005
D2 $\alpha_{\mathrm{c},2p}$	0.89	0.89	0.14*	-0.02			0.17*	-0.01
D2 $\alpha_{\mathrm{c},3}$	0.98	0.97	0.39*	-0.07			0.56*	-0.01
D2 $\alpha_{\mathrm{c},3p}$	0.98	0.97	0.42*	-0.05			0.59*	0.003
D2 $\alpha_{\mathrm{c},4p}$	0.98	0.97	0.47*	-0.08			0.67*	-0.004

(continued)

Table S14 continued. Sensitivity analysis of AnMBR filtration models: standardised regression coefficients (β_k) and mean of the standardised regression coefficients with R^2>0.7 ($\overline{\beta_k}$). Influential parameters with absolute β_k or $\overline{\beta_k}$ value above 0.10 (*).

Model	β_k ($\overline{\beta_k}$)				
	K_F	γ_0	γ_1	γ_2	γ_3
D1a $\alpha_{c,1}$	-0.02	-0.32*	0.04	-0.03	
D1a $\alpha_{c,1p}$	0.02 (0.04)	-0.004 (0.28*)	-0.01 (-0.07)	0.03 (0.05)	
D1a $\alpha_{c,2}$	-0.04	-0.31*	0.03	-0.03	
D1a $\alpha_{c,2p}$	0.04 (0.03)	-0.02 (0.02)	-0.004 (-0.02)	0.06 (0.02)	
D1a $\alpha_{c,3}$	-0.09	-0.78*	0.11*	-0.11*	
D1a $\alpha_{c,3p}$	-0.10	-0.69*	0.10*	-0.09	
D1a $\alpha_{c,4p}$	-0.06 (0.05)	0.11* (0.48*)	-0.02 (-0.08)	0.11* (0.05)	
D1b $\alpha_{c,1}$	-0.03	-0.16*	0.02	-0.02	-0.03
D1b $\alpha_{c,1p}$	0.04 (0.02)	0.05 (0.23*)	-0.01 (-0.03)	0.04 (0.02)	0.06 (0.01)
D1b $\alpha_{c,2}$	-0.01	-0.14*	0.02	-0.03	-0.03
D1b $\alpha_{c,2p}$	0.06 (0.01)	0.01 (0.01)	-0.06 (-0.02)	-0.01 (-0.01)	-0.03 (0.005)
D1b $\alpha_{c,3}$	-0.07	-0.49*	0.09	-0.07	-0.10
D1b $\alpha_{c,3p}$	-0.03	-0.40*	0.07	-0.06	-0.08
D1b $\alpha_{c,4p}$	0.05 (0.07)	0.06 (0.51*)	-0.03 (-0.03)	-0.06 (0.05)	-0.02 (0.03)

(continued)

Table S14 continued. Sensitivity analysis of AnMBR filtration models: standardised regression coefficients (β_k) and mean of the standardised regression coefficients with R^2>0.7 ($\overline{\beta_k}$). Influential parameters with absolute β_k or $\overline{\beta_k}$ value above 0.10 (*).

Model	β_k ($\overline{\beta_k}$)					
	k_c	k_{CK}	ε_{c0}	ρ_C	ρ_X	P_a
D1a $\alpha_{c,1}$		-0.18*	0.85*	0.003	0.18*	
D1a $\alpha_{c,1p}$		0.04 (0.18*)	-0.07 (-0.77*)	-0.04 (0.01)	-0.08 (-0.18*)	-0.08 (-0.04)
D1a $\alpha_{c,2}$	-0.18*		0.89*	0.01	0.17*	
D1a $\alpha_{c,2p}$	0.09 (0.22*)		-0.2* (-0.86*)	0.005 (-0.03)	-0.1 (-0.21*)	-0.06 (-0.07)
D1b $\alpha_{c,1}$		-0.19*	0.91*	-0.001	0.18*	
D1b $\alpha_{c,1p}$		0.08 (0.2*)	-0.09 (-0.81*)	0.05 (0.02)	-0.05 (-0.18*)	-0.06 (-0.07)
D1b $\alpha_{c,2}$	-0.19*		0.93*	0.01	0.17*	
D1b $\alpha_{c,2p}$	-0.03 (0.2*)		-0.09 (-0.86*)	0.0004 (-0.03)	-0.08 (-0.18*)	-0.08 (-0.06)
D1c $\alpha_{c,1}$		-0.18*	0.91*	0.03	0.21*	
D1c $\alpha_{c,1p}$		-0.19*	0.87*	-0.01	0.17*	0.05
D1c $\alpha_{c,2}$	-0.20*		0.93*	0.01	0.19*	
D1c $\alpha_{c,2p}$	-0.18*		0.81*	0.02	0.19*	0.08
D2 $\alpha_{c,1}$		-0.17*	0.86*	0.02	0.16*	
D2 $\alpha_{c,1p}$		-0.17*	0.88*	0.04	0.17*	0.02
D2 $\alpha_{c,2}$	-0.16*		0.89*	0.03	0.17*	
D2 $\alpha_{c,2p}$	-0.18*		0.88*	-0.01	0.17*	-0.01

(continued)

Table S14 continued. Sensitivity analysis of AnMBR filtration models: standardised regression coefficients (β_k) and mean of the standardised regression coefficients with R^2>0.7 ($\overline{\beta_k}$). Influential parameters with absolute β_k or $\overline{\beta_k}$ value above 0.10 (*).

Model	β_k ($\overline{\beta_k}$)					
	P_a	P_b	ζ_1	ζ_2	ζ_3	ζ_4
D1a $\alpha_{c,3}$		-0.43*	-0.07	-0.37*	-0.19*	0.04
D1a $\alpha_{c,3p}$	0.14*	-0.42*	-0.05	-0.38*	-0.15*	0.04
D1a $\alpha_{c,4p}$			0.02 (0.24*)	-0.03 (0.1)	-0.06 (0.57*)	-0.05 (-0.20*)
D1b $\alpha_{c,3}$		-0.64*	-0.08	-0.54*	-0.26*	0.04
D1b $\alpha_{c,3p}$	0.20*	-0.59*	-0.06	-0.52*	-0.2*	0.04
D1b $\alpha_{c,4p}$			-0.05 (0.49*)	0.05 (0.12*)	0.03 (0.35*)	0.06 (-0.13*)
D1c $\alpha_{c,3}$		-0.70*	-0.10*	-0.61*	-0.28*	0.06
D1c $\alpha_{c,3p}$	0.12*	-0.68*	-0.09	-0.60*	-0.25*	0.05
D1c $\alpha_{c,4p}$			-0.05	-0.81*	-0.31*	0.05
D2 $\alpha_{c,3}$		-0.48*	-0.04	-0.44*	-0.06	0.01
D2 $\alpha_{c,3p}$	-0.001	-0.50*	-0.04	-0.47*	-0.08	0.01
D2 $\alpha_{c,4p}$			-0.04	-0.52*	-0.08	0.01

Parameter estimation results

Table S15. Parameter estimation procedure for the alternate AnMBR filtration models. Parameters contained in subset θ_{II} and θ_{II}, number of pairwise correlations above 0.50 (nCorr) and root mean square error (RMSE) at optimal parameter values.

Model	Subset θ_{II}			Subset θ_{III}		
	Parameters	nCorr	RMSE	Parameters	nCorr	RMSE
D1a $\alpha_{c,1}$	$\{\varepsilon_{c0}, \gamma_0\}$	0	10.3	$\{k_{CK}, \rho_X\}$	1	8.0
D1a $\alpha_{c,1p}$	$\{\varepsilon_{c0}, \gamma_0\}$	0	10.4	$\{k_{CK}, \rho_X\}$	0	8.4
D1a $\alpha_{c,2}$	$\{\varepsilon_{c0}, \gamma_0\}$	0	11.1	$\{k_c, \rho_X\}$	1	7.9
D1a $\alpha_{c,2p}$	$\{\varepsilon_{c0}\}$	NA	10.9	$\{k_c, \rho_X\}$	0	7.7
D1a $\alpha_{c,3}$	$\{\gamma_0, \zeta_2, \zeta_3, \gamma_2\}$	ND[a]	10.6	$\{\gamma_1, P_b\}$	ND[a]	10.6
D1a $\alpha_{c,3p}$	$\{\gamma_0, \zeta_3, P_a\}$	1	11.1	$\{\gamma_1, P_b, \zeta_2\}$	1	11.0
D1a $\alpha_{c,4p}$	$\{\gamma_0, \zeta_2, \zeta_1\}$	1	57.6	$\{\zeta_4\}$	NA	57.6
D1b $\alpha_{c,1}$	$\{\varepsilon_{c0}, \gamma_0\}$	0	10.1	$\{k_{CK}, \rho_X\}$	1	8.3
D1b $\alpha_{c,1p}$	$\{\varepsilon_{c0}, \gamma_0\}$	0	10.4	$\{k_{CK}, \rho_X\}$	0	8.4
D1b $\alpha_{c,2}$	$\{\varepsilon_{c0}, \gamma_0\}$	0	10.8	$\{k_c, \rho_X\}$	1	8.2
D1b $\alpha_{c,2p}$	$\{\varepsilon_{c0}\}$	NA	10.9	$\{k_c, \rho_X\}$	1	8.2
D1b $\alpha_{c,3}$	$\{\gamma_0, \zeta_3, P_b\}$	ND[a]	7.6	$\{\zeta_2\}$	NA	7.6
D1b $\alpha_{c,3p}$	$\{\gamma_0, \zeta_3, P_b, q_{m,MS}\}$	1	9.3	$\{P_a, \zeta_2\}$	ND[a]	9.3
D1b $\alpha_{c,4p}$	$\{\gamma_0, \zeta_1, \zeta_2, \zeta_3, \zeta_4\}$	6	157.3	NA	NA	NA
D1c $\alpha_{c,1}$	$\{\varepsilon_{c0}\}$	NA	10.9	$\{k_{CK}, \rho_X\}$	1	9.5
D1c $\alpha_{c,1p}$	$\{\varepsilon_{c0}\}$	NA	9.0	$\{k_{CK}, \rho_X\}$	1	7.7
D1c $\alpha_{c,2}$	$\{\varepsilon_{c0}\}$	NA	11.8	$\{k_c, \rho_X\}$	1	9.5
D1c $\alpha_{c,2p}$	$\{\varepsilon_{c0}\}$	NA	10.1	$\{k_c, \rho_X\}$	1	7.0
D1c $\alpha_{c,3}$	$\{\zeta_1, \zeta_2, P_b\}$	ND[a]	12.0	$\{\zeta_2\}$	NA	12.0
D1c $\alpha_{c,3p}$	$\{\zeta_2, P_b, P_a, q_{m,MS}\}$	3	10.5	$\{\zeta_2\}$	NA	10.5
D1c $\alpha_{c,4p}$	$\{\zeta_2, \zeta_3\}$	1	11.7	$\{q_{m,MS}, K_{S,c}\}$	0	11.7
D2 $\alpha_{c,1}$	$\{\varepsilon_{c0}, \beta_{ST}, C_d\}$	0	7.1	$\{k_{CK}, \rho_X\}$	0	7.1
D2 $\alpha_{c,1p}$	$\{\varepsilon_{c0}, \beta_{ST}, C_d\}$	1	8.1	$\{k_{CK}, \rho_X\}$	1	8.1
D2 $\alpha_{c,2}$	$\{\varepsilon_{c0}, \beta_{ST}, C_d\}$	0	7.0	$\{k_c, \rho_X\}$	1	7.0
D2 $\alpha_{c,2p}$	$\{\varepsilon_{c0}, \beta_{ST}, C_d\}$	0	7.8	$\{k_c, \rho_X\}$	1	7.8
D2 $\alpha_{c,3}$	$\{\beta_{ST}, C_d, P_b\}$	0	16.4	$\{\zeta_2\}$	NA	10.8
D2 $\alpha_{c,3p}$	$\{\beta_{ST}, C_d, P_b\}$	1	15.4	$\{\zeta_2\}$	NA	15.4
D2 $\alpha_{c,4p}$	$\{\beta_{ST}, C_d, \zeta_2\}$	1	15.6	NA	NA	NA
FR1	$\{\gamma_0, \gamma_1, \gamma_2, \gamma_3\}$	4	3.8	$\{K_F\}$	NA	3.8
FR2	$\{\gamma_0, \gamma_1, \gamma_3\}$	2	3.8	$\{K_F\}$	NA	3.8
FR3	$\{\gamma_0, \gamma_3\}$	1	3.5	$\{K_F\}$	NA	3.5
FR4	$\{\gamma_0, \gamma_1, \gamma_2, \gamma_3\}$	4	3.7	$\{K_F, \gamma_4\}$	1	3.7
FR5	$\{\gamma_0, \gamma_1, \gamma_3\}$	2	3.7	$\{K_F, \gamma_4\}$	1	3.7
FR6	$\{\gamma_0, \gamma_3\}$	1	3.4	$\{K_F, \gamma_4\}$	1	3.4

NA: not applicable

[a] Could not be determined due to numerical error: the Jacobian was zero for all values in at least one parameter resulting in division by zero when calculating the covariance matrix.

Table S16. Optimised parameter values ($\hat{\theta}$) for the alternate AnMBR empirical fouling rate (FR) models. Relative error $\sigma_\theta/\hat{\theta}$ shown between brackets. θ^o is the initial guess (nominal values).

↓Model	K_F	γ_0	γ_1	γ_2	γ_3	γ_4
Units→	×10^{-4} Pa s^{-1}	×10^{6} s m^{-1}	×10^{8} s^{2} m^{-1}	×10^{4} s m^{-2} kg^{-1}	×10^{6} s m^{-2} kg^{-1}	×10^{10} s m^{-2}
θ^o→	5.6	2.81	2.48	5.1	1.28	1.75
FR1	5.60 [0.01]	4.19 [0.004]	5.44 [0.03]	3.92 [5.93]	1.10 [0.03]	
FR2	5.60 [0.01]	4.19 [0.003]	5.42 [0.02]		1.09 [0.03]	
FR3	5.60 [0.01]	3.08 [0.003]			1.12 [0.02]	
FR4	6.39 [0.11]	4.65 [0.004]	5.19 [0.03]	38.11 [0.60]	0.83 [0.04]	1.94 [0.08]
FR5	6.21 [0.11]	4.67 [0.003]	5.03 [0.02]		0.80 [0.04]	1.90 [0.08]
FR6	5.75 [0.10]	3.60 [0.002]			0.88 [0.02]	1.79 [0.08]

Table S17. Optimised parameter values ($\hat{\theta}$) for the alternate AnMBR filtration models. Relative error $\sigma_\theta/\hat{\theta}$ shown between brackets. θ^o is the initial guess (nominal values).

↓Model	C_d	$K_{S,\mathrm{c}}$	$q_{m,\mathrm{MS}}$	β_{ST}
Units→	-	kg	-	-
θ^o→	0.40	0.2	4.71	1.75×10^{4}
D1b $\alpha_{\mathrm{c},3p}$			1.4×10^{4} [3×10^{-5}]	
D1c $\alpha_{\mathrm{c},3p}$			3.04 [1×10^{-5}]	
D1c $\alpha_{\mathrm{c},4p}$		0.32 [3×10^{-5}]	6.93 [2×10^{-5}]	
D2 $\alpha_{\mathrm{c},1}$	0.31 [7×10^{-7}]			2.28×10^{-4} [6×10^{-4}]
D2 $\alpha_{\mathrm{c},1p}$	0.40 [6×10^{-7}]			1.78×10^{-4} [0.001]
D2 $\alpha_{\mathrm{c},2}$	0.39 [4×10^{-7}]			2.37×10^{-4} [6×10^{-4}]
D2 $\alpha_{\mathrm{c},2p}$	0.47 [4×10^{-7}]			1.96×10^{-4} [9×10^{-4}]
D2 $\alpha_{\mathrm{c},3}$	0.33 [0.01]			0.505 [0.01]
D2 $\alpha_{\mathrm{c},3p}$	0.30 [1×10^{-4}]			4.5×10^{-7} [1.40]
D2 $\alpha_{\mathrm{c},4p}$	0.34 [1×10^{-5}]			2.3×10^{-6} [0.57]

(continued)

Table S17 continued. Optimised parameter values ($\hat{\theta}$) for the alternate AnMBR filtration models. Relative error $\sigma_\theta/\hat{\theta}$ shown between brackets. θ^o is the initial guess (nominal values).

↓Model	γ_0	γ_1	γ_2
Units→	$\times10^6$ s m^{-1}	$\times10^8$ s^2 m^{-1}	s m^{-2} kg^{-1}
θ^o→	2.81	2.48	5.1×10^4
D1a $\alpha_{c,1}$	2.52 [2×10^{-5}]		
D1a $\alpha_{c,1p}$	5.59 [1×10^{-8}]		
D1a $\alpha_{c,2}$	2.47 [2×10^{-5}]		
D1a $\alpha_{c,3}$	33.0 [∞^a]	2.47 [∞^a]	0.5 [∞^a]
D1a $\alpha_{c,3p}$	3.03 [4×10^{-5}]	3.59 [3×10^{-5}]	
D1a $\alpha_{c,4p}$	2.72 [8×10^{-8}]		
D1b $\alpha_{c,1}$	2.46 [3×10^{-5}]		
D1b $\alpha_{c,1p}$	5.27 [1×10^{-9}]		
D1b $\alpha_{c,2}$	2.26 [3×10^{-5}]		
D1b $\alpha_{c,3}$	33.8 [∞^a]		
D1b $\alpha_{c,3p}$	3.69 [3×10^{-5}]		
D1b $\alpha_{c,4p}$	2.85 [7×10^{-8}]		

[a] The Jacobian was zero for all values in at least one parameter resulting in division by zero when calculating the covariance matrix.

(continued)

Table S17 continued. Optimised parameter values ($\hat{\theta}$) for the alternate AnMBR filtration models. Relative error $\sigma_\theta/\hat{\theta}$ shown between brackets. θ^o is the initial guess (nominal values).

↓Model	k_c	k_{CK}	$\varepsilon_{\mathrm{c0}}$	ρ_{X}	P_{a}
Units→	$\times 10^{17}$ m^{-2}	$\times 10^{7}$	-	$\times 10^{3}$ kg m^{-3}	$\times 10^{4}$ Pa
θ^o→	1.0	4.0	0.66	1.24	2.01
D1a $\alpha_{\mathrm{c},1}$		21 [0.04]	0.38 [0.01]	18.4 [0.03]	
D1a $\alpha_{\mathrm{c},1p}$		35 [0.01]	0.57 [0.003]	20.6 [0.04]	
D1a $\alpha_{\mathrm{c},2}$	6.12 [0.04]		0.44 [0.01]	23.9 [0.03]	
D1a $\alpha_{\mathrm{c},2p}$	9.53 [0.01]		0.61 [0.003]	22.8 [0.02]	
D1a $\alpha_{\mathrm{c},3p}$				20.5 [0.04]	1.07 [0.03]
D1b $\alpha_{\mathrm{c},1}$		21 [0.04]	0.41 [0.01]	20.6 [0.04]	
D1b $\alpha_{\mathrm{c},1p}$		35 [0.01]	0.57 [0.003]	25.0 [0.03]	
D1b $\alpha_{\mathrm{c},2}$	5.60 [0.04]		0.46 [0.01]	25.0 [0.03]	
D1b $\alpha_{\mathrm{c},2p}$	9.33 [0.01]		0.63 [0.003]		
D1b $\alpha_{\mathrm{c},3p}$					7.8×10^{12}[0.06]
D1c $\alpha_{\mathrm{c},1}$		15.71 [0.04]	0.31 [0.01]	12.1 [0.05]	
D1c $\alpha_{\mathrm{c},1p}$		17.11 [0.02]	0.43 [0.002]	7.29 [0.04]	
D1c $\alpha_{\mathrm{c},2}$	4.73 [0.05]		0.37 [0.01]	16.8 [0.04]	
D1c $\alpha_{\mathrm{c},2p}$	5.94 [0.01]		0.49 [0.002]	10.6 [0.02]	
D1c $\alpha_{\mathrm{c},3p}$					0.69 [0.20]
D2 $\alpha_{\mathrm{c},1}$		4 [0.02]	0.12 [0.004]	1.24 [0.03]	
D2 $\alpha_{\mathrm{c},1p}$		4 [0.01]	0.16 [0.002]	1.23 [0.02]	
D2 $\alpha_{\mathrm{c},2}$	0.99 [0.02]		0.13 [0.004]	1.25 [0.02]	
D2 $\alpha_{\mathrm{c},2p}$	1.00 [0.01]		0.17 [0.002]	1.23 [0.02]	

(continued)

Table S17 continued. Optimised parameter values ($\hat{\theta}$) for the alternate AnMBR filtration models. Relative error $\sigma_\theta/\hat{\theta}$ shown between brackets. θ^o is the initial guess (nominal values).

↓Model	P_b	ζ_1	ζ_2	ζ_3	ζ_4
Units→	×10³ Pa	×10³	×10⁴	-	-
θ^o→	4.1799	1.16	1.36	172.4	150.9
D1a $\alpha_{c,3}$	4.22 [∞[a]]		14.9 [∞[a]]	111 [∞[a]]	
D1a $\alpha_{c,3p}$	4.26 [1×10^5]		1.4 [1×10^5]	1037 [705]	
D1a $\alpha_{c,4p}$		1.17 [0.001]		177 [2×10^{-4}]	150.9 [5×10^{-4}]
D1b $\alpha_{c,3}$	137.6 [∞[a]]		1.36 [0.03]	66.7 [∞[a]]	
D1b $\alpha_{c,3p}$	58.1 [0.02]		2.34 [∞[a]]	106.2 [0.02]	
D1b $\alpha_{c,4p}$		1.13 [2.01]	1.38 [0.17]	173.5 [0.01]	156.9 [0.25]
D1c $\alpha_{c,3}$	58.7 [∞[a]]		1.36 [0.04]	1752 [∞[a]]	
D1c $\alpha_{c,3p}$	9.46 [0.18]		1.36 [0.005]	127.9 [0.01]	
D1c $\alpha_{c,4p}$			1.98 [0.01]	381.1 [0.01]	
D2 $\alpha_{c,3}$	7.23 [8×10^4]		2.04×10^6 [0.04]		
D2 $\alpha_{c,3p}$	8.1 [0.61]		1.36 [0.02]		
D2 $\alpha_{c,4p}$			2.52 [0.47]		

[a] The Jacobian was zero for all values in at least one parameter resulting in division by zero when calculating the covariance matrix.

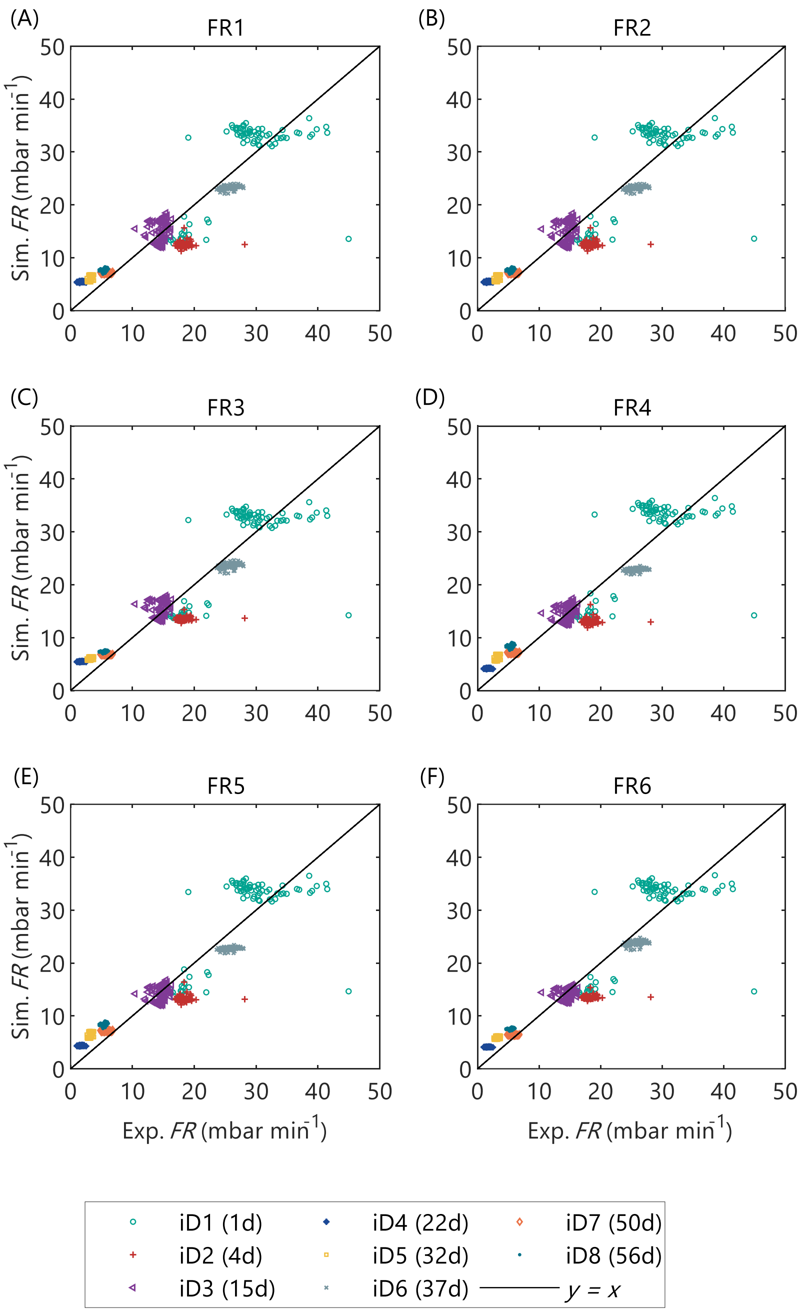

Figure S10. Calibration of the alternate AnMBR empirical fouling rate models, FR1 to FR6, using the representative dataset from the pilot AnMBR, iD1 to iD8.

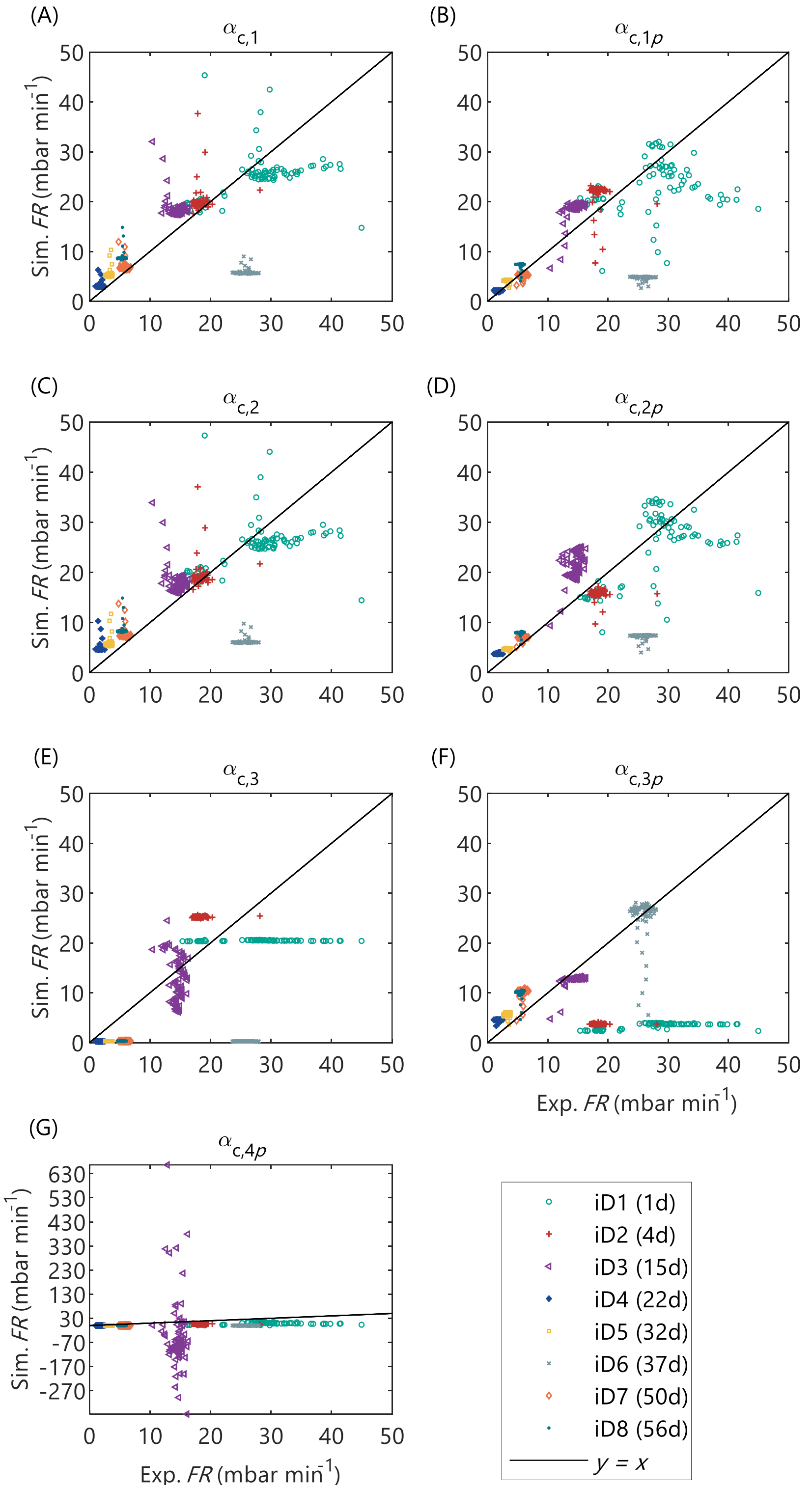

Figure S11. Calibration of the alternate AnMBR filtration models with deposition submodel D1a and different specific cake resistance submodels, $\alpha_{c,1}$ to $\alpha_{c,4p}$; using the representative dataset from the pilot AnMBR, iD1 to iD8.

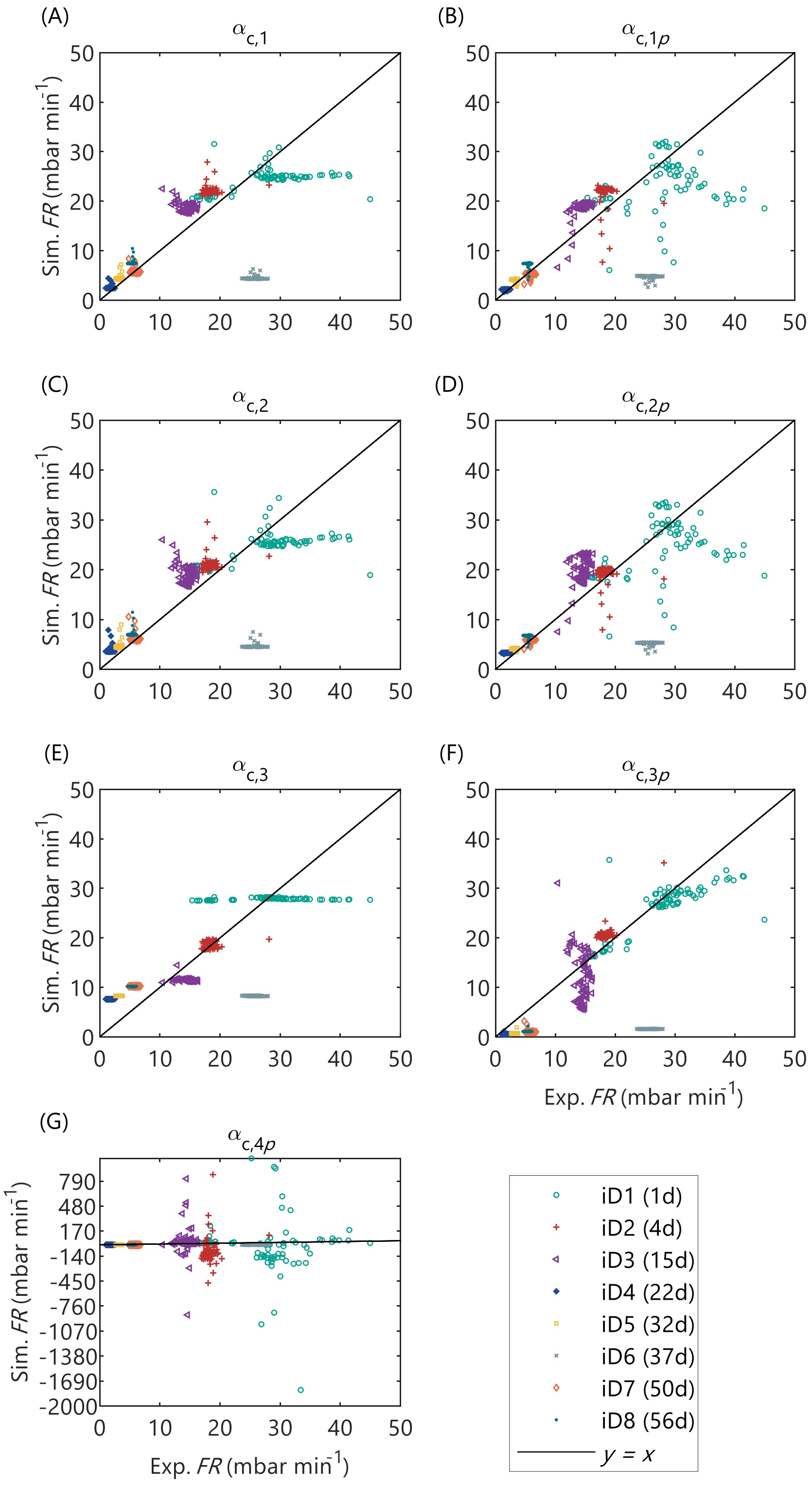

Figure S12. Calibration of the alternate AnMBR filtration models with deposition submodel D1b and different specific cake resistance submodels, $\alpha_{c,1}$ to $\alpha_{c,4p}$; using the representative dataset from the pilot AnMBR, iD1 to iD8..

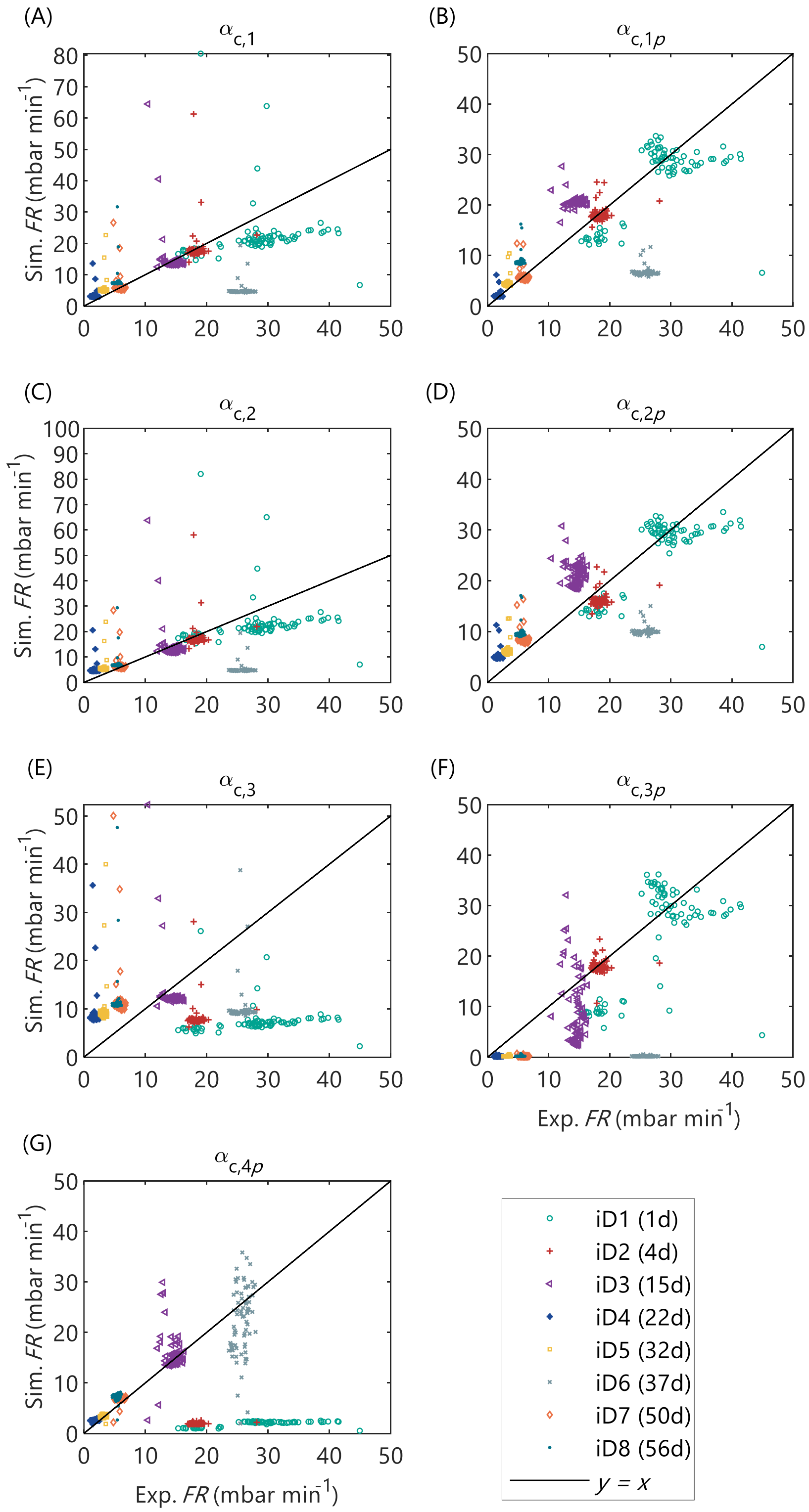

Figure S13. Calibration of the alternate AnMBR filtration models with deposition submodel D1c and different specific cake resistance submodels, $\alpha_{c,1}$ to $\alpha_{c,4p}$; using the representative dataset from the pilot AnMBR, iD1 to iD8..

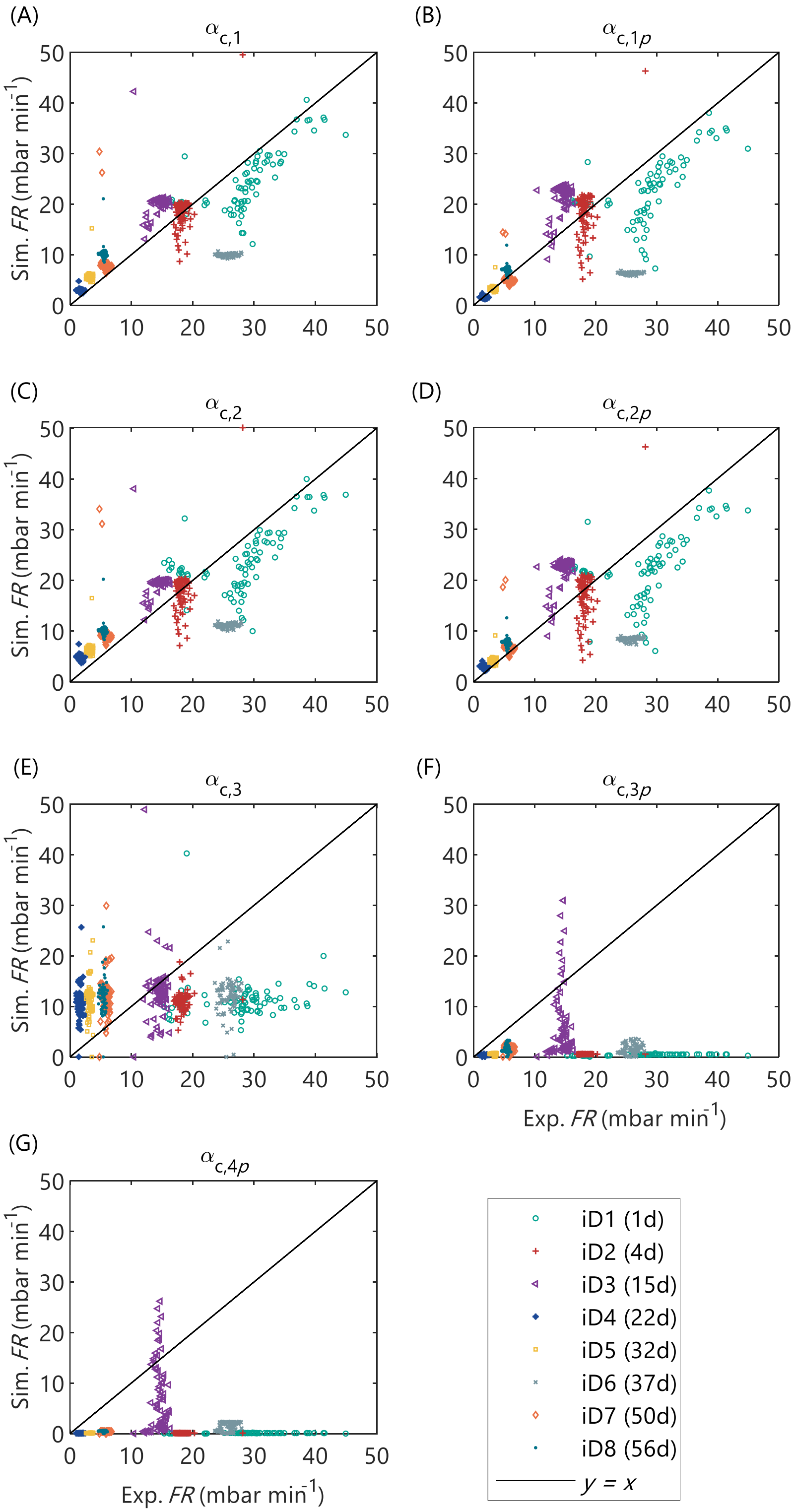

Figure S14. Calibration of the alternate AnMBR filtration models with deposition submodel D2 and different specific cake resistance submodels, $\alpha_{c,1}$ to $\alpha_{c,4p}$; using the representative dataset from the pilot AnMBR, iD1 to iD8.

Long-term prediction with calibrated models

Table S18. Statistical indicators representing model accuracy of the alternate AnMBR filtration models for long-term fouling rate prediction in the pilot AnMBR. Normalised root mean square error (nRMSE), Pearson correlation coefficient (Corr), and mean absolute percentage error (MAPE).

Model	nRMSE	Corr	MAPE
D1a $\alpha_{c,1}$	0.71	0.68	58
D1a $\alpha_{c,1p}$	0.80	0.66	37
D1a $\alpha_{c,2}$	0.74	0.67	90
D1a $\alpha_{c,2p}$	0.70	0.69	72
D1a $\alpha_{c,3}$	0.86	0.73	79
D1a $\alpha_{c,3p}$	20.48	0.03	145
D1a $\alpha_{c,4p}$	15.52	0.00	122
D1b $\alpha_{c,1}$	0.74	0.69	43
D1b $\alpha_{c,1p}$	0.80	0.66	37
D1b $\alpha_{c,2}$	0.77	0.68	64
D1b $\alpha_{c,2p}$	0.87	0.65	57
D1b $\alpha_{c,3}$	0.97	0.53	161
D1b $\alpha_{c,3p}$	0.76	0.75	64
D1b $\alpha_{c,4p}$	36,204	0.01	15,396
D1c $\alpha_{c,1}$	0.76	0.62	54
D1c $\alpha_{c,1p}$	0.70	0.69	38
D1c $\alpha_{c,2}$	0.78	0.60	80
D1c $\alpha_{c,2p}$	0.70	0.70	103
D1c $\alpha_{c,3}$	1.05	-0.28	181
D1c $\alpha_{c,3p}$	0.95	0.67	80
D1c $\alpha_{c,4p}$	81.7	-0.01	142
D2 $\alpha_{c,1}$	0.73	0.73	74
D2 $\alpha_{c,1p}$	2.34	0.38	52
D2 $\alpha_{c,2}$	14.99	0.06	713
D2 $\alpha_{c,2p}$	20.33	0.06	110
D2 $\alpha_{c,3}$	1.05	-0.05	219
D2 $\alpha_{c,3p}$	774	0.02	1,014
D2 $\alpha_{c,4p}$	2,369	0.00	2,443
FR1	0.55	0.82	93
FR2	0.55	0.82	93
FR3	0.69	0.73	88
FR4	0.54	0.83	74
FR5	0.54	0.83	78
FR6	0.70	0.72	66

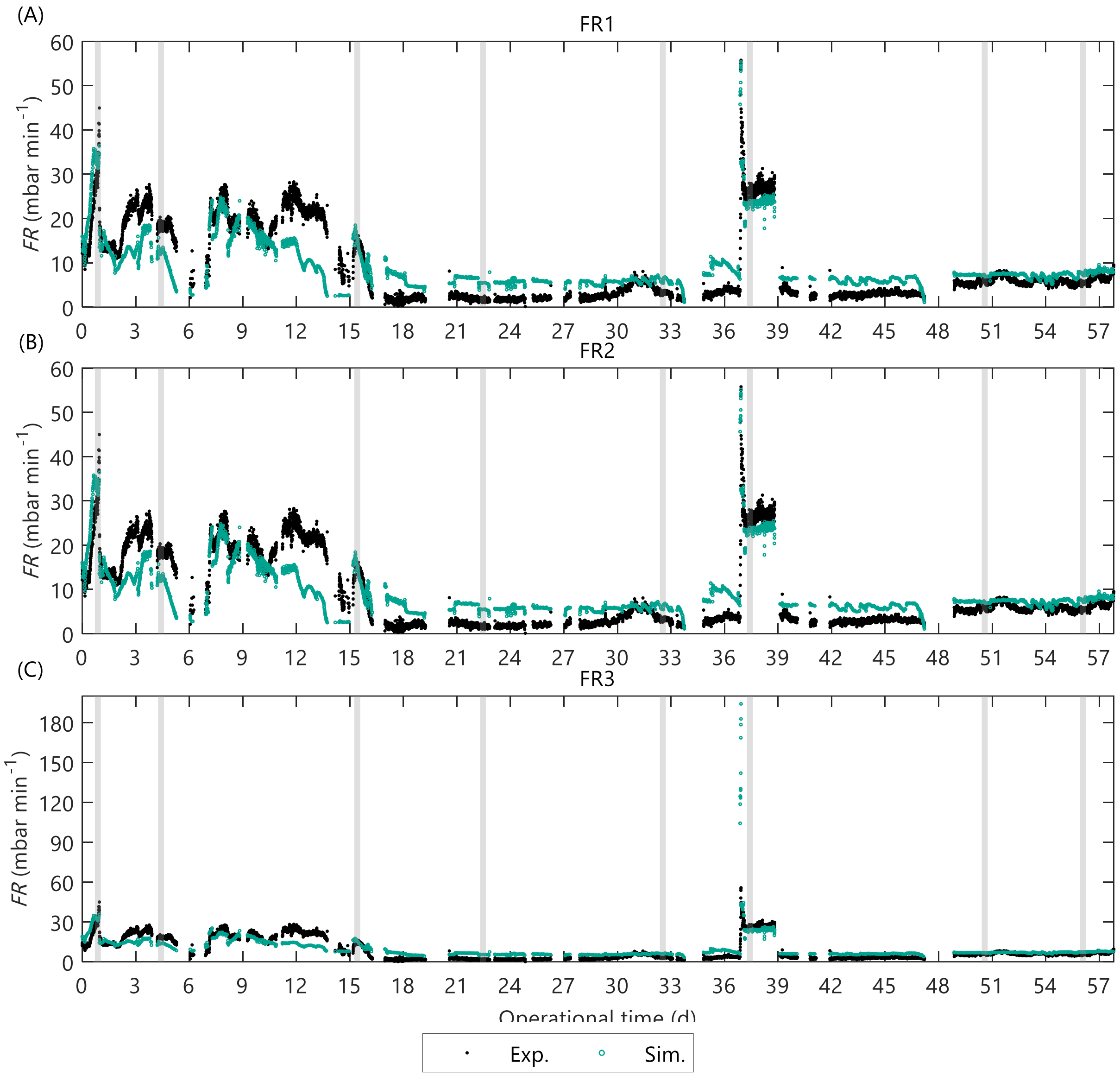

Figure S15. Validation of the alternate AnMBR empirical fouling rate (FR) models that excluded floc size as input variable: (A) FR1, (B) FR2, and (C) FR3. The grey vertical areas represent the representative dataset (iD1 to iD8 from left to right) used for model calibration.

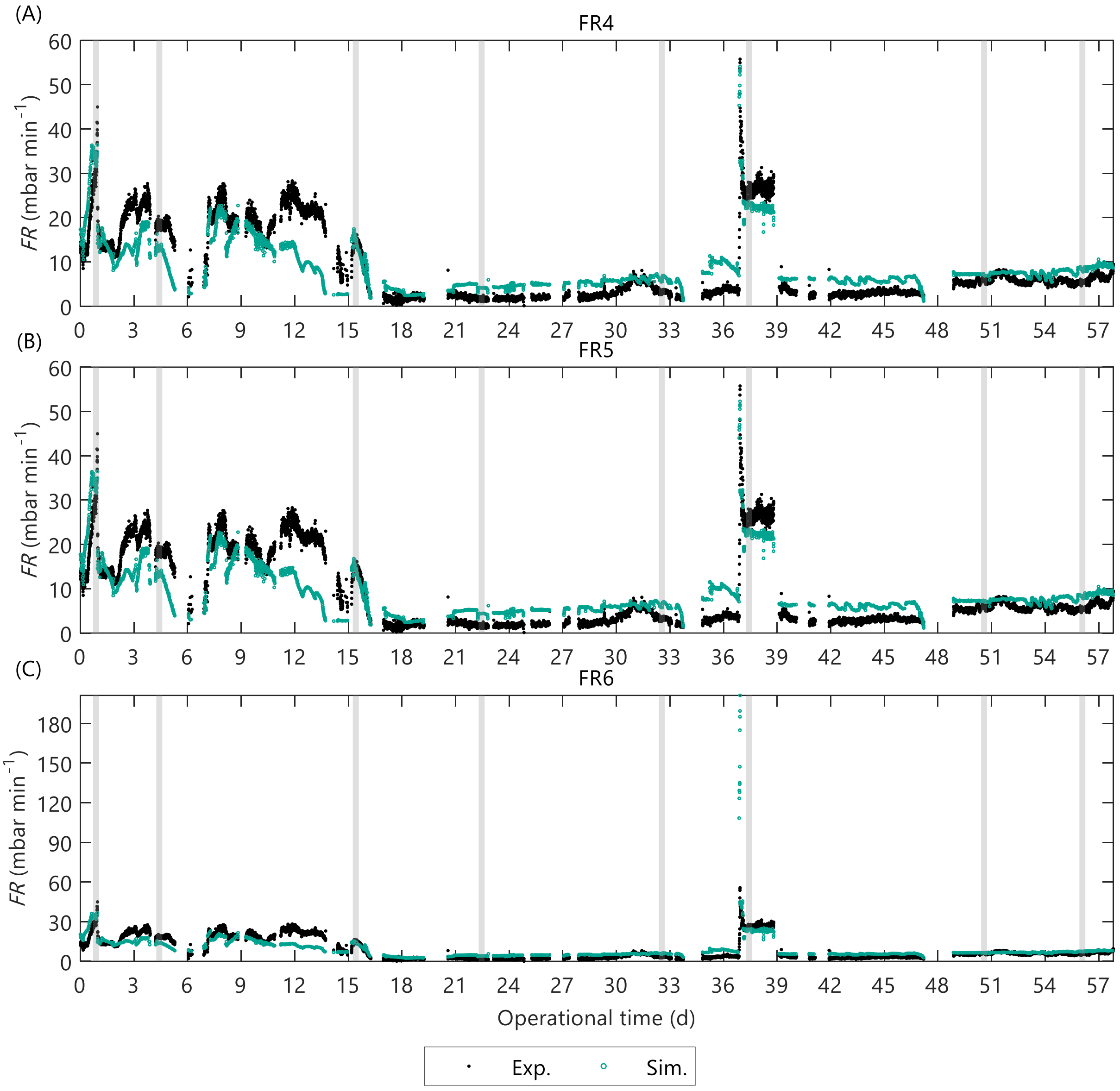

Figure S16. Validation of the alternate AnMBR empirical fouling rate (FR) models that included floc size as input variable: (A) FR4, (B) FR5, and (C) FR6.

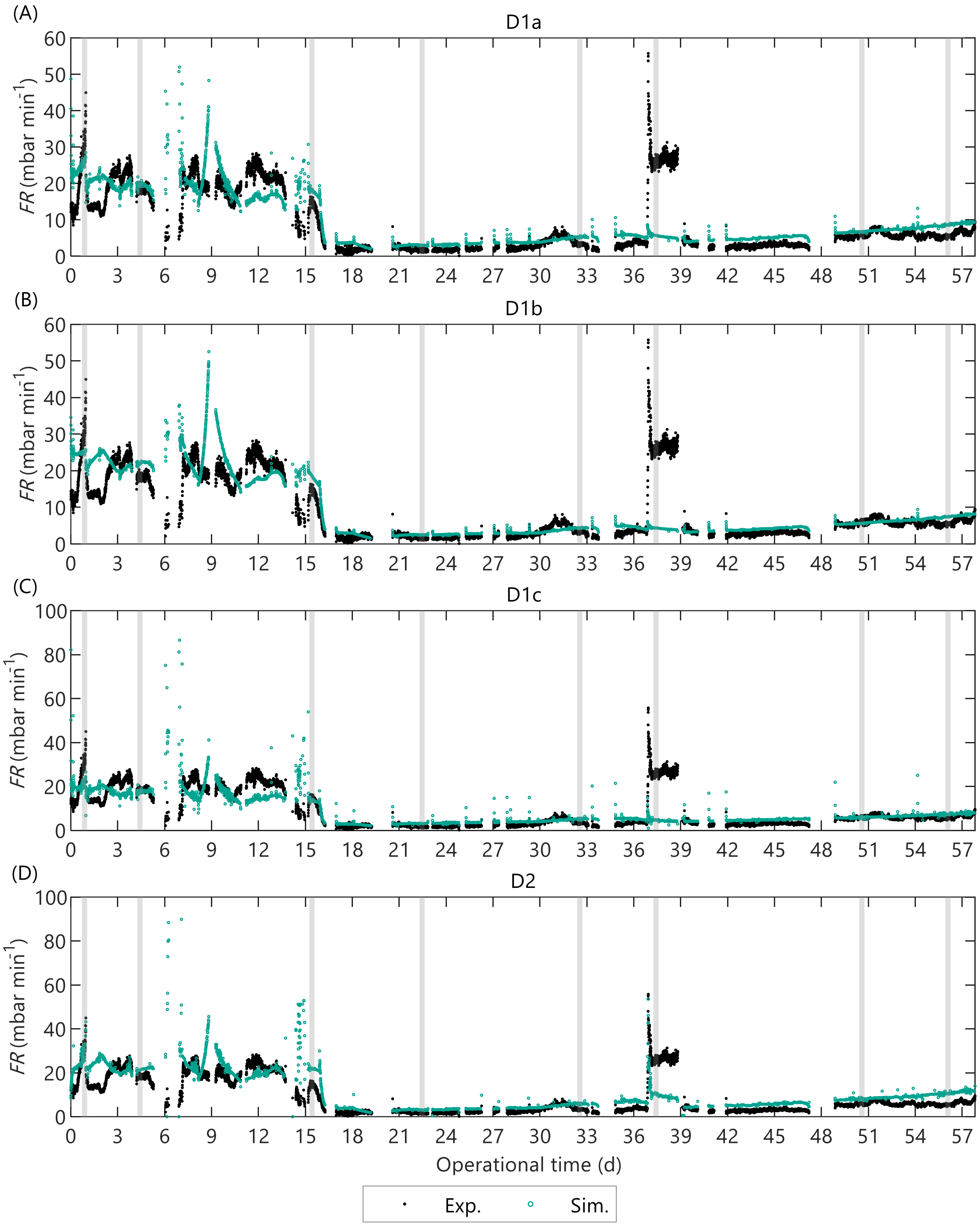

Figure S17. Validation of the alternate AnMBR filtration models that combine the specific cake resistance model $\alpha_{c,1}$ with the different deposition submodels: (A) D1a, (B) D1b, (C) D1c and (D) D2.

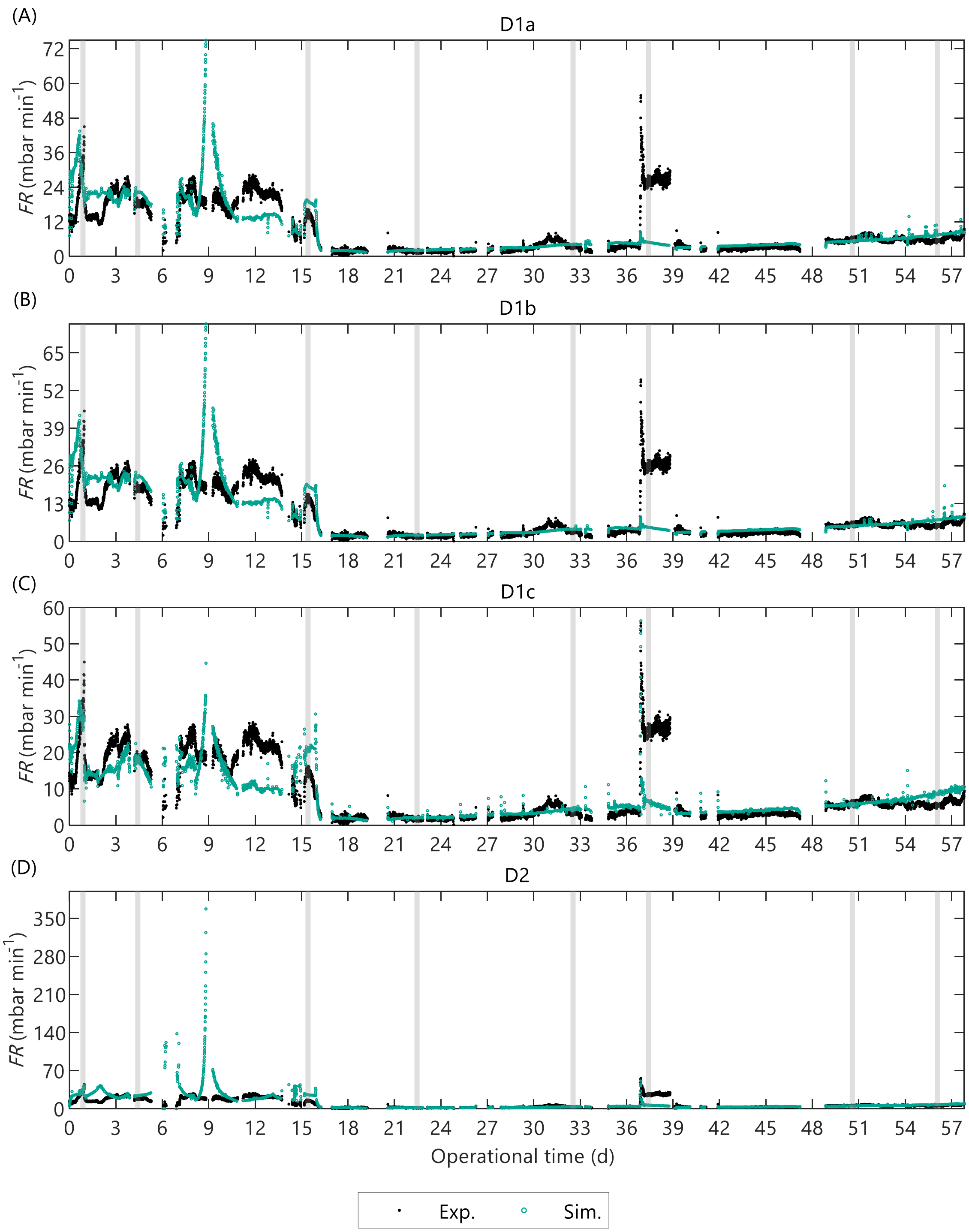

Figure S18. Validation of the alternate AnMBR filtration models that combine the specific cake resistance submodel $\alpha_{c,1p}$ with the different deposition submodels: (A) D1a, (B) D1b, (C) D1c and (D) D2.

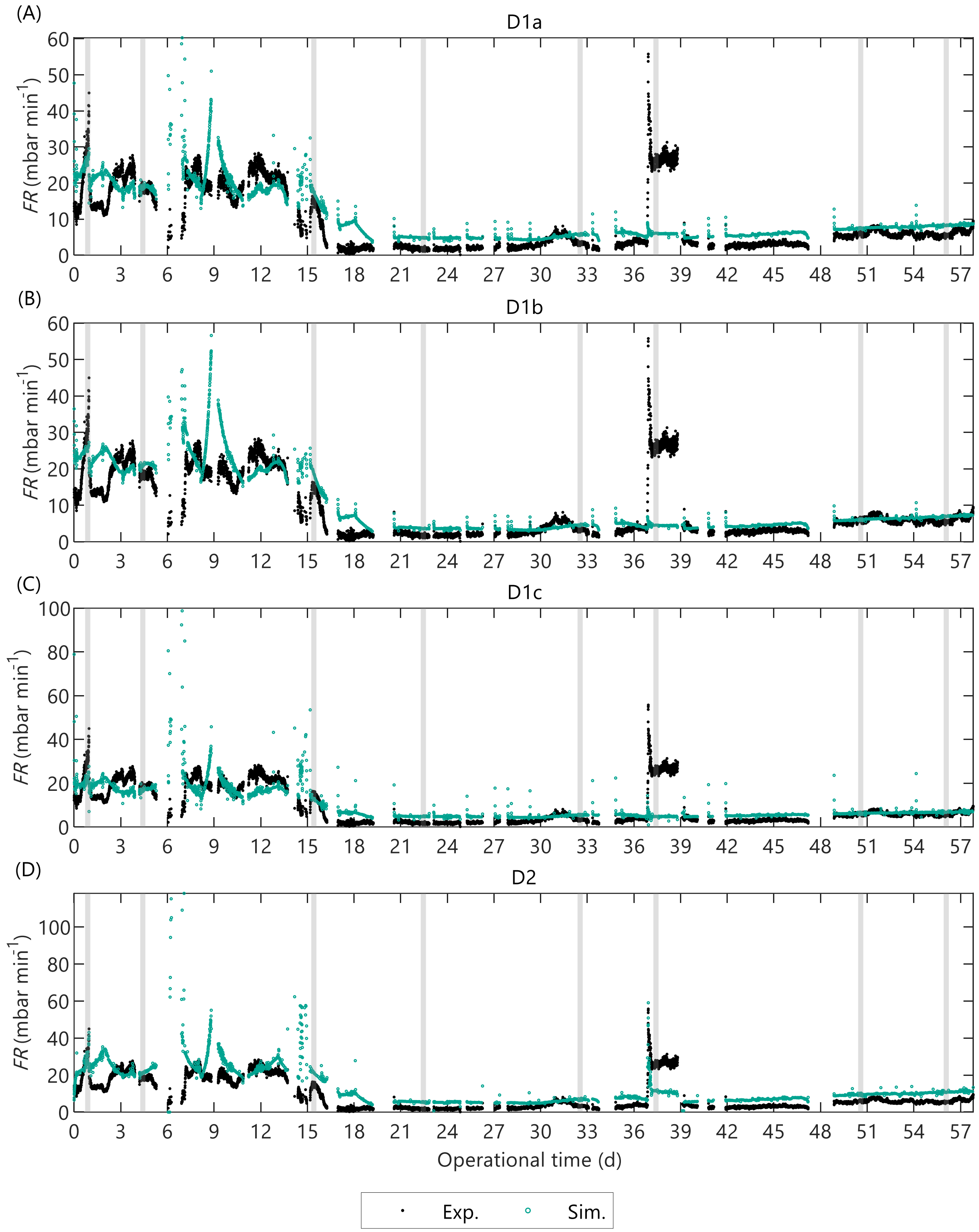

Figure S19. Validation of the alternate AnMBR filtration models that combine the specific cake resistance submodel $\alpha_{c,2}$ with the different deposition submodels: (A) D1a, (B) D1b, (C) D1c and (D) D2.

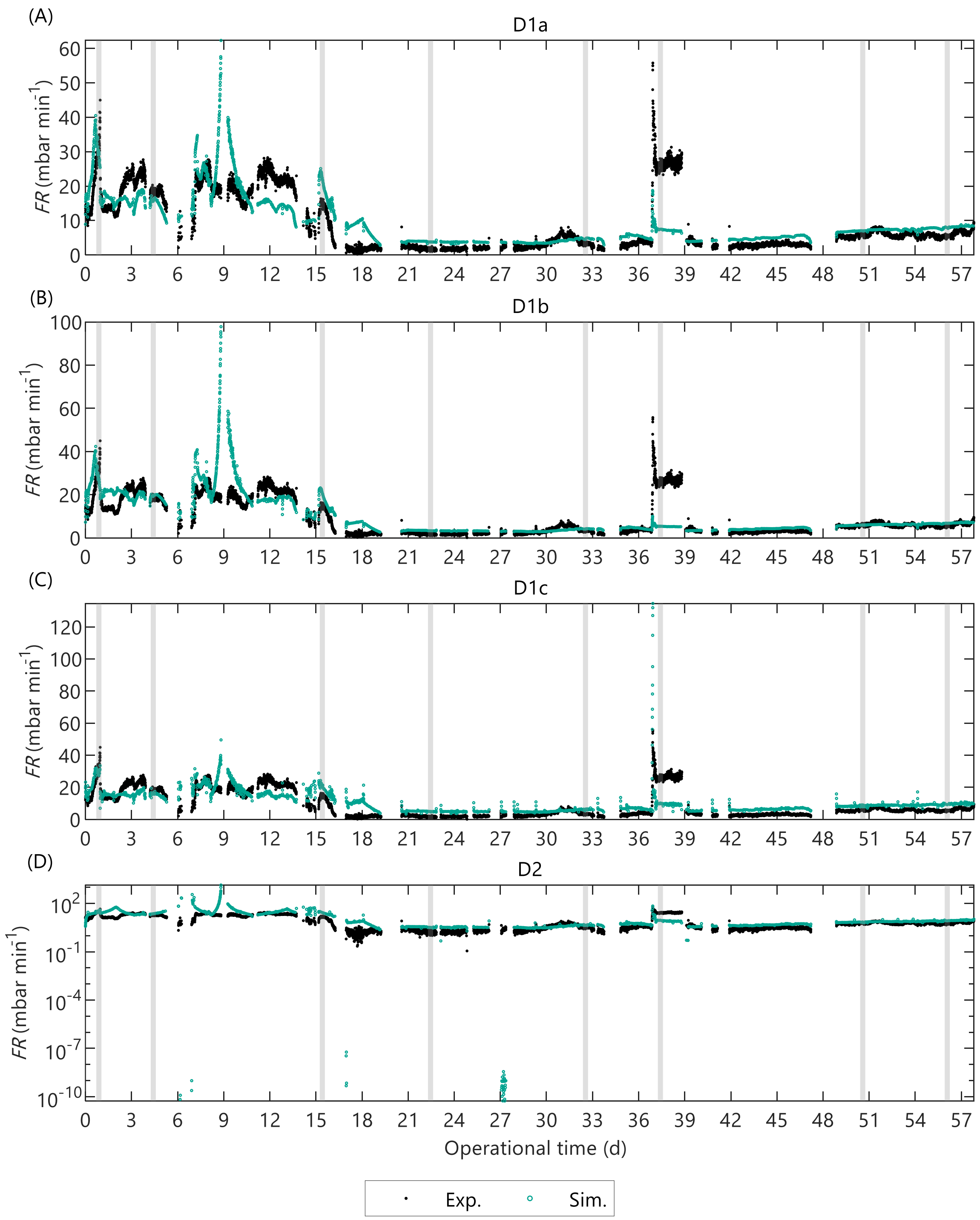

Figure S20. Validation of the alternate AnMBR filtration models that combine the specific cake resistance submodel $\alpha_{c,2p}$ with the different deposition submodels: (A) D1a, (B) D1b, (C) D1c and (D) D2.

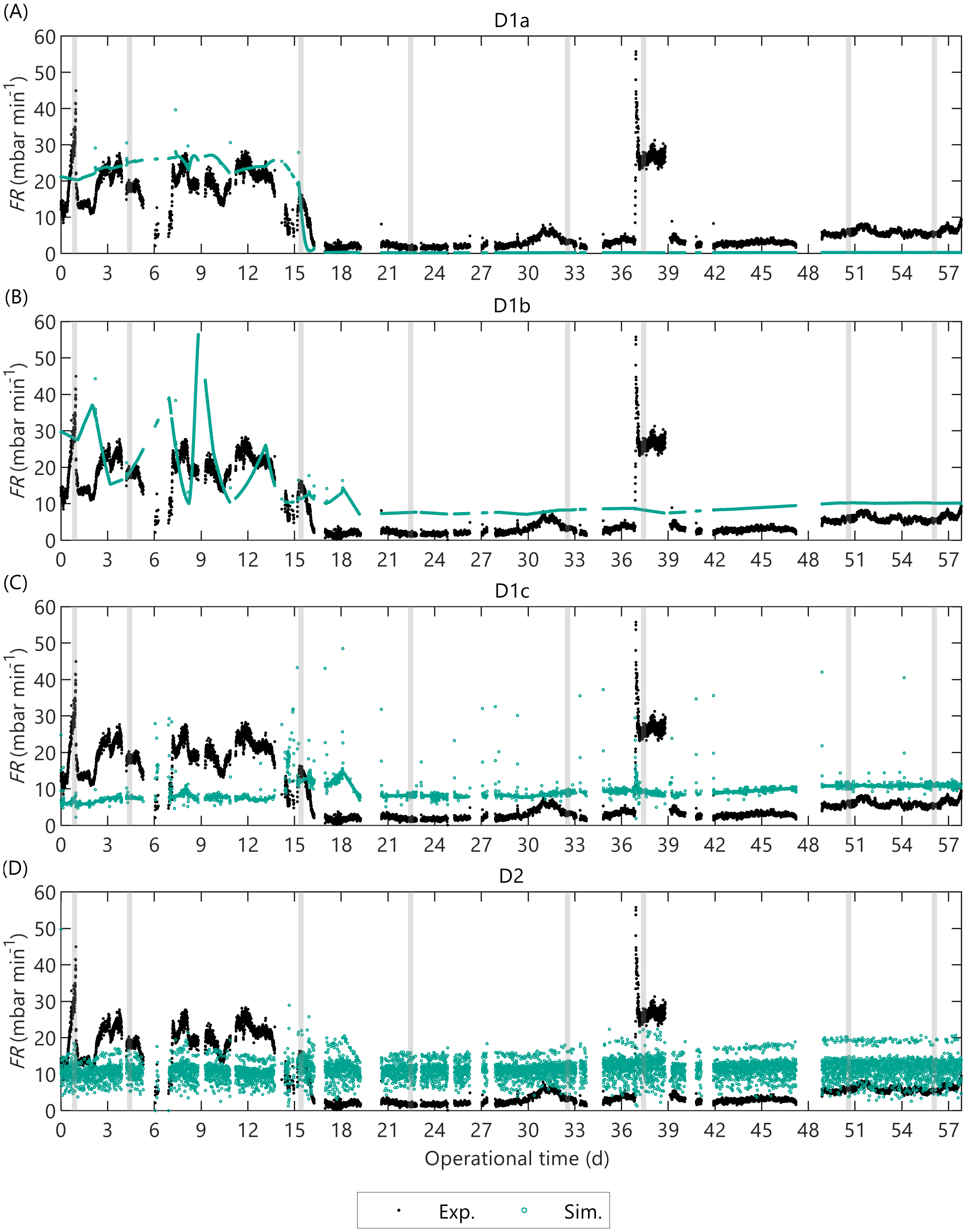

Figure S21. Validation of the alternate AnMBR filtration models that combine the specific cake resistance submodel $\alpha_{c,3}$ with the different deposition submodels: (A) D1a, (B) D1b, (C) D1c and (D) D2.

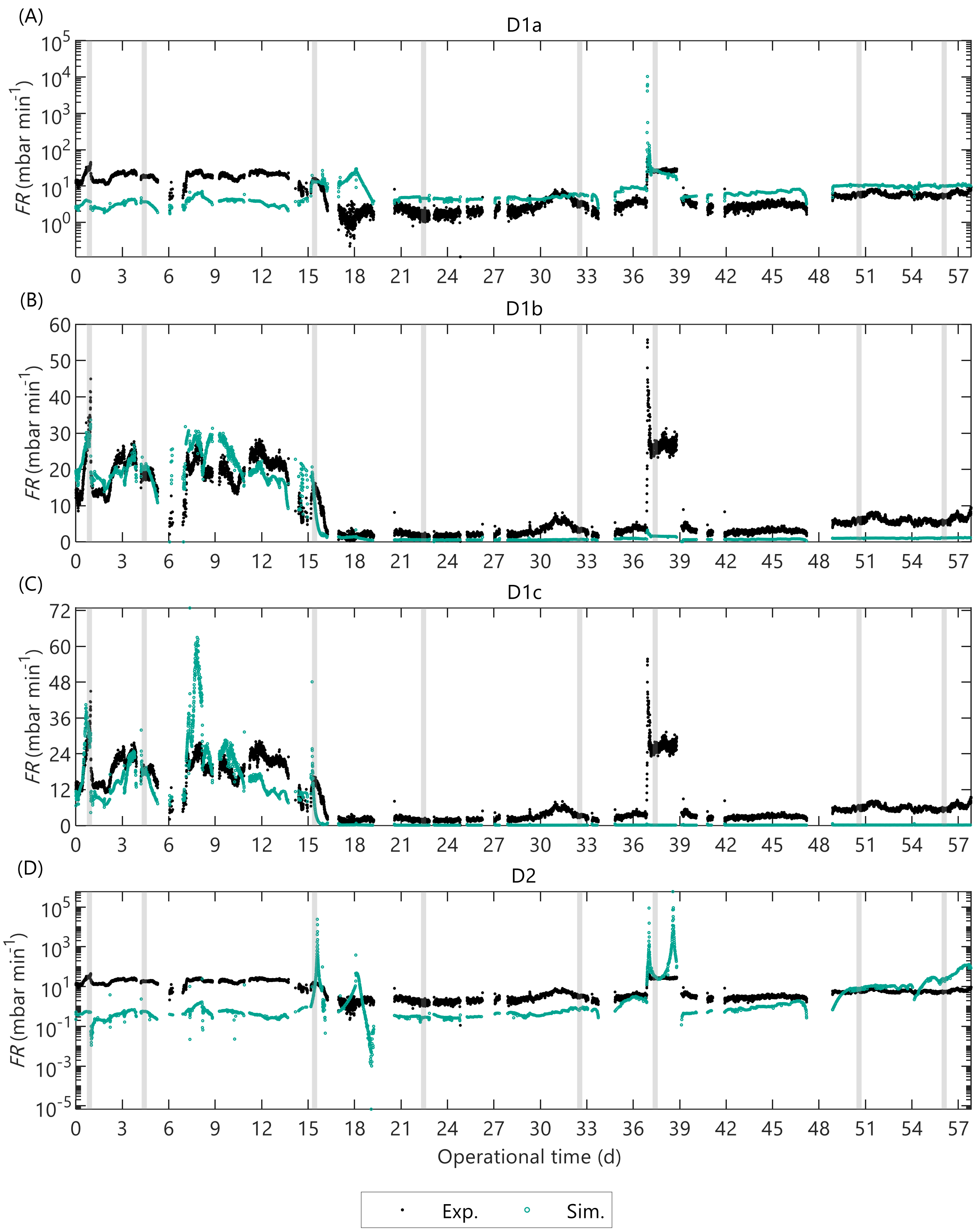

Figure S22. Validation of the alternate AnMBR filtration models that combine the specific cake resistance submodel $\alpha_{c,3p}$ with the different deposition submodels: (A) D1a, (B) D1b, (C) D1c and (D) D2.

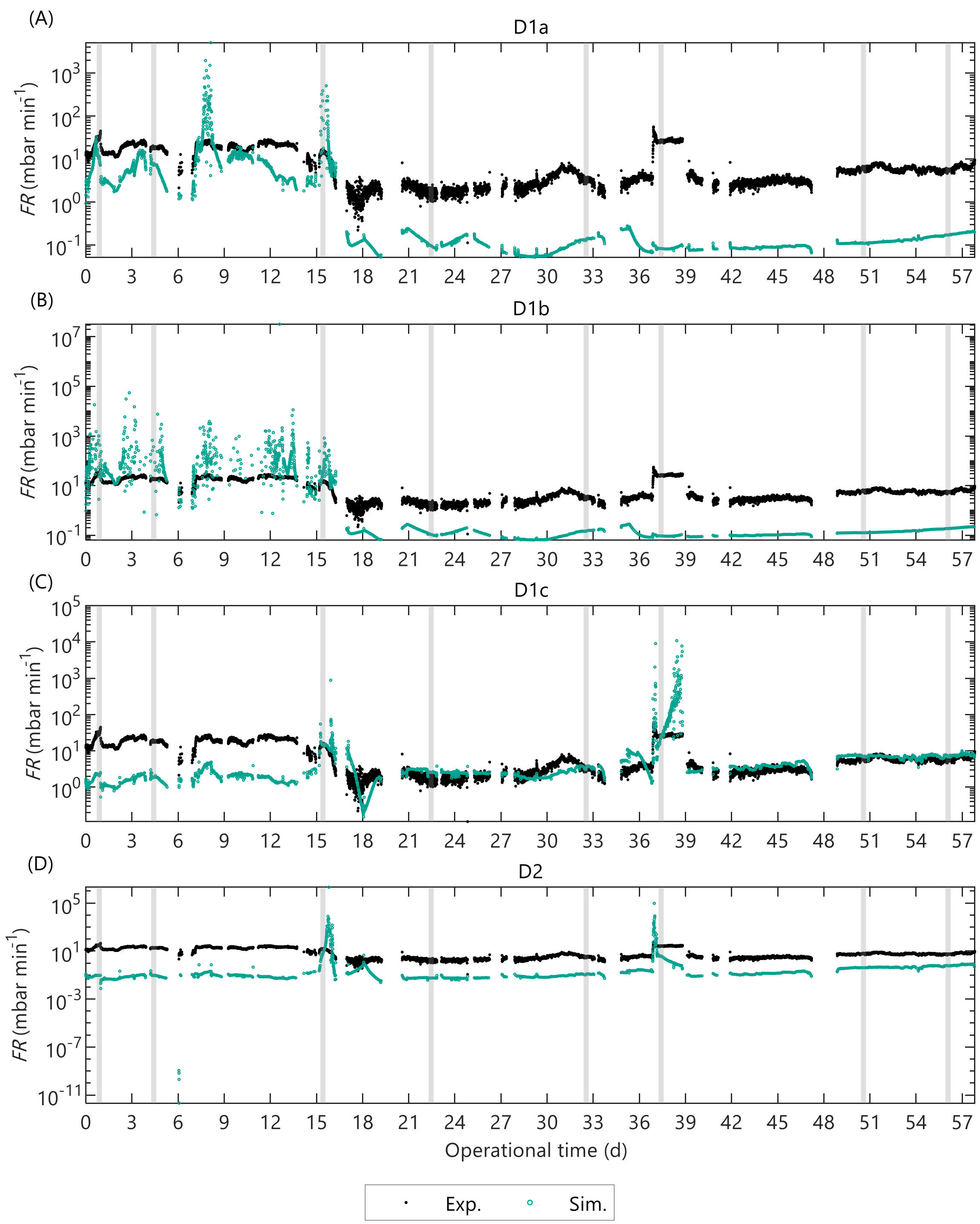

Figure S23. Validation of the alternate AnMBR filtration models that combine the specific cake resistance submodel $\alpha_{c,4p}$ with the different deposition submodels: (A) D1a, (B) D1b, (C) D1c and (D) D2.

S10. Effect of sludge characteristics on fouling rate predictions

Figure S24 show the effect of the sludge characteristics on the predicted fouling rate with three calibrated alternate AnMBR filtration models, namely FR6, D1c α_1, and D1c $\alpha_{1,p}$. The sludge characteristics were varied inside the simulation ranges in Figure S24, which were based on the observed ranges in the pilot AnMBR. The remaining variables were set to the nominal values presented in Table S1 and Table S19.

Each sludge characteristics was divided in 50 values inside its range, creating a 50x50 mesh for each plot in Figure S24, the value for each datapoint in the mesh was the predicted FR. For the empirical model, the corresponding equation to calculate FR was directly applied for each value in the mesh. For the FR_RIS models, the FR was obtained by solving the model at fixed sludge characteristics until steady state was reached, and the last FR value was used. Two hours was sufficient to reach steady state as verified by analysing the evolution of the model (results not shown).

Table S19. Nominal values and simulation range of operational conditions and sludge characteristics.

	Variable	Units	Nominal value [a]	Range in pilot AnMBR	Simulation range
c_{C}	Concentration of colloidal material	Kg m^{-3}	0.35	[0.14, 0.62]	[0.10, 0.65]
c_{X}	Concentration of particulate material	Kg m^{-3}	9.25	[4.9, 15.9]	[4.0, 16.0]
d_p	Mean particle diameter	×10^{-5} m	2.7	[2.1, 4.5]	[2.0, 5.0]
u_{G}	Superficial gas velocity	×10^{-3} m s^{-1}	3.0	[0.5, 5.7]	[0.5 6.0]

[a] Mean value during pilot AnMBR operation.

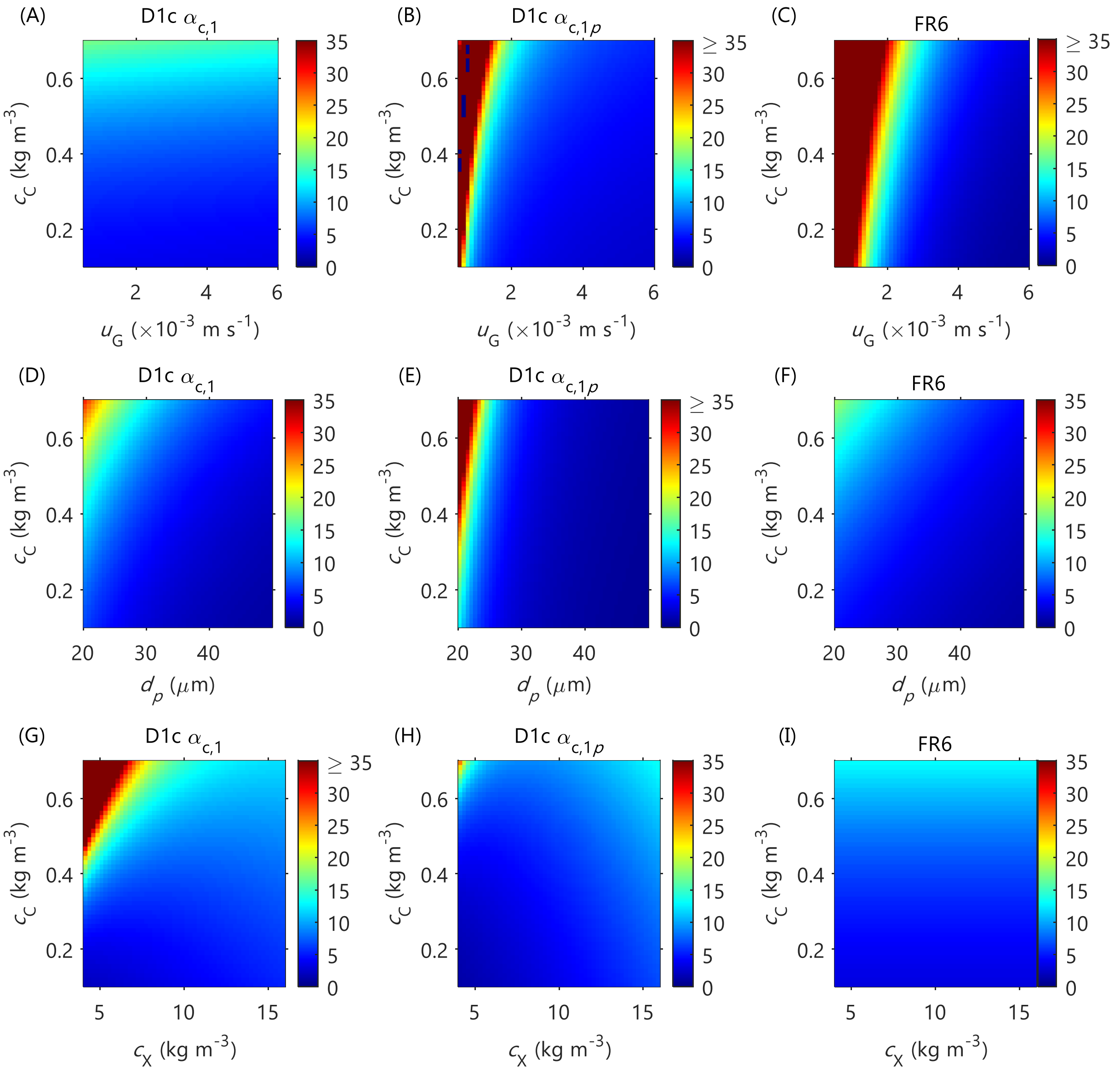

Figure S24. Effect of colloidal material concentration (c_C), particulate material concentration (c_X), mean particle diameter (d_p), and superficial gas velocity in the membrane tank (u_G) on the predicted fouling rate (FR, mbar min^{-1}) with different calibrated AnMBR filtration models: (left) D1c α_1, (middle) D1c $\alpha_{1,p}$, and (right) FR6.

S11. Calibration and validation of alternate AnDFCm filtration models

Model calibration discussion

Table S20 summarises the GSA results for all alternate AnDFCm filtration models. Analogous to the GSA results for the alternate AnMBR filtration models, the parameters C_d, β_{ST}, k_c, k_{CK}, $\varepsilon_{\mathrm{c0}}$, ρ_{X}, P_{b}, ζ_2 and ζ_3 were influential in all or most models; h_t, γ, ρ_{C}, ζ_1, and ζ_4 were none-influential in all or most models; and $\varepsilon_{\mathrm{c0}}$ was the most influential parameter in all the models. Opposite to the GSA results for the alternate AnMBR filtration models, $K_{S,\mathrm{c}}$ and $q_{m,\mathrm{MS}}$ (which are associated with the detachment processes) were only influential in some AnMBR models, whereas they were influential in most AnDFCm models. This might be caused by the higher superficial velocity in the AnDFCm installation ($u_{\mathrm{L,AnDFCm}}$=1.5 m s^{-1}) compared to the AnMBR ($0.5\times10^{-3} < u_{\mathrm{G}} < 5.7\times10^{-3}$ m s^{-1}) which increases the detachment.

The influential parameters were estimated using in-situ ΔR_{20} measurement in the pilot AnMBR immediately after FE dosing and ex-situ ΔR_{20} measurement during the dosage-step tests BWa and BWb in Odriozola et al. [17]. Table S21 summarises the parameter estimation procedure and Table S22 the optimal values and quality of the estimators. The quality of the parameters was worse than for the AnMBR models, especially for the deposition related parameters, that is: 74% of the deposition related and 40% SCR related parameters had poor quality. This was partially caused by the high pairwise correlation between parameters (>0.50) producing an ill-conditioned optimisation problem [20]. The parameter $\varepsilon_{\mathrm{c0}}$ was of good quality because it was estimated separately for most models, namely $\theta_{II} = \{\varepsilon_{\mathrm{c0}}\}$, except for D2 $\alpha_{2,p}$ and D2 α_2 where $\theta_{II} = \{\varepsilon_{\mathrm{c0}}, C_d\}$. $\varepsilon_{\mathrm{c0}}$ was estimated separately because it was the most influential parameter in each model and most subsets containing $\varepsilon_{\mathrm{c0}}$ had a collinearity index above the threshold of 10.

The calibration procedure can be improved by adding identifiability steps or improving the dataset used for calibration. For example, adding a second identifiability step with θ_{III}, decreased the parameters with poor quality from 74% to 38% for the deposition parameters and from 40% to 17% for the SCR parameters (results not shown) and identical or similar predictions of the validation data was obtained with one and two identifiability steps, Figure S28. The only models, of which predictions with one and two identifiability steps differed, were D1c $\alpha_{c,1}$, D2 $\alpha_{c,1}$, D3 $\alpha_{c,1}$, D3c $\alpha_{c,3}$, and D3c $\alpha_{c,3p}$; where for D1c $\alpha_{c,1}$ the prediction was considerably improved by adding a second identifiability analysis, whereas for the remaining models the prediction was only slightly worsen. Therefore, the calibration procedure was improved by adding a second identifiability step because it decreased the relative error of the parameters without deteriorating the model prediction capacity substantially or even improving it. Further adding extra identifiability steps was not analysed in this research.

Figure S25 and Figure S26 compare the experimental ΔR_{20} with the predicted values using the optimal parameters for each model, which were obtained with the original calibration procedure with one identifiability step. Most of the models that included a SCR submodel with cake compression ($\alpha_{\mathrm{c},jp}$) could not be calibrated to satisfactorily predict the experimental data. The exceptions were D1c $\alpha_{\mathrm{c},2p}$ and D3 with $\alpha_{\mathrm{c},1p}$, $\alpha_{\mathrm{c},2p}$, and $\alpha_{\mathrm{c},3p}$. Although D1c $\alpha_{\mathrm{c},2p}$ approximated the experimental ΔR_{20} used for calibration, the model failed at high c_{X} during model validation. For models with D3, the predictions with the compression submodels $\alpha_{\mathrm{c},ip}$ were identical to the corresponding non-compressed submodel $\alpha_{\mathrm{c},i}$ for i = [1,2,3]. This can be attributed to the fact that the estimated $f_{\mathrm{X},c}$ was considerably low, causing negligible deposition of particulate material (ω_{X}), and thus,the compressed SCR approximated the SCR without compression. Therefore, the models that coupled D3 with $\alpha_{\mathrm{c},1p}$, $\alpha_{\mathrm{c},2p}$, or $\alpha_{\mathrm{c},3p}$ in fact, did not describe a compressible cake.

Regarding the alternate AnDFCm models that included a non-compressible SCR submodel different observations were found. The models that included $\alpha_{\mathrm{c},1}$ predicted the experimental ΔR_{20} reasonably well for the three datasets used during calibration. In addition, these models behaved similar for all deposition submodels (i.e. D1c, D2 and D3). For models including $\alpha_{\mathrm{c},2}$ or $\alpha_{\mathrm{c},3}$, D1c $\alpha_{\mathrm{c},2}$ and D3 $\alpha_{\mathrm{c},2}$ behaved similarly whereas D2 $\alpha_{\mathrm{c},2}$ differed; and analogous D1c $\alpha_{\mathrm{c},3}$ and D3 $\alpha_{\mathrm{c},3}$ behaved similarly and D2 $\alpha_{\mathrm{c},3}$ differed. D2 $\alpha_{\mathrm{c},2}$ predicted the experimental ΔR_{20} better than D1c $\alpha_{\mathrm{c},2}$ and D3 $\alpha_{\mathrm{c},2}$; whereas D2 $\alpha_{\mathrm{c},3}$ predicted the experimental ΔR_{20} worse for D1c $\alpha_{\mathrm{c},3}$ and D3 $\alpha_{\mathrm{c},3}$.

Global sensitivity analysis results

Table S20. Sensitivity analysis of alternate AnDFCm filtration models: standardised regression coefficients (β_k) and mean of the standardised regression coefficients with R^2>0.7 ($\overline{\beta_k}$). Influential parameters with absolute β_k or $\overline{\beta_k}$ value above 0.10 (*).

Model	R^2	$\overline{R^2}$	β_k ($\overline{\beta_k}$)							
			C_d	$f_{\mathrm{C,c}}$	$f_{\mathrm{X,c}}$	h_t	$K_{S,\mathrm{c}}$	$q_{m,\mathrm{MS}}$	β_{ST}	γ
D1c $\alpha_{\mathrm{c},1}$	0.92	0.92					-0.09	0.10*		
D1c $\alpha_{\mathrm{c},1p}$	0.06	0.84					0.02 (0.12*)	0.004 (-0.12*)		
D1c $\alpha_{\mathrm{c},2}$	0.95	0.92					-0.11*	0.07		
D1c $\alpha_{\mathrm{c},2p}$	0.02	ND [a]					ND [a]	ND [a]		
D1c $\alpha_{\mathrm{c},3}$	0.98	0.98					-0.27*	0.25*		
D1c $\alpha_{\mathrm{c},3p}$	0.02	0.93					0.04 (0.27*)	-0.06 (-0.28*)		
D1c $\alpha_{\mathrm{c},4p}$	0.00	0.98					0.01 (0.40*)	-0.05 (-0.41*)		

[a] Cannot be determined because all R^2<0.7. The subset used for parameter estimation was the one from D1c $\alpha_{\mathrm{c},2}$. (continued)

Table S20 continued. Sensitivity analysis of alternate AnDFCm filtration models: standardised regression coefficients (β_k) and mean of the standardised regression coefficients with R^2>0.7 ($\overline{\beta_k}$). Influential parameters with absolute β_k or $\overline{\beta_k}$ value above 0.10 (*).

Model	R^2	$\overline{R^2}$	β_k ($\overline{\beta_k}$)							
			C_d	$f_{\mathrm{C,c}}$	$f_{\mathrm{X,c}}$	h_t	$K_{S,\mathrm{c}}$	$q_{m,\mathrm{MS}}$	β_{ST}	γ
D2 $\alpha_{\mathrm{c},1}$	0.90	0.91	0.14*	-0.03		-0.02			0.14*	0.01
D2 $\alpha_{\mathrm{c},1p}$	0.89	0.89	0.12*	-0.06		-0.02			0.18*	-0.01
D2 $\alpha_{\mathrm{c},2}$	0.90	0.90	0.13*	-0.06		-0.002			0.18*	-0.005
D2 $\alpha_{\mathrm{c},2p}$	0.89	0.90	0.12*	-0.05		0.04			0.23*	0.02
D2 $\alpha_{\mathrm{c},3}$	0.98	0.95	0.48*	-0.01		0.01			0.50*	0.0001
D2 $\alpha_{\mathrm{c},3p}$	0.97	0.95	0.49*	-0.01		-0.01			0.52*	0.01
D2 $\alpha_{\mathrm{c},4p}$	0.98	0.95	0.58*	-0.02		0.01			0.62*	0.01
D3 $\alpha_{\mathrm{c},1}$	0.93	0.91		0.004	-0.18*					
D3 $\alpha_{\mathrm{c},1p}$	0.93	0.91		-0.01	-0.19*					
D3 $\alpha_{\mathrm{c},2}$	0.11	0.92		0.02 (0.01)	0.08 (0.20*)					
D3 $\alpha_{\mathrm{c},2p}$	0.21	0.92		-0.06 (-0.01)	-0.03 (0.18*)					
D3 $\alpha_{\mathrm{c},3}$	0.97	0.96		-0.56*	0.09					
D3 $\alpha_{\mathrm{c},3p}$	0.97	0.96		-0.55*	0.10*					
D3 $\alpha_{\mathrm{c},4p}$	0.01	0.91		0.03 (0.12*)	0.06 (0.62*)					

(continued)

Table S20 continued. Sensitivity analysis of alternate AnDFCm filtration models: standardised regression coefficients (β_k) and mean of the standardised regression coefficients with R^2>0.7 ($\overline{\beta_k}$). Influential parameters with absolute β_k or $\overline{\beta_k}$ value above 0.10 (*).

Model	β_k ($\overline{\beta_k}$)					
	k_c	k_{CK}	$\varepsilon_{\mathrm{c0}}$	ρ_{C}	ρ_{X}	P_a
D1c $\alpha_{\mathrm{c},1}$		-0.19*	0.93*	0.01	0.18*	
D1c $\alpha_{\mathrm{c},1p}$		0.07 (0.19*)	-0.26* (-0.87*)	-0.001 (0.02)	0.03 (-0.20*)	0.01 (-0.03)
D1c $\alpha_{\mathrm{c},2}$	-0.18*		0.92*	0.03	0.19*	
D2 $\alpha_{\mathrm{c},1}$		-0.16*	0.88*	0.05	0.13*	
D2 $\alpha_{\mathrm{c},1p}$		-0.19*	0.87*	0.03	0.15*	
D2 $\alpha_{\mathrm{c},2}$	-0.18*		0.89*	0.04	0.14*	-0.18*
D2 $\alpha_{\mathrm{c},2p}$	-0.18*		0.89*	0.04	0.17*	-0.18*
D3 $\alpha_{\mathrm{c},1}$		-0.18*	0.92*	0.02	0.18*	
D3 $\alpha_{\mathrm{c},1p}$		-0.17*	0.90*	0.01	0.18*	-0.003
D3 $\alpha_{\mathrm{c},2}$	-0.01 (0.17*)		0.31* (-0.89*)	0.01 (-0.01)	-0.12* (-0.22*)	
D3 $\alpha_{\mathrm{c},2p}$	-0.05 (0.18*)		0.46* (-0.92*)	0.04 (0.005)	0.03 (-0.18*)	0.02 (0.01)

(continued)

Table S20 continued. Sensitivity analysis of alternate AnDFCm filtration models: standardised regression coefficients (β_k) and mean of the standardised regression coefficients with R^2>0.7 ($\overline{\beta_k}$). Influential parameters with absolute β_k or $\overline{\beta_k}$ value above 0.10 (*).

Model	β_k ($\overline{\beta_k}$)					
	P_a	P_b	ζ_1	ζ_2	ζ_3	ζ_4
D1c $\alpha_{\mathrm{c},3}$		-0.52*	-0.08	-0.44*	-0.61*	0.11*
D1c $\alpha_{\mathrm{c},3p}$	-0.07 (-0.04)	0.07 (0.54*)	-0.03 (0.50*)	0.12* (0.04)	-1×10^{-5} (0.23*)	0.01 (-0.07)
D1c $\alpha_{\mathrm{c},4p}$			-0.04 (0.64*)	-0.06 (0.15*)	0.01 (0.06)	-0.02 (-0.01)
D2 $\alpha_{\mathrm{c},3}$		-0.49*	-0.04	-0.46*	-0.01	0.004
D2 $\alpha_{\mathrm{c},3p}$	-0.003	-0.52*	-0.04	-0.48*	-0.003	-0.003
D2 $\alpha_{\mathrm{c},4p}$			-0.05	-0.56*	-0.01	-0.002
D3 $\alpha_{\mathrm{c},3}$		-0.48*	-0.08	-0.39*	-0.56*	0.10*
D3 $\alpha_{\mathrm{c},3p}$	-0.02	-0.44*	-0.06	-0.37*	-0.57*	0.11*
D3 $\alpha_{\mathrm{c},4p}$			-0.03 (0.08)	-0.07 (0.67*)	0.002 (0.11*)	0.06 (-0.03)

Parameter estimation results

Table S21. Parameter estimation procedure for the alternate AnDFCm filtration models. Parameters contained in subset θ_{II} and θ_{II}, number of pairwise correlations above 0.50 (nCorr) and root mean square error (RMSE) at optimal parameter values.

Model	Subset θ_{II}			Subset θ_{III}		
	Parameters	nCorr	RMSE	Parameters	nCorr	RMSE
D1c $\alpha_{c,1}$	$\{\varepsilon_{c0}\}$	NA	2.8	$\{k_{CK}, \rho_X, q_{m,MS}\}$	2	2.1
D1c $\alpha_{c,1p}$	$\{\varepsilon_{c0}\}$	NA	5.3	$\{k_{CK}, \rho_X, q_{m,MS}, K_{S,c}\}$	4	4.9
D1c $\alpha_{c,2}$	$\{\varepsilon_{c0}\}$	NA	5.6	$\{k_c, \rho_X, K_{S,c}\}$	3	4.0
D1c $\alpha_{c,2p}$	$\{\varepsilon_{c0}\}$	NA	3.8	$\{k_c, \rho_X, K_{S,c}\}$	3	2.4
D1c $\alpha_{c,3}$	$\{\zeta_3, \zeta_2, q_{m,MS}\}$	1	1.7	$\{K_{S,c}, P_b, \zeta_4\}$	ND[a]	1.6
D1c $\alpha_{c,3p}$	$\{P_b, \zeta_1, \zeta_3\}$	3	3.6	$\{q_{m,MS}, K_{S,c}\}$	1	3.6
D1c $\alpha_{c,4p}$	$\{\zeta_1, \zeta_2\}$	1	8.4	$\{q_{m,MS}, K_{S,c}\}$	1	8.4
D2 $\alpha_{c,1}$	$\{\varepsilon_{c0}\}$	NA	7.0	$\{\beta_{ST}, C_d, k_{CK}, \rho_X\}$	6	1.9
D2 $\alpha_{c,1p}$	$\{\varepsilon_{c0}\}$	NA	7.5	$\{\beta_{ST}, C_d, k_{CK}, \rho_X\}$	6	5.5
D2 $\alpha_{c,2}$	$\{\varepsilon_{c0}, C_d\}$	1	2.1	$\{\beta_{ST}, k_c, \rho_X\}$	1	2.1
D2 $\alpha_{c,2p}$	$\{\varepsilon_{c0}, C_d\}$	1	4.0	$\{\beta_{ST}, k_c, \rho_X\}$	2	4.0
D2 $\alpha_{c,3}$	$\{\beta_{ST}, C_d\}$	0	8.4	$\{P_b, \zeta_2\}$	1	8.4
D2 $\alpha_{c,3p}$	$\{\beta_{ST}, C_d\}$	1	8.4	$\{P_b, \zeta_2\}$	1	6.3
D2 $\alpha_{c,4p}$	$\{\beta_{ST}, C_d\}$	1	8.4	$\{\zeta_2\}$	NA	7.1
D3 $\alpha_{c,1}$	$\{\varepsilon_{c0}\}$	NA	2.4	$\{k_{CK}, \rho_X, f_X\}$	3	2.0
D3 $\alpha_{c,1p}$	$\{\varepsilon_{c0}\}$	NA	2.4	$\{k_{CK}, \rho_X, f_X\}$	3	2.0
D3 $\alpha_{c,2}$	$\{\varepsilon_{c0}\}$	NA	5.3	$\{k_c, \rho_X, f_X\}$	3	3.3
D3 $\alpha_{c,2p}$	$\{\varepsilon_{c0}\}$	NA	5.3	$\{k_c, \rho_X, f_X\}$	3	3.3
D3 $\alpha_{c,3}$	$\{P_b, \zeta_2, \zeta_3\}$	1	1.6	$\{f_C, \zeta_4\}$	1	1.3
D3 $\alpha_{c,3p}$	$\{P_b, \zeta_2, \zeta_3\}$	1	1.6	$\{f_X, f_C, \zeta_4\}$	3	1.2
D3 $\alpha_{c,4p}$	$\{f_X, \zeta_2\}$	1	8.6	$\{f_C, \zeta_3\}$	1	8.6

NA: not applicable

[a] Could not be determined due to numerical error: the Jacobian was zero for all values in at least one parameter resulting in division by zero when calculating the covariance matrix.

Table S22. Optimised parameter values ($\hat{\theta}$) for the alternate AnDFCm filtration models. Relative error $\sigma_\theta/\hat{\theta}$ shown between brackets. θ^o is the initial guess (nominal values).

↓Model	C_d	$f_{\mathrm{C,c}}$	$f_{\mathrm{X,c}}$	β_{ST}	$K_{S,\mathrm{c}}$	$q_{m,\mathrm{MS}}$
Units→	-	-	-	-	kg	-
θ^o→	0.40	0.25	0.25	1.75×10^{-4}	0.2	4.71
D1c $\alpha_{\mathrm{c},1}$						2.2×10^{-14} [1×10^{12}]
D1c $\alpha_{\mathrm{c},1p}$					1.01 [1×10^{4}]	2.71 [8×10^{3}]
D1c $\alpha_{\mathrm{c},2}$					0.08 [684]	
D1c $\alpha_{\mathrm{c},2p}$					0.05 [0.001]	
D1c $\alpha_{\mathrm{c},3}$					0.21 [4×10^{9}]	3.3×10^{-12} [5×10^{9}]
D1c $\alpha_{\mathrm{c},3p}$					5.7×10^{10} [1×10^{5}]	1.6×10^{11} [1×10^{5}]
D1c $\alpha_{\mathrm{c},4p}$					6.8×10^{-4} [3.78]	0.02 [2.63]
D2 $\alpha_{\mathrm{c},1}$	0.003 [4.3]			1×10^{-5} [33.5]		
D2 $\alpha_{\mathrm{c},1p}$	0.48 [545]			1×10^{-4} [1.10]		
D2 $\alpha_{\mathrm{c},2}$	0.10 [0.16]			2×10^{-4} [1.23]		
D2 $\alpha_{\mathrm{c},2p}$	0.19 [0.03]			2×10^{-4} [0.02]		
D2 $\alpha_{\mathrm{c},3}$	0.05 [7.9×10^{3}]			0.50 [7.25]		
D2 $\alpha_{\mathrm{c},3p}$	9.46 [44.5]			0.02 [296]		
D2 $\alpha_{\mathrm{c},4p}$	4.59 [0.19]			0.39 [0.51]		
D3 $\alpha_{\mathrm{c},1}$			0.06 [2×10^{6}]			
D3 $\alpha_{\mathrm{c},1p}$			0.06 [3×10^{6}]			
D3 $\alpha_{\mathrm{c},2}$			0.04 [1×10^{6}]			
D3 $\alpha_{\mathrm{c},2p}$			0.04 [1×10^{6}]			
D3 $\alpha_{\mathrm{c},3}$		0.13 [0.23]				
D3 $\alpha_{\mathrm{c},3p}$		0.12 [0.22]	0.28 [0.07]			
D3 $\alpha_{\mathrm{c},4p}$		0.24 [2×10^{5}]	0.21 [0.25]			

(continued)

Table S22 continued. Optimised parameter values ($\hat{\theta}$) for the alternate AnDFCm filtration models. Relative error $\sigma_\theta/\hat{\theta}$ shown between brackets. θ^o is the initial guess (nominal values).

↓Model	k_c	k_{CK}	$\varepsilon_{\mathrm{c0}}$	ρ_{X}
Units→	$\times 10^{17}$ m^{-2}	$\times 10^{7}$	-	$\times 10^{3}$ kg m^{-3}
θ^o→	1.0	4.0	0.66	1.24
D1c $\alpha_{c,1}$		0.94 [0.32]	0.33 [0.02]	4.67 [0.38]
D1c $\alpha_{c,1p}$		4.81 [186]	0.62 [0.003]	4.83 [3.61]
D1c $\alpha_{c,2}$	12.91 [342]		0.44 [0.05]	15.3 [0.26]
D1c $\alpha_{c,2p}$	8.05 [0.15]		0.66 [0.002]	5.63 [0.18]
D2 $\alpha_{c,1}$		0.69 [33.2]	0.15 [0.04]	6.7 [0.98]
D2 $\alpha_{c,1p}$		5.7 [0.75]	0.21 [0.01]	0.54 [524]
D2 $\alpha_{c,2}$	1.01 [1.27]		0.11 [0.02]	1.22 [0.17]
D2 $\alpha_{c,2p}$	1.00 [0.02]		0.19 [0.004]	1.24 [0.06]
D3 $\alpha_{c,1}$		21.1 [0.39]	0.47 [0.02]	2.05 [3×10^{6}]
D3 $\alpha_{c,1p}$		21.1 [0.39]	0.47 [0.02]	2.05 [3×10^{6}]
D3 $\alpha_{c,2}$	13.7 [0.35]		0.60 [0.04]	4.57 [1×10^{6}]
D3 $\alpha_{c,2p}$	13.7 [0.35]		0.60 [0.04]	4.57 [1×10^{6}]

(continued)

Table S22 continued. Optimised parameter values ($\hat{\theta}$) for the alternate AnDFCm filtration models. Relative error $\sigma_\theta/\hat{\theta}$ shown between brackets. θ^o is the initial guess (nominal values).

↓Model	P_{b}	ζ_1	ζ_2	ζ_3	ζ_4
Units→	$\times 10^{3}$ Pa	$\times 10^{3}$	$\times 10^{4}$	-	-
θ^o→	4.1799	1.16	1.36	172.4	150.9
D1c $\alpha_{c,3}$	4.2 [∞^a]		1.57 [0.23]	248.7 [0.04]	142.4 [∞^a]
D1c $\alpha_{c,3p}$	0.85 [0.49]	73.4 [0.58]		169.6 [0.18]	
D1c $\alpha_{c,4p}$		3.69 [0.09]	0.56 [0.51]		
D2 $\alpha_{c,3}$	1.9×10^{7} [0.87]		0.63 [1.05]		
D2 $\alpha_{c,3p}$	9401 [6×10^{5}]		570 [6×10^{5}]		
D2 $\alpha_{c,4p}$			7.7×10^{5} [0.01]		
D3 $\alpha_{c,3}$	27.9 [0.28]		0.77 [0.32]	236.9 [0.05]	12.4 [0.69]
D3 $\alpha_{c,3p}$	27.9 [0.28]		0.77 [0.32]	236.9 [0.05]	7.60 [0.55]
D3 $\alpha_{c,4p}$			1.72 [2.54]	178 [2×10^{5}]	

[a] The Jacobian was zero for all values in at least one parameter resulting in division by zero when calculating the covariance matrix.

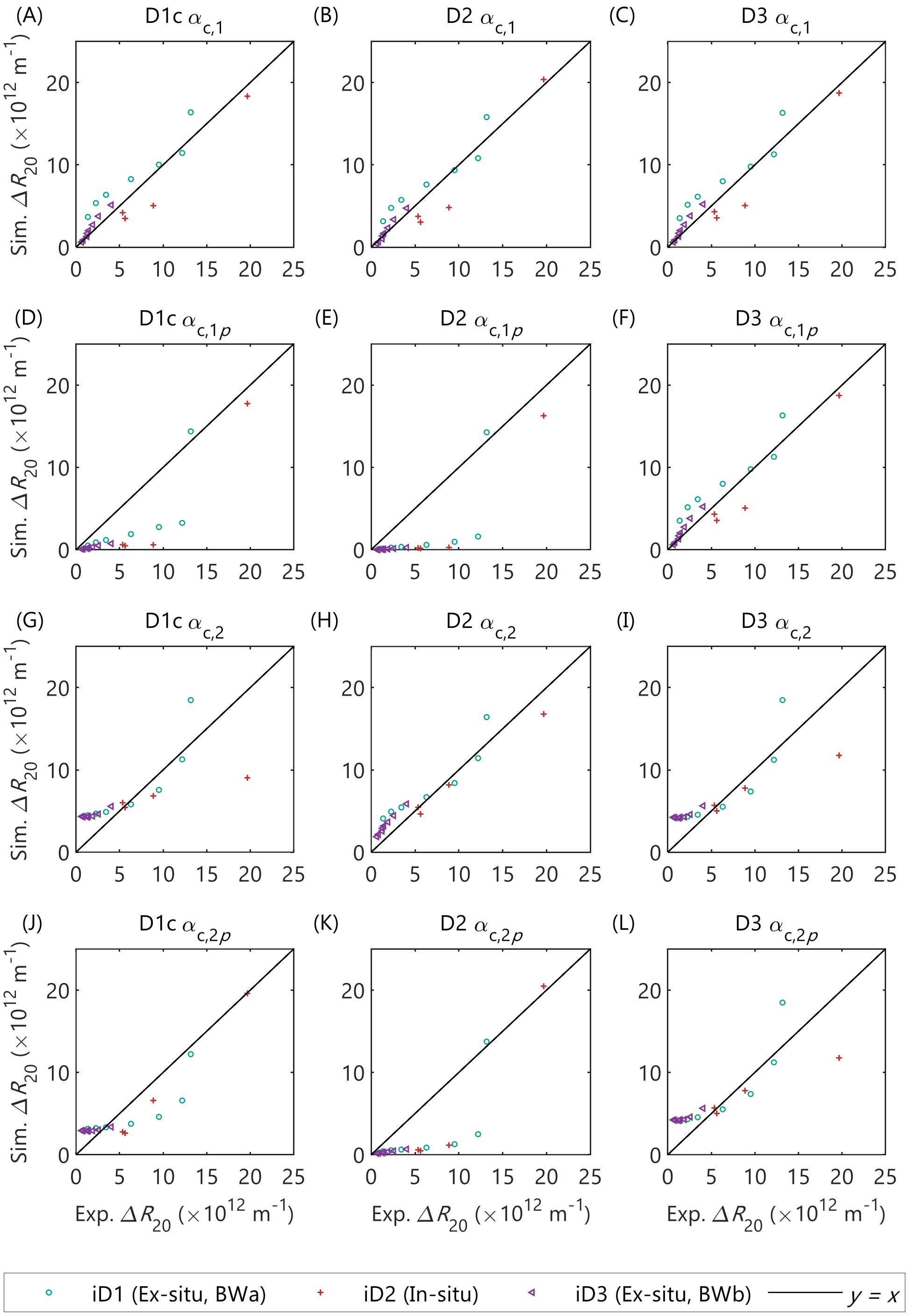

Figure S25. Calibration of the alternate AnDFCm filtration models combining the different deposition submodels (D1c, D2 and D3) with the Carman-Kozeny based specific cake resistance submodels ($\alpha_{c,1}$, $\alpha_{c,1p}$, $\alpha_{c,2}$ and $\alpha_{c,2p}$). Calibration performed using ΔR_{20} measurement during in-situ and ex-situ flux enhancer additions to the reactor and to grab samples from the pilot AnMBR, respectively.

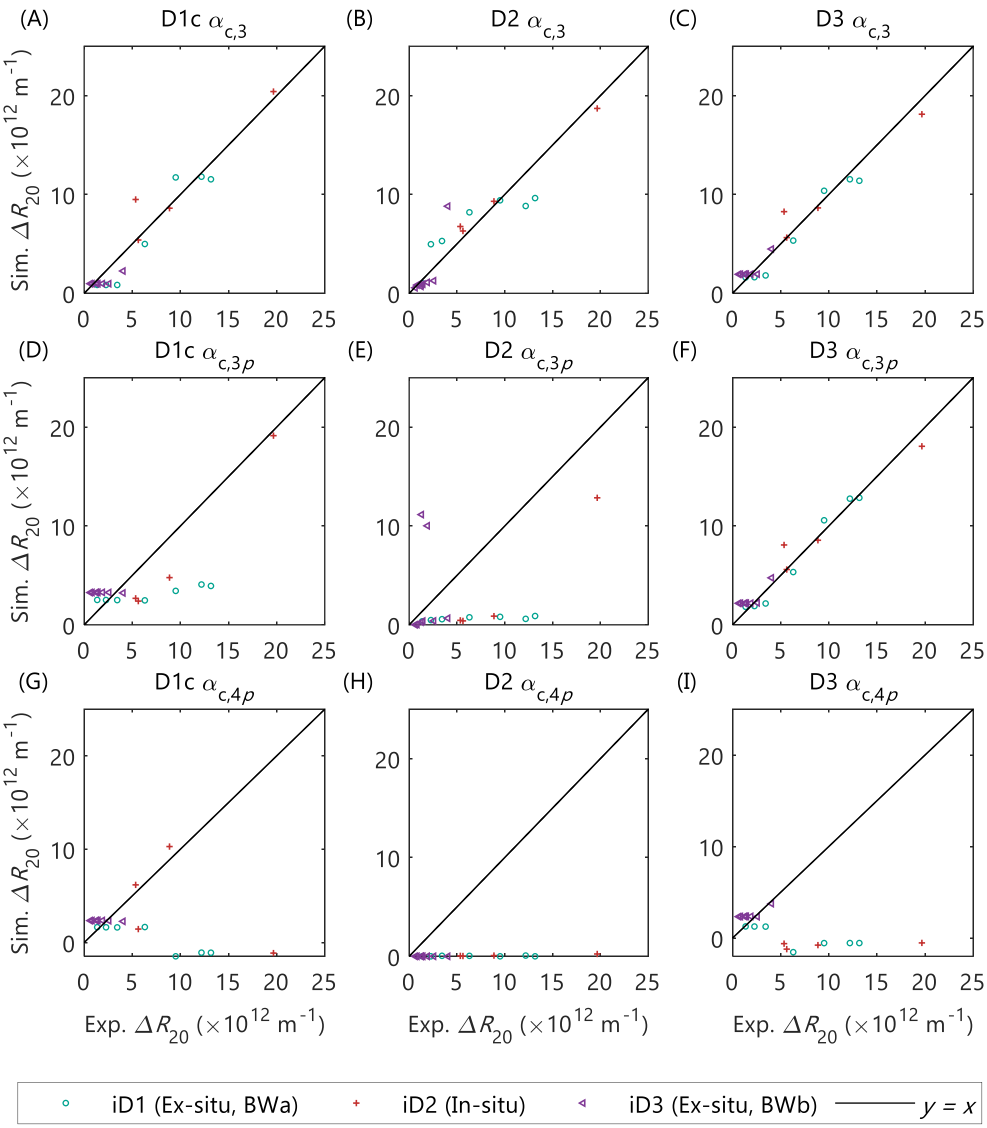

Figure S26. Calibration of the alternate AnDFCm filtration models combining the different deposition submodels (D1c, D2 and D3) with the specific cake resistance submodels based on the empirical equation by Cho et al. [22] ($\alpha_{c,3}$, $\alpha_{c,3p}$, and $\alpha_{c,4p}$). Calibration performed using ΔR_{20} measurement during in-situ and ex-situ flux enhancer additions to the reactor and to grab samples from the pilot AnMBR, respectively.

Long-term prediction with calibrated models

Table S23. Statistical indicators representing model accuracy of the alternate AnDFCm filtration models for long-term ΔR_{20} prediction in the pilot AnMBR. Normalised root mean square error (nRMSE), Pearson correlation coefficient (Corr), and mean absolute percentage error (MAPE).

Model	nRMSE	Corr	MAPE
D1c $\alpha_{c,1}$	0.29	0.74	23
D1c $\alpha_{c,1p}$	0.96	0.28	80
D1c $\alpha_{c,2}$	0.33	0.65	21
D1c $\alpha_{c,2p}$	4.38	0.09	149
D1c $\alpha_{c,3}$	0.32	0.69	31
D1c $\alpha_{c,3p}$	0.63	0.55	56
D1c $\alpha_{c,4p}$	1.21	-0.29	95
D2 $\alpha_{c,1}$	0.33	0.73	25
D2 $\alpha_{c,1p}$	0.95	0.36	89
D2 $\alpha_{c,2}$	0.24	0.82	15
D2 $\alpha_{c,2p}$	1.56	0.43	95
D2 $\alpha_{c,3}$	0.35	0.58	27
D2 $\alpha_{c,3p}$	0.94	0.37	88
D2 $\alpha_{c,4p}$	1.06	0.26	99
D3 $\alpha_{c,1}$	0.26	0.78	22
D3 $\alpha_{c,1p}$	0.26	0.78	22
D3 $\alpha_{c,2}$	0.25	0.80	15
D3 $\alpha_{c,2p}$	0.25	0.80	15
D3 $\alpha_{c,3}$	0.25	0.78	20
D3 $\alpha_{c,3p}$	0.21	0.84	17
D3 $\alpha_{c,4p}$	1.11	-0.24	101

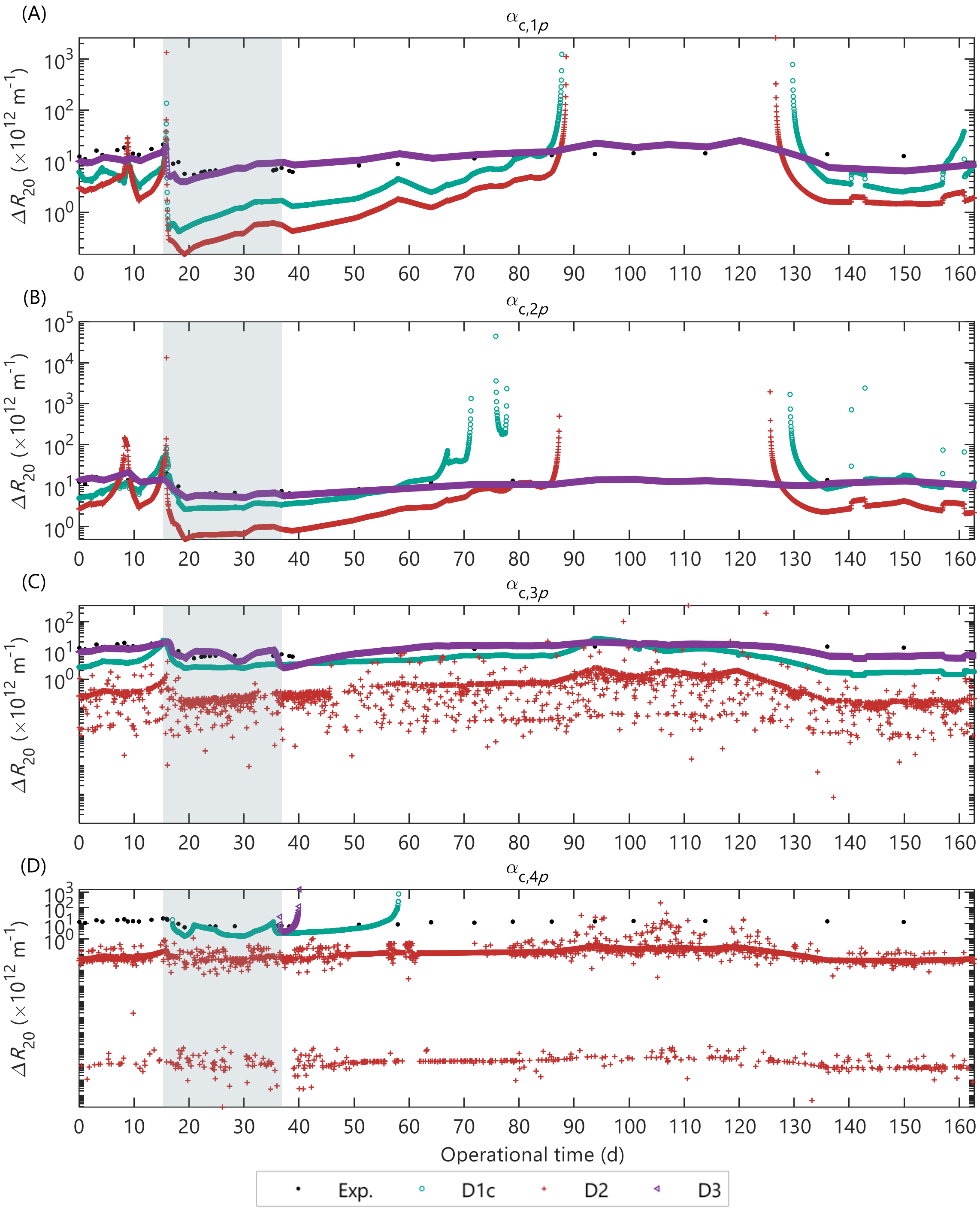

Figure S27. Validation of the alternate AnDFCm filtration models that combine the different deposition submodels (D1c, D2 and D3) with the compressible specific cake resistance submodels: (A) α_{1p}, (B) α_{2p}, (C) α_{3p}, and (D) α_{4p}. The grey area represent the in-situ data used for model calibration.

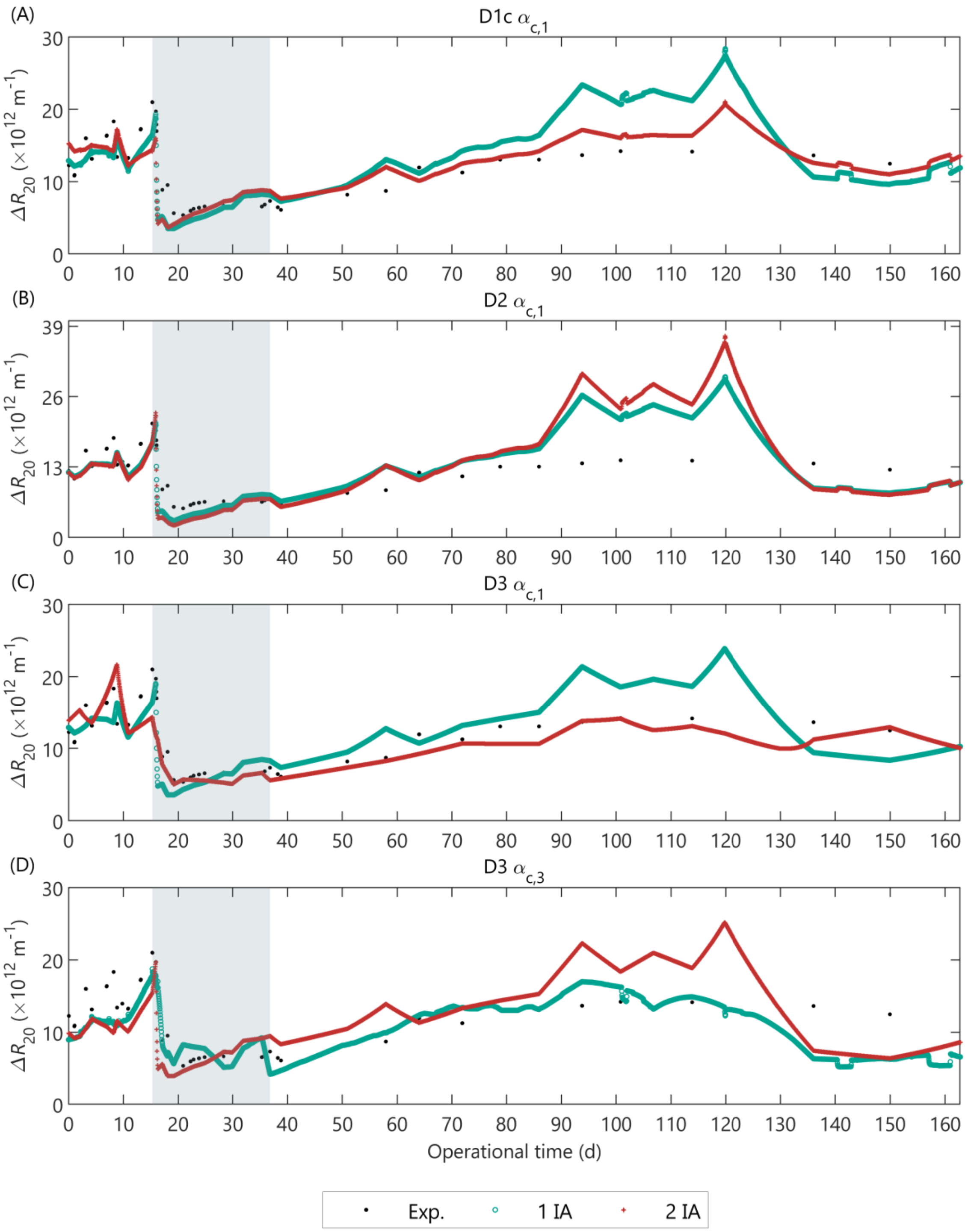

Figure S28. Comparison of alternate AnDFCm filtration models calibrated using one (1 IA) and two (2 IA) steps of identifiability analysis. Only the alternate models with different predictions for 1 IA and 2 IA are shown, as follows: (A) D1c α_1, (B) D2 α_1, (C) D3 α_1, and (D) D3c $\alpha_3 \equiv$ D3c α_{3p}. The remaining models combining the deposition submodels D1c, D2 and D3 with specific cake resistance submodels α_1, α_2, α_3, α_{1p}, α_{2p}, α_{3p} and α_{4p} presented identical predictions for 1 IA and 2 IA. The grey area represent the in-situ data used for model calibration.

S12. Effect of sludge characteristics on filterability predictions

Figure S29 show the effect of the concentrations of colloidal and particulate material on the predicted ΔR_{20} with the alternate calibrated AnDFCm filtration models. The sludge characteristics were varied inside the simulation ranges in Table S24, which were based on the observed ranges in the pilot AnMBR. The remaining variables were set to the nominal values presented in the table, for example, all the ΔR_{20} simulations in Figure S29 were done at T = 296 K and d_p = 2.7×10^5 m.

Table S24. Nominal values and simulation range of operational conditions and sludge characteristics.

	Variable	Units	Nominal value [a]	Range in pilot AnMBR	Simulation range
c_C	Concentration of colloidal material	Kg m^{-3}	0.35	[0.14, 0.62]	[0.10, 0.65]
c_X	Concentration of particulate material	Kg m^{-3}	9.25	[4.9, 15.9]	[4.0, 16.0]
d_p	Mean particle diameter	$\times10^5$ m	2.7	[2.1, 4.5]	[2.0, 5.0]
T	Sludge temperature	K	296	[292, 301]	NA

[a] Mean value during pilot AnMBR operation.

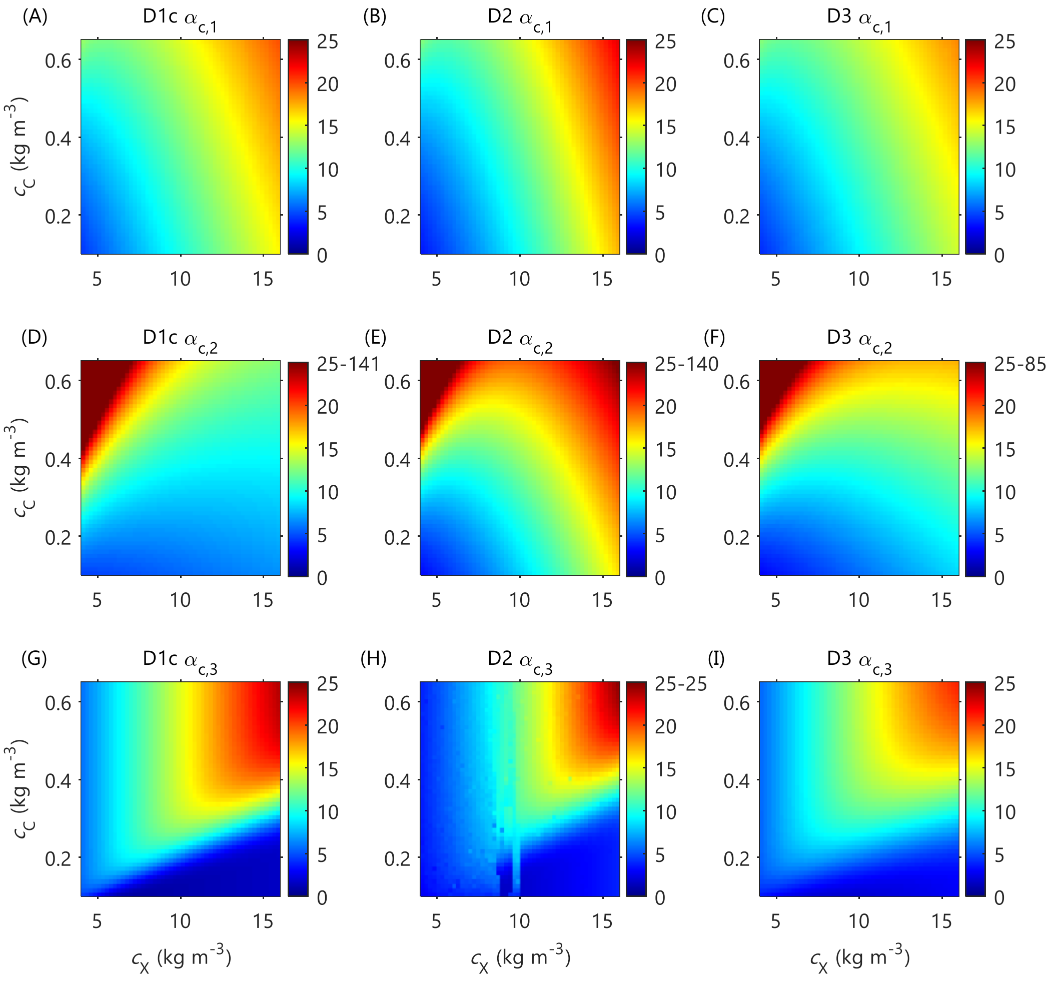

Figure S29. Effect of the concentrations of colloidal material (c_C) and particulate material (c_X) on the predicted ΔR_{20} ($\times10^{-12}$ m^{-1}) with different calibrated AnDFCm filtration models: (A) D1c $\alpha_{c,1}$, (B) D2 $\alpha_{c,1}$, (C) D3 $\alpha_{c,1}$, (D) D1c $\alpha_{c,2}$, (E) D2 $\alpha_{c,2}$, (F) D3 $\alpha_{c,2}$, (G) D1c $\alpha_{c,3}$, (H) D2 $\alpha_{c,3}$, and (I) D3 $\alpha_{c,3}$.

S13. Cake layer compression

Evenblij [23] presented the three possible hypothetical filtration curves obtained with the Delft filtration characterization method, and Geilvoet [6] related the filtration curves with a compressibility coefficient. The researchers qualified the cake layer by analysing the filtration curve ΔR versus ΔV_S obtained at constant flux, where ΔR is the additional resistance after production of a certain volume of permeate, and ΔV_S the volume of permeate produced per square meter of membrane surface area. Table S25 summarises the hypothetical filtration curves.

Table S25. Hypothetical filtration curves obtained with the Delft filtration characterization method [6,23].

Cake layer type	Exponential parameter b<br>$\Delta R = a\ \Delta {V_S}^b$	Compressibility coefficient s<br>$s = \frac{b-1}{b}$
Compressible	> 1	> 0
Linear	1	0
Equilibrium	< 1	< 0

We fitted the experimental resistance-volume curves obtained with the AnDFCm to the power equation presented in Table S25 and analysed the cake layer type based on the compressibility coefficient calculated with the optimised parameter b. Figure S30 shows the histogram of compressibility coefficients obtained for the in-situ ΔR_{20} measurement performed in the pilot AnMBR; the compressibility coefficient varied between -0.39 and 0.19. In accordance with Table S25, 34% of the filtration curves corresponded to a linear cake layer ($s = 0$), 48% to an equilibrium cake layer ($s < 0$), and 18% to a compressible cake layer ($s > 0$). Furthermore, the grab sludge samples from different AnMBRs displayed in Figure S31 generated a linear or equilibrium cake layer. Therefore, results suggested that the cake layer formed with the AnDFCm when filtering anaerobic sludge samples from AnMBR is none-compressible or slightly compressible.

For aerobic sludge samples, from MBRs, the cake layer formed using the DFCm was hardly compressible, the compression coefficient varied between 0-0.3 [24]. The DFCm (at 80 L m^{-2} h^{-1} and 1.0 m s^{-1}) and AnDFCm (at 60 L m^{-2} h^{-1} and 1.5 m s^{-1}) differed in the flux and crossflow velocities used during filtration; therefore, we cannot directly compare the numerical values of the compression coefficients. Nevertheless, the hypothetical filtration curves were valid for both measuring methods because they were performed at constant flux.

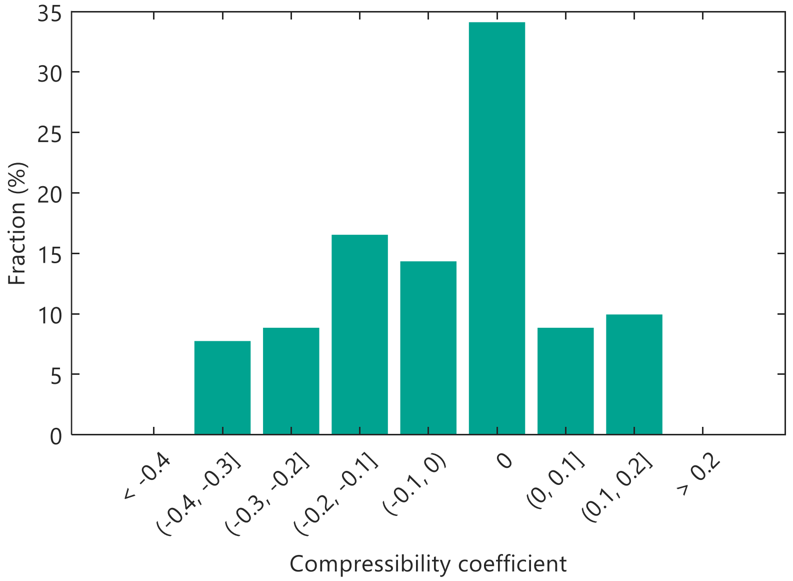

Figure S30. Distribution of the compressibility coefficient of the cake layer formed with the AnDFCm during in-situ ΔR_{20} measurements of the pilot AnMBR sludge.

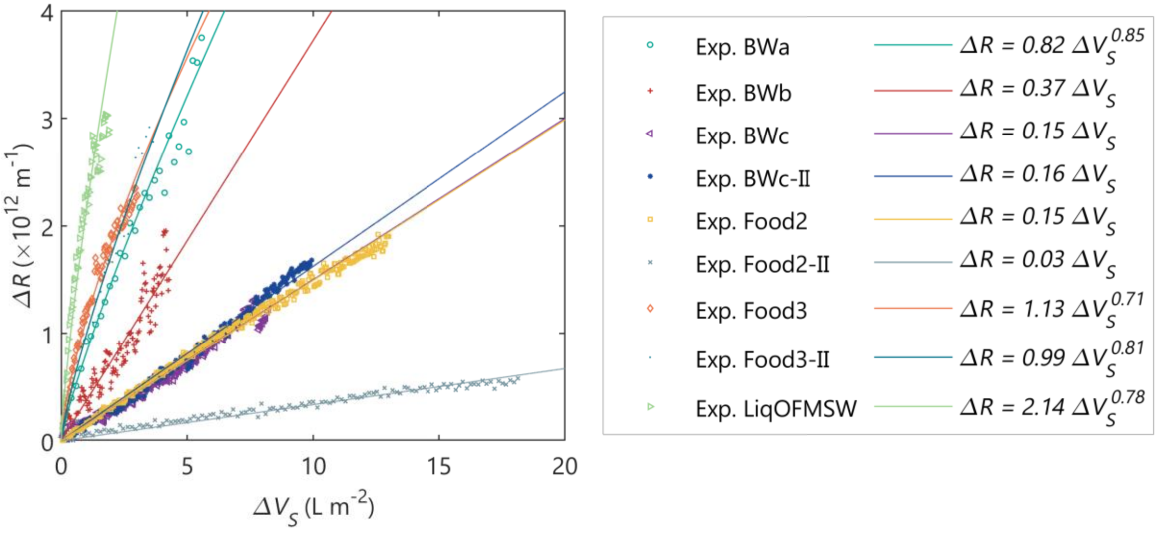

Figure S31. Added total resistance versus specific volume of permeate obtained when filtering grab sludge samples from different AnMBRs with the AnDFCm. Experimental data (markers) and fitted power curves (lines).

S14. Nomenclature

Abbreviations

AnDFCm	Anaerobic Delft filtration characterization method
AnMBR	Anaerobic membrane bioreactor
COD	Chemical oxygen demand
FE	Flux enhancer
FR	Fouling rate
HS	Humic substances
MBR	(aerobic) Membrane bioreactor
PBM	Population balance models
PSD	Particle size distribution
RIS	Resistance in series
RMSE	Root mean square error
SCR	Specific cake resistance
SMP	Soluble microbial products
SRC	Standardised regression coefficient
TMP	Transmembrane pressure
TSS	Total suspended solids
VFA	Volatile fatty acids

Symbols

α_{c}	Specific cake resistance (m kg^{-1})
$\alpha_{\mathrm{c},j}$	Specific cake resistance calculated with submodel j without cake compression (m kg^{-1})
$\alpha_{\mathrm{c},jp}$	Specific cake resistance calculated with submodel j with cake compression (m kg^{-1})
β_k	Standardised regression coefficient for parameter k
β_{ST}	Lumped parameter $\beta_{\mathrm{ST}} = \beta(1 - K_{\mathrm{ST}})$; β Erosion rate coefficient of the sludge cake; K_{ST} stickiness coefficient
γ	Compression coefficient for the dynamic cake layer (kg m^{-3})
γ_i	Empirical model parameter i
γ^k	Collinearity index of the parameter subset k
ΔR_{20}	Additional resistance when 20 L of permeate per m^2 of membrane surface are obtained during filtration at constant flux and crossflow velocity in the AnDFCm installation (m^{-1})
Δt_{fe}	Injection time of flux enhancer pulse-dosage (s)
ε_{c}	Cake layer porosity (-)

$\varepsilon_{\mathrm{c0}}$	Cake layer porosity without colloidal material (-)
ζ_i	Empirical model parameters
θ	Parameter subset for estimation
θ°	Initial guess
$\hat{\theta}$	Estimated parameters (optimal values)
θ_{F}	Total filtration time in one cycle (s)
θ_{R}	Total relaxation time in one cycle (s)
$\theta_{H,i}$	Temperature correction factor for Henry's law coefficient for component i (-)
θ_j	Temperature correction factor for the reaction rate of process j (-)
μ	Dynamic viscosity of the permeate at the operational temperature (Pa s)
μ_{20}	Dynamic viscosity of the permeate at 20°C (Pa s)
μ_i	Dynamic viscosity of fluid i (Pa s)
υ_{B}	Motor frequency of the blower (Hz)
υ_{P}	Motor frequency of the permeate pump (Hz)
$\upsilon_{i,j}$	Stoichiometric coefficients of component i in process j
ρ_j	Rate equations of process j (kgCOD m^{-3} d^{-1} or kmol m^{-3} d^{-1})
ρ_i	Density of component or fluid i (kg m^{-3})
σ_{θ}	Standard deviation of estimated parameters
ω_i	Mass of component i deposited per membrane area (kg m^{-2})
Alk_{BW}	Blackwater alkalinity (Kg$CaCO_3$ m^{-3})
A_{m}	Membrane surface area (m^2)
A_{MT}	Membrane tank cross-sectional area (m^2)
b_j	First order decay rate of microorganism in process j (d^{-1})
cCOD	Colloidal COD concentration (kgCOD m^{-3})
C_d	Drag coefficient (-)
C_i	Concentration of colloidal material i in the bulk liquid (kgCOD m^{-3})
c_i	Concentrations of component i in the liquid phase (kg m^{-3})
$c_{i,j}$	Concentration of component i in fluid j (kgCOD m^{-3} or kmol m^{-3})
c_{fe}	Total concentration of flux enhancer inside the reactor (kgCOD m^{-3})
csCOD	Submicron COD concentration (kgCOD m^{-3})
$csCOD_{BW}$	Submicron COD concentration in blackwater (kgCOD m^{-3})
D_{opt}	Optimal flux enhancer dosage (kg m^{-3})
d_p	Mean particle diameter (m)
$d_{p,i}$	Diameter of the i-th particle (m)
$d_{p,\mathrm{St}}$	Mean particle diameter at stable operation (m)
E_i	Input function of component i (kgCOD m^3 d^{-1})
f_{conv}	Conversion factor
$f_{i,\mathrm{c}}$	Fraction of material i depositied onto the membrane (-)

$f_{i,\mathrm{P}}$	Fraction of component i that passes through the membrane and reaches the permeate (-)
$f_{i,\mathrm{WS}}$	Fraction of component i that leaves the reactor with waste sludge flow (-)
$f_{\mathrm{objective}}$	Objective function for parameter estimation
$f_{p,i}$	Yield of product p on substrate i (-)
g	Gravitational acceleration (m s^{-2})
G	Apparent shear rate (s^{-1})
H_{MT}	Liquid level in membrane tank (m)
$i_{\mathrm{C},i}$	Carbon content of component i (kmole $kgCOD^{-1}$)
$i_{\mathrm{C,CXI,bio}}$	Colloidal fraction of the released suspended inert material upon biomass decay (-)
$i_{\mathrm{COD},i}$	Theoretical chemical oxygen demand for component i (kgCOD kg^{-1})
$I_{i,j}$	Inhibition factor of component i in process j (-)
$i_{i,\mathrm{CSInf}}$	Content of component i in the submicron material of the influent (kgCOD $kgCOD^{-1}$)
$i_{i,\mathrm{SInf}}$	Content of component i in the soluble material of the influent (kgCOD $kgCOD^{-1}$)
$i_{i,\mathrm{XInf}}$	Content of component i in the particulate material of the influent (kgCOD $kgCOD^{-1}$)
I_{MS}	Sigmoid inhibition function during membrane scouring (-)
$i_{\mathrm{N},i}$	Nitrogen content of component i (kmole $kgCOD^{-1}$)
J	Transmembrane flux (m^3 m^{-2} s^{-1})
J_{20}	20°C-normalised transmembrane flux (m^3 m^{-2} s^{-1})
$J_{20,\mathrm{AnDFCm}}$	20°C-normalised transmembrane flux applied in the AnDFCm installation (m^3 m^{-2} s^{-1})
J_{AnDFCm}	Transmembrane flux applied in the AnDFCm installation (m^3 m^{-2} s^{-1})
k_{ads}	Pseudo-first order reaction rate coefficient for flux enhancer adsorption (d^{-1})
k_c	Cake resistance coefficient (m^{-2})
k_{CK}	Carman-Kozeny cake resistance coefficient (-)
$k_{\mathrm{dis,bio}}$	First order reaction rate coefficient for biomass disintegration (d^{-1})
$K_{eq,\mathrm{fe},i}$	Equilibrium coefficient for flux enhancer and component i binding (kgCOD kg^{-1})
K_F	Parameter representing the fouling rate when J_{20} tends to zero (Pa s^{-1})
k_{floc}	Empirical flocculation-deflocculation rate (d^{-1})
$k_{\mathrm{floc,fe}}$	Flux enhancer induced flocculation yield (m $kgCOD^{-1}$ m^3)
$K_{H,i}$	Henry's law coefficient for component i (kgCOD m^{-3} bar^{-1} or kmol m^{-3} bar^{-1})
k_{hyd}	Unique first order reaction rate coefficient for all hydrolysis processes (d^{-1})
$k_{\mathrm{hyd},j}$	First order reaction rate coefficient for hydrolysis of component j (d^{-1})
$K_{I,i,j}$	Concentration of inhibitor i giving 50% inhibition on process j rate (kgCOD m^{-3})
k_j	Reaction rate for process j (d^{-1})
$K_{L,\mathrm{ads}}$	Langmuir affinity coefficient (m^3 $kgCOD^{-1}$)
$k_L a$	Dynamic gas-liquid transfer coefficient (d^{-1})
$k_{m,j}$	Monod maximum specific uptake rate for process j (d^{-1})
$K_{S,\mathrm{c}}$	Half-saturation coefficient for cake mass during membrane scouring (kg)

$K_{S,\mathrm{G}}$	Half-saturation coefficient for gas velocity during membrane scouring (m s^{-1})
$K_{S,\mathrm{IN}}$	Monod half saturation coefficient for inorganic nitrogen (kmol m^{-3})
$K_{S,j}$	Monod half saturation coefficient for process j (kgCOD m^{-3})
M_{fe}	Cumulative mass of flux enhancer (kgCOD)
$M_{\mathrm{fe,P}}$	Cumulative mass of flux enhancer removed with the permeate flow (kgCOD)
$\dot{m}_{\mathrm{fe}}$	Flux enhancer mass flow rate (kgCOD s^{-1})
$NH4_{BW}$	Ammonium nitrogen in blackwater (KgN m^{-3})
n_j	Numer of experimental observatons of the ouput variable j (-)
P_{a}	Pressure needed to double the specific resistance (Pa)
P_{b}	Transmembrane pressure coefficient (Pa)
pCOD	Permeate COD (kgCOD m^{-3})
p_{G}	Gas pressure in the headspace (bar)
P_i	Volume fraction (-)
$p_{i,\mathrm{G}}$	Partial pressure of gas i (bar)
$\mathrm{pH}_{\mathrm{LL},j}$	Lower pH limit where the group of organisms in process j is 50% inhibited.
$\mathrm{pH}_{\mathrm{UL},j}$	Upper pH limit where the group of organisms in process j is 50% inhibited.
Q_j	Volumetric flow rate of fluid j (m^3 s^{-1})
$q_{m,\mathrm{ads}}$	Maximum adsorption capacity corresponding to monolayer coverage (kgCOD kg^{-1})
$q_{m,\mathrm{MS}}$	Maximum membrane scouring velocity (-)
R^2	Coefficient of determination (-)
R_{c}	Cake layer resistance (m^{-1})
R_{m}	Membrane intrinsic resistance (m^{-1})
R_{t}	Total filtration resistance (m^{-1})
$S_{\mathrm{fe},e}$	Concentration of soluble flux enhancer in the bulk liquid after equilibrium (kgCOD m^{-3})
S_i	Concentration of soluble component i in the bulk liquid (kgCOD m^{-3} or kmol m^{-3})
t	Time (s)
T	Temperature (K or °C)
t_{conv}	Time conversion factor (86,400 s d^{-1})
tCOD	Total COD concentration (kgCOD m^{-3})
$tCOD_{BW}$	Total COD concentration in blackwater (kgCOD m^{-3})
t_{F}	Continuous filtration time in a cycle (s)
t_{fe0}	Initial time of flux enhancer pulse-dosage (s)
TMP	Transmembrane pressure (Pa)
$u_{\mathrm{L,AnDFCm}}$	Liquid crossflow velocity in the AnDFCm installation (m s^{-1})
u_{G}	Gas superficial velocity (in the AnMBR membrane tank) (m s^{-1})
V_{F}	Volume of permeate produced within the filtration time t_{F} (m^3 m^{-2})
V_{G}	Total gas volume (m^3)

V_L	Total mixed liquor volume (m^3)
V_P	Permeate volume produced (m^3)
V_T	Total reactor volume (m^3)
w_j	Weight of output variable j (-)
$X_{\mathrm{fe},e}$	Adsorbed concentration of flux enhancer after equilibrium (kgCOD m^{-3})
X_i	Concentration of particulate component i in the bulk liquid (kgCOD m^{-3})
$Y_{\mathrm{fe,C}}$	Yield of colloidal material flocculated per unit of flux enhancer adsorbed (kg kg^{-1})
$Y_{\mathrm{floc,fe}}$	Proportionality parameter between particle diameter and flux enhancer concentration (kg $kgCOD^{-1}$)
Y_j	Yield coefficient of biomass on substrate for process j.
$y_{e,j,i}$	i-th experimental value of the output variable j
$y_{m,j,i}$	i-th predicted value of the output variable j

Subscripts

aa	Amino acids / Amino acids degraders
ac	Acetate /Acetate degraders
bu	Butyrate
c	Cake layer
C	Colloidal
c4	Valerate and butyrate degraders
ch4	Methane
co2	Carbon dioxide
ch	Carbohydrates
fa	Long chain fatty acids (LCFA) / LCFA degraders
fe	Flux enhancer, cationic polymer
G	Gas
h2	Hydrogen / Hydrogen degraders
hs	Humic substances
I	Inert
IC	Inorganic carbon
IN	Inorganic nitrogen
Inf	Influent
L	Mixed liquor
li	Lipids
P	Permeate
pr	Proteins
pro	Propionate / Propionate degraders
su	Monosaccharides
va	Valerate
W	Water
WS	Waste sludge
X	Particulate

References

1. Odriozola, M.; Morales, N.; Vázquez-Padín, J.R.; Lousada-Ferreira, M.; Spanjers, H.; van Lier, J.B. Fouling Mitigation by Cationic Polymer Addition into a Pilot-Scale Anaerobic Membrane Bioreactor Fed with Blackwater. *Polymers (Basel).* **2020**, *12*, 2383, doi:10.3390/polym12102383.
2. Mara, D.; Horan, N.; Hao, O.J. *Handbook of Water and Wastewater Microbiology*; 2003; ISBN 9780124701007.
3. Zhao, X.; Ma, J.; Ma, H.; Gao, D.; Lv, X.; Zhang, J. Adsorptive removal of vinyl polymer tanning agents from aqueous solution using wastewater activated sludge. *Desalin. Water Treat.* **2016**, *57*, 11422–11432, doi:10.1080/19443994.2015.1042064.
4. Horvath, A.E.; Lindström, T.; Laine, J. On the indirect polyelectrolyte titration of cellulosic fibers. Conditions for charge stoichiometry and comparison with ESCA. *Langmuir* **2006**, *22*, 824–830, doi:10.1021/la052217i.
5. Moreau, A.A.; Ratkovich, N.; Nopens, I.; van der Graaf, J.H.J.M. The (in)significance of apparent viscosity in full-scale municipal membrane bioreactors. *J. Memb. Sci.* **2009**, *340*, 249–256, doi:10.1016/j.memsci.2009.05.049.
6. Geilvoet, S. The Delft Filtration Characterisation method Assessing membrane bioreactor activated sludge filterability, Delft University of Technology, The Netherlands, 2010.
7. Durán, F. Modelación matemática del tratamiento anaerobio de aguas residuales urbanas incluyendo las bacterias sulfatorreductoras. Aplicación a un biorreactor anaerobio de membranas, Universitat Politècnica de València, 2013.
8. Batstone, D.J.; Keller, J.; Angelidaki, I.; Kalyuzhnyi, S. V.; Pavlostathis, S.G.; Rozzi, A.; Sanders, W.T.; Siegrist, H.; Vavilin, V.A. *Anaerobic Digestion Model No. 1 (ADM1)*; IWA Publishing: London, 2002; ISBN 1900222787, 9781900222785.
9. Guo, H.; Oosterkamp, M.J.; Tonin, F.; Hendriks, A.; Nair, R.; van Lier, J.B.; de Kreuk, M. Reconsidering hydrolysis kinetics for anaerobic digestion of waste activated sludge applying cascade reactors with ultra-short residence times. *Water Res.* **2021**, *202*, 117398, doi:10.1016/j.watres.2021.117398.
10. Elmitwalli, T.A.; van Leeuwen, M.; Kujawa-Roeleveld, K.; Sanders, W.; Zeeman, G. Anaerobic biodegradability and digestion in accumulation systems for concentrated black water and kitchen organic-wastes. *Water Sci. Technol.* **2006**, *53*, 167–175, doi:10.2166/wst.2006.247.
11. Wendland, C. Anerobic Digestion of Blackwater and Kitchen Refuse, Technischen Universität Hamburg-Harburg, 2008.
12. Feng, Y. Calibration and Verification of a Mathematical Model for the Simulation of Blackwater/Biowaste Digestion (MSc thesis), Hamburg University of Technology, 2004.
13. Tugtas, A.E.; Tezel, U.; Pavlostathis, S.G. An extension of the Anaerobic Digestion Model No. 1 to include the effect of nitrate reduction processes. *Water Sci. Technol.* **2006**, *54*, 41–49, doi:10.2166/wst.2006.524.
14. Metcalf, L.; Eddy, H.P.; Tchobanoglous, G.; Burton, F.L.; Stensel, H.D. *Wastewater Engineering: Treatment and Reuse*; 4th ed.; McGraw Hill: New York, 2002;
15. Sander, R. Compilation of Henry's law constants (version 4.0) for water as solvent. *Atmos. Chem. Phys.* **2015**, *15*, 4399–4981, doi:10.5194/acp-15-4399-2015.
16. Gorini, D.; Choubert, J.-M.; le Pimpec, P.; Heduit, A. Concentrations and fate of sugars, proteins and lipids during domestic and agro-industrial aerobic treatment. *Water Sci. Technol.* **2011**, *63*, 1669–1677, doi:10.2166/wst.2011.334.
17. Odriozola, M.; Lousada-Ferreira, M.; Spanjers, H.; van Lier, J.B. Effect of sludge characteristics on optimal required dosage of flux enhancer in anaerobic membrane bioreactors. *J. Memb. Sci.* **2021**, *619*, 118776, doi:10.1016/j.memsci.2020.118776.

18. Hubbe, M.A.; Wu, N.; Rojas, O.J.; Park, S. Permeation of a cationic polyelectrolyte into mesoporous silica. Part 3. Using adsorption isotherms to elucidate streaming potential results. *Colloids Surfaces A Physicochem. Eng. Asp.* **2011**, *381*, 1–6, doi:10.1016/j.colsurfa.2010.12.052.

19. Benedetti, L.; Claeys, F.; Nopens, I.; Vanrolleghem, P.A. Assessing the convergence of LHS Monte Carlo simulations of wastewater treatment models. *Water Sci. Technol.* **2011**, *63*, 2219–2224, doi:10.2166/wst.2011.453.

20. Sin, G.; Gernaey, K. Data Handling and Parameter Estimation. In *Experimental Methods in Wastewater Treatment*; Loosdrecht, M.C.M. van, P.H. Nielsen, C.M. Lopez-Vazquez, D.B., Eds.; IWA Publishing: London, UK, 2016; pp. 201–234 ISBN 9781780404752.

21. Judd, S.; Judd, C. *The MBR book: principles and applications of membrane bioreactors for water and wastewater treatment*; Judd, Simon; Judd, C., Ed.; Second Edi.; Butterworth-Heinemann: Oxford, 2011; ISBN 9780080966823.

22. Cho, J.; Song, K.G.; Ahn, K.H. The activated sludge and microbial substances influences on membrane fouling in submerged membrane bioreactor: Unstirred batch cell test. *Desalination* **2005**, *183*, 425–429, doi:10.1016/j.desal.2005.05.009.

23. Evenblij, H. Filtration Characteristics in Membrane Bioreactors, Delft University of Technology, The Netherlands, 2006.

24. Lousada-Ferreira, M.; Krzeminski, P.; Geilvoet, S.; Moreau, A.; Gil, J.A.; Evenblij, H.; van Lier, J.B.; van der Graaf, J.H.J.M.; Lier, J.B. Van; Graaf, J.H.J.M. Van Der Filtration characterization method as tool to assess membrane bioreactor sludge filterability-the Delft experience. *Membranes (Basel).* **2014**, *4*, 227–242, doi:10.3390/membranes4020227.

25. Batstone, D.J.; Keller, J.; Blackall, L.L. The influence of substrate kinetics on the microbial community structure in granular anaerobic biomass. *Water Res.* **2004**, *38*, 1390–1404, doi:10.1016/j.watres.2003.12.003.

26. Odriozola, M.; Abraham, E.; Lousada-Ferreira, M.; Spanjers, H.; van Lier, J.B. Identification of the Methanogenesis Inhibition Mechanism Using Comparative Analysis of Mathematical Models. *Front. Bioeng. Biotechnol.* **2019**, *7*, 93, doi:10.3389/fbioe.2019.00093.

27. Lidholm, O.; Ossiansson, E. Modeling Anaerobic Digestion, 2008.

28. Wu, J.; He, C.; Zhang, Y. Modeling membrane fouling in a submerged membrane bioreactor by considering the role of solid, colloidal and soluble components. *J. Memb. Sci.* **2012**, *397–398*, 102–111, doi:10.1016/j.memsci.2012.01.026.

29. Robles, A.; Ruano, M.V.; Ribes, J.; Seco, A.; Ferrer, J. A filtration model applied to submerged anaerobic MBRs (SAnMBRs). *J. Memb. Sci.* **2013**, *444*, 139–147, doi:10.1016/j.memsci.2013.05.021.

30. Robles, A.; Ruano, M. V.; Ribes, J.; Seco, A.; Ferrer, J. Global sensitivity analysis of a filtration model for submerged anaerobic membrane bioreactors (AnMBR). *Bioresour. Technol.* **2014**, *158*, 365–373, doi:10.1016/j.biortech.2014.02.087.

31. Li, X. yan; Wang, X. mao Modelling of membrane fouling in a submerged membrane bioreactor. *J. Memb. Sci.* **2006**, *278*, 151–161, doi:10.1016/j.memsci.2005.10.051.

32. Mannina, G.; Di Bella, G.; Viviani, G. An integrated model for biological and physical process simulation in membrane bioreactors (MBRs). *J. Memb. Sci.* **2011**, *376*, 56–69, doi:10.1016/j.memsci.2011.04.003.

33. Zarragoitia-González, A.; Schetrite, S.; Alliet, M.; Jáuregui-Haza, U.; Albasi, C. Modelling of submerged membrane bioreactor: Conceptual study about link between activated slugde biokinetics, aeration and fouling process. *J. Memb. Sci.* **2008**, *325*, 612–624, doi:10.1016/j.memsci.2008.08.037.
