# Supplementary material for: Optimising the Flux Enhancer Dosing Strategy in a Pilot-Scale Anaerobic Membrane Bioreactor by Mathematical Modelling
Source: Membranes (Basel). 2022 Jan 26;12(2):151. doi: 10.3390/membranes12020151 (PMC8877340; doi:10.3390/membranes12020151)
Supplement: Supplementary file 1 [file membranes-12-00151-s001.zip › Supplementary file s2.zip!/Simulink model S1/Readme.pdf]

Supplementary material

Optimising the flux enhancer dosing strategy in a pilot-scale anaerobic membrane bioreactor by mathematical modelling

Magela Odriozola*, Jules B. van Lier, Henri Spanjers

Department of Water Management, Delft University of Technology, Stevinweg 1, 2628 CN Delft, the Netherlands.

*Corresponding author: m.odriozolaarbiza@tudelft.nl.

Simulink model S1: Simulink® implementation of control tools for flux enhancer dosing to the pilot AnMBR

The Simulink® file *SimulationEnvironment.slx* contains the implementation of the control tools and integrated model described in Section 3.5. The simulation uses the parameter values in *Parameters.mat* and the initial conditions in *InitialConditions.mat*.

To run the simulation, open the file *SimulationEnvironment.slx* in Simulink®. Select the control tool using the manual switches. Select the simulation time, in seconds, and press *Run*.
